# Integrative transcriptomic analysis uncovers the microRNA-centric regulation of Japanese encephalitis virus infection in porcine trophoblast cells

**DOI:** 10.1080/21505594.2026.2690825

**Published:** 2026-06-17

**Authors:** Juan Yang, Jiayao Jiang, Jiajia Gong, Linjie Zhang, Chenxi Li, Xiaocheng Bao, Hairui Fan, Chaohui Dai, Yanhua Li, Shuai Chen, Ming-An Sun

**Affiliations:** a Institute of Comparative Medicine, College of Veterinary Medicine, Yangzhou University, Yangzhou, China; b College of Animal Science and Technology, Yangzhou University, Yangzhou, China; c Institute of Animal Science, Jiangsu Academy of Agricultural Sciences, Nanjing, China; d Joint International Research Laboratory of Important Animal Infectious Diseases and Zoonoses of Jiangsu Higher Education Institutions, Yangzhou, China; e Jiangsu Co-Innovation Center for Prevention and Control of Important Animal Infectious Diseases and Zoonosis, Joint International Research Laboratory of Agriculture and Agri-Product Safety of Ministry of Education of China, Interdisciplinary Center for Zoonotic Diseases and Biosafety, Yangzhou University, Yangzhou, China

**Keywords:** Japanese encephalitis virus, porcine trophoblast cell, microRNA, immune, RNA-seq

## Abstract

Japanese encephalitis virus (JEV) is a mosquito-borne zoonotic virus with pig as its major amplifying host. Despite that JEV can persistently infect the placentae of pregnant sows and cause severe reproductive failures, the molecular responses and potential regulators of JEV infection in pig placenta remain unclear. Using porcine trophectoderm (pTr) cells as model, we investigated the transcriptomic changes during JEV infection, with a special focus on microRNA-centric regulation over immune genes and JEV infection. To avoid currently poor microRNA annotation for pig, we first performed small RNA-seq to achieve refined microRNA annotation, with hundreds of novel microRNAs annotated. After confirming that JEV can efficiently infect pTr cells and induce severe cytopathic effects, we conducted routine and small RNA-seq to determine the changes of mRNA and microRNA genes during JEV infection. We identified >1000 differentially expressed genes and found JEV-induced genes are tightly associated with antiviral immune responses. Moreover, we identified 94 JEV-affected microRNAs, including many with their roles on viral infection still unclear. Focusing on JEV-affected microRNAs, we constructed a microRNA–mRNA negative regulatory network, with multiple hub microRNAs and their targets inspected. Among these microRNAs, we experimentally validated that ssc-miR-149 and ssc-miR-483 both can inhibit JEV replication in pTr cells, with the well-recognized immune suppressor SOCS1 validated as a direct target of ssc-miR-149. Overall, this study comprehensively characterized the microRNA-centric regulation of JEV infection in pTr cells, which provides mechanistical insights into JEV infection in pig placenta and will facilitate the development of novel therapies against JEV infection.

## Introduction

Japanese encephalitis virus (JEV), a mosquito-borne zoonotic virus, is the leading cause of Japanese encephalitis (JE) which threatens human health in Asia and Oceania [1–3]. JEV is a single-stranded, positive-sense RNA virus belonging to the genus *Flavivirus*, which also includes dengue, West Nile, and Zika viruses [3]. Since it was first isolated from the brain of a fatal human encephalitis case in Tokyo in 1934 [4], JEV has an estimated worldwide annual incidence of 69,000 or even more with a 20–30% mortality rate [3,5]. JEV is maintained in a transmission cycle between mosquito vectors, reservoir hosts (waterbirds), and amplifying hosts. Despite the high seroprevalence rates in multiple mammalian species (*e.g*., cattle, dogs, goats, and rodents), pigs are recognized as the only (or at least the most important) amplifying host of JEV [4,6–8]. Indeed, JEV can even achieve vector-free transmission among pigs [9]. Surprisingly, unlike the neurological phenotype in dead-end hosts such as humans and horses [3,10–12], JEV infection in pig primarily causes reproductive disorders, including abortion, stillbirth, and mummified piglets [13–15]. Notably, JEV can persist in the endometrium of infected sows and cause infection of adjacent placentas, subsequently infecting the fetuses [16].

The placenta is a transient organ of eutherian mammals, which not only facilitates the maternal–fetal exchange of nutrients/wastes but also serves as a critical immunological barrier to protect the embryo from infection [17–19]. Unlike highly invasive homochorial placenta in primates and rodents, pigs possess a noninvasive diffuse epitheliochorial placenta [20,21]. Even though mammalian placentae have evolved highly effective strategies to protect the fetus from viruses [17,19,22], there are still a few viruses that can infect placenta and cause pregnancy disorders, such as Zika (also belongs to flavivirus) for human and JEV for pigs [16,23]. Apart from JEV, several other types of viruses – including porcine parvovirus (PPV), porcine reproductive and respiratory syndrome virus (PRRSV), and porcine circovirus type 2 (PCV2) – are also reported to infect trophoblast cells and/or cause reproductive failure [24–27]. Although these viruses cause severe reproductive disorders in sows, the molecular mechanisms underlying their pathogenesis – including JEV which is focused by this study – still remain poorly understood.

Numerous host-derived factors are involved in the regulation of viral infection, among which microRNA (miRNA) represents one class of important regulatory molecules. MiRNAs are endogenous single-stranded non-coding RNAs of ~ 22 nucleotides in length, which can regulate gene expression at the post-transcriptional level [28–30]. In animals, miRNA causes the degradation and/or translation inhibition of target mRNA by pairing the 3’UTR with its seed region [29]. Many host miRNAs are involved in infection processes of different viruses, such as the extensively studied influenza A virus and human immunodeficiency virus [31,32]. Recent studies also identified a number of host miRNAs that regulate JEV infection [33]. For example, miR-19b-3p and miR-15b promote JEV-induced inflammation by targeting RNF11 and RNF125, which are negative regulators of NF-κB and RIG-I signaling [34,35]. There are also many miRNAs that affect viral replication directly, such as miR-33a-5p which inhibits JEV replication by targeting EEF1A [36]. Conversely, miR-301a inhibits interferon (IFN) production by suppressing the production of SOCS5, IRF1, and NKRF, hence promotes JEV replication [37,38]. In addition, the NS3 helicase of JEV can impair pre-miR-466d function, thereby evading the antiviral response and facilitating its replication [39]. Apart from miRNAs, many protein-coding genes have been identified to regulate JEV infection as well [40–42]. Although efforts have been made to understand how host miRNAs regulate JEV infection, previous studies have primarily focused on the central nervous systems of humans or mice. In contrast, the role of miRNAs in regulating JEV infection of the placentae for pigs – the most important amplifying host of JEV – remains to be explored.

In this study, we initially conducted computational annotation to expand the current porcine miRNA repertoire. After characterizing the replication kinetics of JEV in porcine trophectoderm (pTr) cells, we integrated transcriptomic data to identify the miRNAs and their mRNA targets that potentially modulate JEV infection, and further validated the function of multiple miRNAs in regulating JEV infection. This study represents a comprehensive characterization of the miRNA-centric regulation of JEV infection in pTr cells and provides mechanistical insights into the capacity of JEV infection in the porcine placenta.

## Results

### Genome-wide characterization of the miRNAs expressed in pig placenta

**Figure 1.**
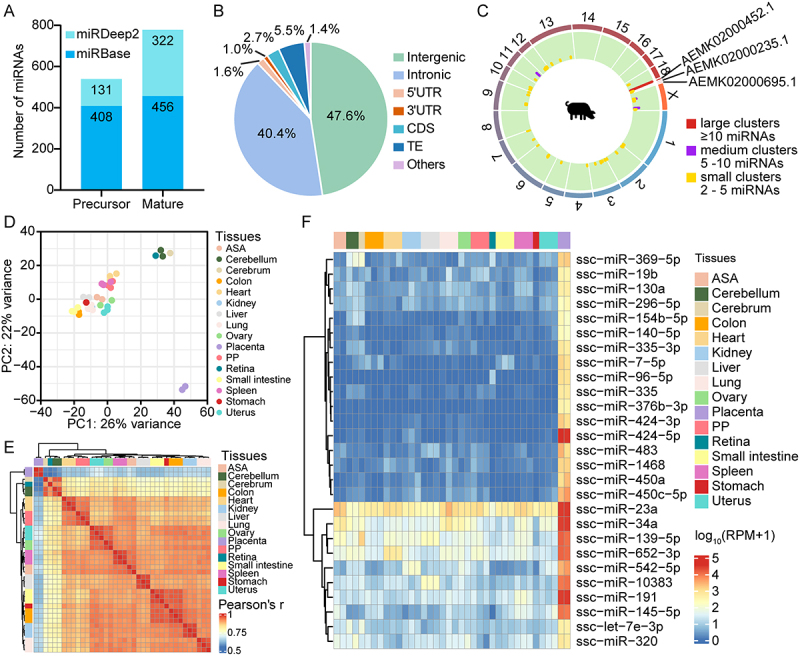
Genome-wide annotation and transcriptomic profiling of porcine miRNAs. (A) The numbers of known porcine miRNAs documented in miRBase and novel porcine miRNAs identified using miRDeep2 in this study. (B) The distribution of porcine precursor miRNA genes across various genomic regions. (C) Circos diagram illustrating the sizes and genomic distribution of porcine miRNA clusters. The outermost ring displays individual chromosomes and scaffolds (only those containing miRNA cluster distribution were shown); the inner ring represents distinct miRNA clusters. The length and color of the lines indicate the size of each cluster, defined as the number of contained miRNAs. (D, E) PCA and hierarchical clustering analyses of different porcine tissues according to miRNA expression. The top 400 miRNAs with the highest expression variance were used for PCA analysis. ASA: abdominal subcutaneous adipose, PP: pectoralis profundus. (F) Heatmap showing the porcine miRNAs with placenta-enriched expression, which are defined as those with significantly higher expression in placenta compared to all other tissues.

The miRNAs are still poorly annotated in pig relative to model species like human and mouse (**Figure S1A**), which precludes their comprehensive investigation. Therefore, we first refined the annotation of pig miRNAs by generating small RNA-seq (sRNA-seq) data of placental tissues and pTr cells (**Figure S1B**, **Table S1**). In total, we identified 539 precursor and 778 mature miRNAs (Figure 1(A)), including 131 and 322 novel ones that are absent from miRBase [43]. Among these novel precursor miRNAs, 53 are conserved given that they match precursor miRNAs of other species, while the remaining are pig-specific (**Table S1**). We further examined their genomic distribution and found most miRNAs localize in introns and intergenic regions, which resembles the patterns for human and mouse miRNAs (Figure 1(B), S1B&C, Table S2). Previous studies indicate that miRNAs frequently originate from transposable elements (TEs), which are mobile DNA elements abundant in animal genomes [44–46]. We inspected their overlap with TEs and found that 5.5% of pig miRNAs are also derived from TEs (Figure 1(B), **Table S2**). Moreover, pig miRNAs frequently form clusters, but unlike large clusters such as the primate-specific C19MC and the rodent-specific C2MC [47,48], they predominantly appear as small clusters (Figure 1(C), S1D, Table S3). Notably, we identified a single large cluster from the scaffold AEMK02000452.1 (Figure 1(C), Table S3), which is homologous to the human C14MC cluster and is conserved among eutherian mammals [49,50].

Given that this study focuses on placenta, we further characterized the pig miRNAs specifically expressed in placenta. For this purpose, we conducted an integrative analysis with the sRNA-seq data of term placenta and other pig tissues generated in this study or retrieved from public resources [51]. Principal component analysis (PCA) and hierarchical clustering demonstrate that the miRNA expression profile of the placenta is distinct from that of other analyzed tissues (Figure 1(D,E)). Of note, apart from placenta, neural tissues (*e.g*., cerebrum, cerebellum, and retina) also form a distinct cluster (Figure 1(D,E)). By differential expression analysis of placenta against other tissues, we identified 27 miRNAs showing placenta-enriched expression, including several striking ones that are expressed exclusively in placenta (Figure 1(F)). The improved annotation of pig miRNAs (particularly those highly expressed in placenta) can facilitate subsequent analysis on the miRNA-centric roles of JEV infection in pig placenta.

### JEV can efficiently infect pTr cells and cause severe cytopathic effects

While porcine placenta was reported to be susceptible to JEV infection [16], the *in vitro* model suitable for studying JEV infection in pig placenta remains to be assessed. Here, we examined JEV infection in the pTr cell line, which is a widely used trophoblast cell line initially established from the trophectoderm of a day 12 porcine conceptus [52]. We infected pTr cells with 1, 5, and 10 multiplicity of Infection (MOI) of JEV, and then inspected the phenotype and molecular changes at 24 and 48 hours post-infection (hpi), respectively.

**Figure 2.**
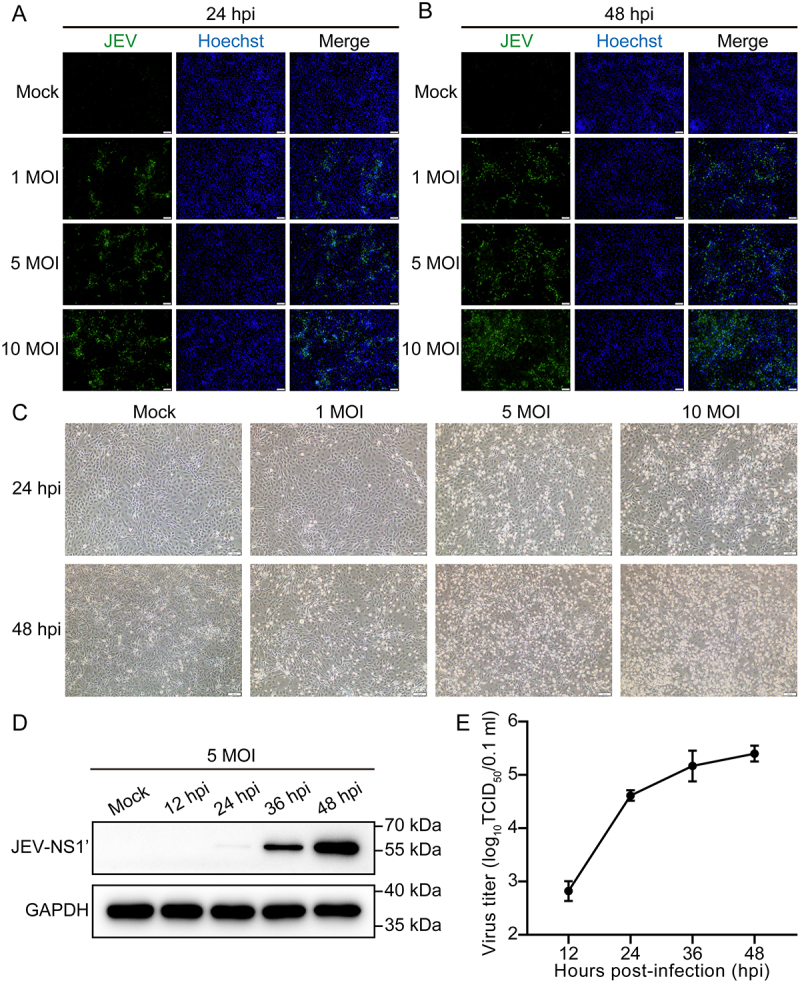
Evaluation of the infection efficiency of JEV in pTr cells (A, B) Indirect immunofluorescence images to visualize the infection of pTr cells by JEV under varying durations of infection and MOI conditions. Mouse-derived anti-JEV serum served as the primary antibody here. (C) Cytopathic effects in pTr cells following JEV infection at various time points and MOI. (D) Expression dynamics of the JEV NS1’ protein in pTr cells during prolonged infection time. Total cellular proteins were harvested from different groups of pTr cells, including mock control and those infected with JEV (MOI = 5) at 12, 24, 36, and 48 hpi, respectively. (E) Changes in virus titers in the culture supernatants of pTr cells infected with JEV at different time points post-infection. pTr cells were infected with JEV at an MOI of 5, the timing of virus infection and collection of culture supernatant was consistent with that described in Figure 2D. Virus titers in the culture supernatants were detected by 50% tissue culture infective dose (TCID_50_) assay. Data are mean ± SD, *n* = 3.

Our immunofluorescence imaging result suggests that JEV can efficiently infect pTr cells at 1 MOI within 24 hours, and the amount of infected cells increased with the escalation of the infection dose and the prolongation of the infection time (Figure 2(A,B)). Importantly, JEV infection can induce severe cytopathic effect in pTr cells, with high proportions of detached cells observed at an MOI of 5 at 24 hpi and MOI of 1 at 48 hpi (Figure 2(C)). In consistent with the trend of JEV infection, the numbers of detached pTr cells also increase in a dose- and time-dependent manner (Figure 2(C)). We also confirm the highly efficient infection and cytopathic effects of JEV in BHK-21 cells (**Figure S2**), which is a baby hamster kidney cell line known to be highly susceptible to JEV infection [53]. However, we assume pTr – but not BHK-21 – is a more suitable model for studying JEV infection in porcine placenta given its close relationship to placenta. To better evaluate the progress of JEV infection, we performed Western blotting with NS1’ antibody and confirmed that viral proteins accumulated continuously as the infection time prolongs (Figure 2(D)). By viral titer assay, we also confirmed that the amount of virus released into the culture supernatant continuously increased until 48 hpi (Figure 2(E)). Together, JEV can efficiently infect pTr cells and cause evident cytopathic effects; therefore, pTr cells can be a suitable *in vitro* model for studying JEV infection in pig placenta.

### JEV infection can trigger strong immune response in pTr cells

**Figure 3.**
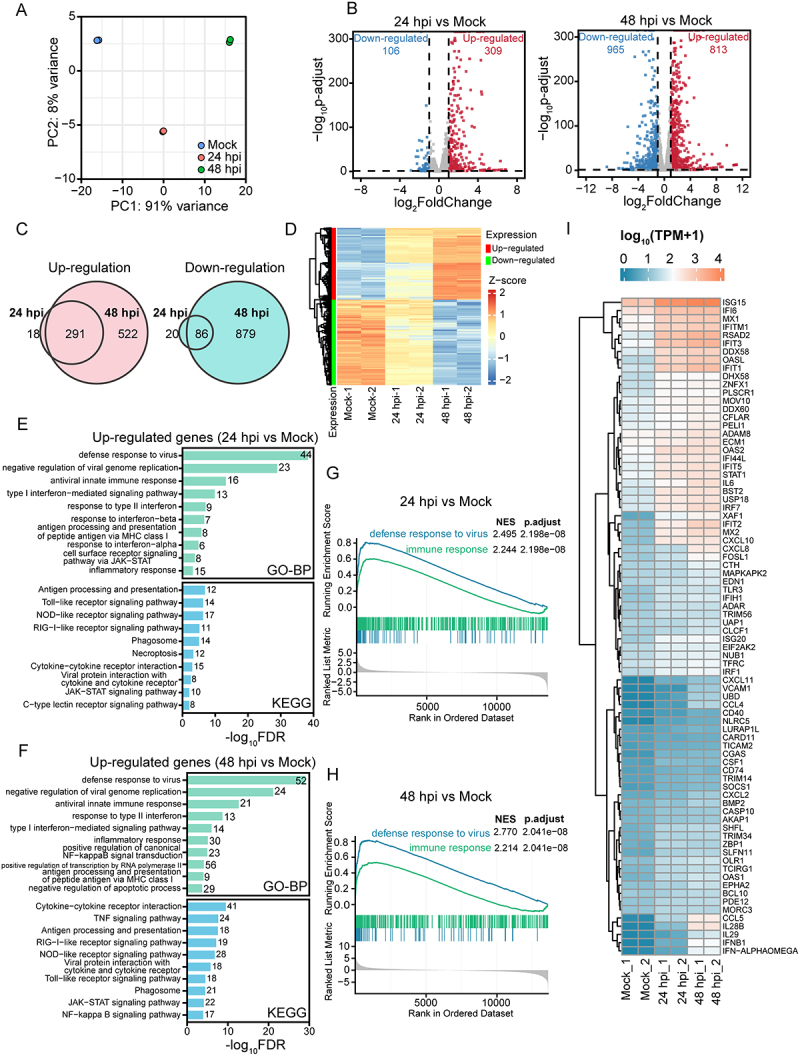
Global mRNA expression profiles of pTr cells following JEV infection. (A) Relationship of all samples based on the PCA results with RNA-seq data. Top 1000 genes with the highest expression variance were used for analysis. (B) Volcano plots depicting the differential expression analysis results in infected vs. mock-infected pTr cells. Genes with up-regulated expression are highlighted in red, while those with down-regulated expression are highlighted in blue. (C) Venn diagram showing the number of intersect genes exhibiting up-regulated and down-regulated expression at 24 hpi and 48 hpi compared to the mock-infected pTr cells. (D) Expression profiles of the DEGs identified between 48 hpi JEV-infected and mock-infected pTr cells. The expression pattern of involved DEGs at 24 hpi was also shown. (E, F) Enriched GO and KEGG terms for up-regulated genes in the 24 hpi and 48 hpi JEV-infected compared to the mock-infected pTr cells. Only the top ten terms from “biological process” category and KEGG pathway are shown. The numeric value displayed at the terminus of each bar represents the count of genes enriched for the corresponding functional term or pathway. (G, H) GSEA plot of defense response to virus and immune response at 24 hpi and 48 hpi compared to the mock-infected. (I) Heatmap presenting the expression of several genes associated with antiviral immunity in JEV-infected and mock-infected pTr cells. JEV infection was performed with MOI = 5 for all experiments relevant to this figure.

After confirming the efficient infection in pTr cells, we further determined the changes of gene expression in response to JEV infection. We choose to use 5 MOI for all following experiments, given that at this infection dose: 1) JEV can achieve both efficient infection rate and maintain favorable cell status; 2) the viral protein production and titer increase progressively in a time-dependent manner during infection. We first performed routine RNA-seq for pTr cells infected with JEV at 5 MOI for 24 and 48 hours, which are compared against the mock-infected group. PCA analysis confirms the reliability of the RNA-seq data for replicated samples and indicates the time-dependent transcriptomic dynamics during infection (Figure 3(A)). Then, we determined the differentially expressed genes (DEGs) after JEV infection. Compared with the mock-infected group, we identified 415 DEGs at 24 hpi, including 309 up-regulated and 106 down-regulated genes (Figure 3(B)). At 48 hpi, the number of up- and down-regulated genes increased to 813 and 965, respectively (Figure 3(B)). Further visualization of the expression profiles of all DEGs confirmed their time-serial expression changes during infection (Figure 3(C,D)). Specifically, most DEGs exhibit a similar trend of alteration at 24 and 48 hpi, even though higher degree of changes usually occur at 48 hpi (Figure 3(D)).

After revealing the transcriptomic changes during JEV infection, we further determined the functional relevance of the identified DEGs. Gene Ontology (GO) and Kyoto Encyclopedia of Genes and Genomes (KEGG) enrichment analyses indicate that the JEV-induced genes at 24 and 48 hpi are both significantly associated with antiviral immunity, such as type I IFN-mediated signaling pathway, toll-like receptor signaling pathway, and antigen processing and presentation (Figure 3(E,F)). Gene set enrichment analysis (GSEA) further confirms that anti-viral defense and immune response are among the most enriched terms in infected groups compared with the mock-infected group (Figure 3(G,H)). Closer inspection further demonstrates the JEV-induced expression of dozens of immune genes (Figure 3(I)), such as multiple IFNs (*e.g*., *IL29*, *IL28B*, *IFNB1*, and the Laurasiatheria-specific *IFN-ALPHAOMEGA*) and IFN-stimulated genes (*e.g*., *MX1*, *MX2*, and *OAS1*). We also examined the functions of genes that were up-regulated exclusively at 48 hpi and found they were primarily associated with the apoptotic process (**Figure S3A**), in line with the cytopathic effects during infection. In contrast, the down-regulated genes are mainly associated with energy metabolism at 24 hpi and cell adhesion at 48 hpi (**Figure S3 B, C**), likely also linked to the JEV-induced cytopathic effects (Figure 2(C)). Together, our transcriptomic data suggest that JEV infection can trigger a strong immune response in pTr cells.

### The miRNA-centric transcriptomic dynamics induced by JEV infection in pTr cells

**Figure 4.**
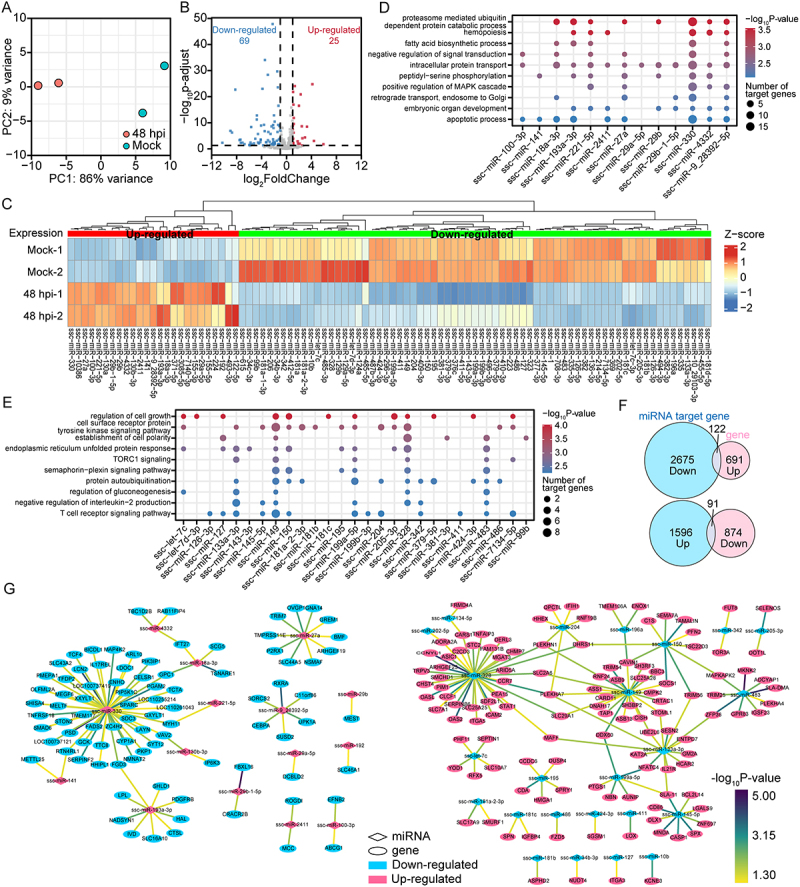
Global miRNA expression profiling and miRNA–mRNA interaction network analysis of pTr cells following JEV infection.

After characterizing the JEV-induced gene response with routine RNA-seq, we further generated matched sRNA-seq data to dissect the roles of miRNAs during JEV infection in pTr cells. PCA analysis demonstrates that the global miRNAome could be remarkably altered after JEV infection (Figure 4(A)). Compared with the mock-infected group, we identified 25 up- and 69 down-regulated miRNAs at 48 hpi (Figure 4(B,C)). Among them, we noticed that several miRNAs (*e.g*., miR-204, miR-150, miR-155, and miR-145-5p) have been previously linked to the infection of JEV or other viruses [54–57], despite that none of these studies are carried out in trophoblast cells. Importantly, we also identified many differentially expressed miRNAs with their roles during JEV infection completely unexplored (Figure 4(C)). Moreover, 27 of the 94 differentially expressed miRNAs are newly annotated in this study (**Table S1**), suggesting the advantage of using improved annotation for pig miRNAs. Interestingly, five differentially expressed miRNAs – including ssc-miR-130a, ssc-miR-483, ssc-miR-335, ssc-miR-335-3p, and ssc-miR-424-3p – are expressed exclusively in placenta (Figure 1F), implying their potential placenta-specific roles. To achieve more functional insights, we predicted the miRNA‑targeted genes and then performed GO enrichment analysis. Overall, the targets for up-regulated miRNAs are related to protein ubiquitination, MAPK signal cascade, embryonic development, and apoptotic process, while those for down-regulated miRNAs are associated with endoplasmic reticulum unfolded protein response, protein autoubiquitination, semaphorin–plexin signaling pathway, and IL2 production (Figure 4(D,E)).

(A) Relationship of all samples based on PCA results with sRNA-seq data. Top 100 miRNAs with the highest expression variance were used for analysis. (B) Volcano plots depicting differentially expressed miRNAs at 48 hpi vs mock-infected. MiRNAs with up-regulated expression are highlighted in red, while those with down-regulated expression are highlighted in blue. (C) Expression profiles of the differentially expressed miRNAs identified between 48 hpi and mock‑infected samples. (D, E) Enriched GO terms for target genes of up-regulated and down-regulated miRNAs at 48 hpi compared to the mock-infected. Only ten representative terms from “Biological Process” category are shown. (F) Venn diagram showing the number of intersections between the target genes of down-regulated miRNAs and up-regulated genes (upper panel) at 48 hpi compared to mock-infected samples, as well as the number of intersections between the target genes of up-regulated miRNAs and down-regulated genes (lower panel) at 48 hpi compared to the mock-infected samples. (G) The miRNA–mRNA interaction networks in JEV-infected pTr cells. The left panel shows the significant interactions of the up-regulated miRNA-down-regulated gene pairs, and the right panel shows the significant interactions of the down-regulated miRNA-up-regulated gene pairs.

Given that miRNAs negatively regulate their target genes, we further screened for the miRNAs which may regulate JEV-affected mRNAs by simultaneously considering the expression of paired miRNAs and mRNAs. In total, 91 target genes of up-regulated miRNAs are down-regulated, while 122 target genes of down-regulated miRNAs are up-regulated (Figure 4(F)). Subsequently, we constructed a negative regulatory network for JEV-affected miRNAs and mRNAs (Figure 4(G)), which shows that one miRNA usually links to multiple mRNAs, and *vice versa*. Strikingly, down-regulated (but not up-regulated) miRNAs tend to target many immune-related mRNAs that are induced by JEV infection (Figure 4(G)), suggesting the loosed repression by these miRNAs may at least partially contribute to the observed immune activation. We further inspected the hub miRNAs in the constructed network and found many of them have previously been reported to regulate the infection process of various viruses (Figure 4(G)). For instance, previous studies suggest that miR-204 inhibits PRRSV replication in porcine alveolar macrophages [55], miR-328-3p enhances PEDV replication in IPEC-J2 cells [58], and miR-133a, let-7c, and miR-150 suppress Dengue virus activity [59–61]. Meanwhile, we uncover several miRNAs (*e.g*., miR-330, miR-149, miR-483) – including a few porcine-specific miRNAs – that have not been linked to viral infection so far (Figure 4(G), Table S1). Together, we not only identify many miRNAs known to regulate the viral infection, but also pinpoint a list of novel miRNAs which may regulate JEV infection in pig placenta and are valuable candidates for further studies.

### ssc-miR-149 and ssc-miR-483 inhibit JEV replication in pTr cells

Previous studies suggest that viral infection can affect the expression of specific miRNAs in host cells, and these miRNAs in turn can influence the viral life cycle [62,63]. Given that dozens of miRNAs with JEV-affected expression in pTr cells are identified (Figure 4(C)), it is intriguing to further examine if these miRNAs are capable of influencing JEV infection. For this purpose, we selected the two miRNAs ssc-miR-149 and ssc-miR-483 – whose expressions are repressed following JEV infection (Figure 4(C)), as candidates for experimental validation. To be noted, unlike miR-149 which is a well-annotated miRNA conserved among mammals, ssc-miR-483 is a miRNA novelly annotated in the pig genome by our computational analysis (**Table S1**). However, multiple evidence suggest that ssc-miR-483 is a *bona fide* pig miRNA: 1) the secondary structure of the ssc-miR-483 precursor is highly stable, and the duplex of mature miRNAs exhibits a 3’ overhang of 2 nt (**Figure S4A**) [43]; 2) miR-483 is conserved in mammals, and its precursor gene is located within the penultimate intron of the imprinted gene *IGF2* (**Figure S4B**; 3) ssc-miR-483 shows high sequence similarity (and identical seed sequence) to its orthologues in other mammals (**Figure S4C**); 4) the expression level of ssc-miR-483 was significantly reduced following knockdown of *DICER1*, which encodes Dicer, a key endoribonuclease for miRNA biosynthesis (**Figure S4D**). Interestingly, our data suggest that ssc-miR-483 is expressed exclusively in porcine placenta (Figure 1(F)), indicating that it may have certain intrinsic placenta-specific roles.

**Figure 5.**
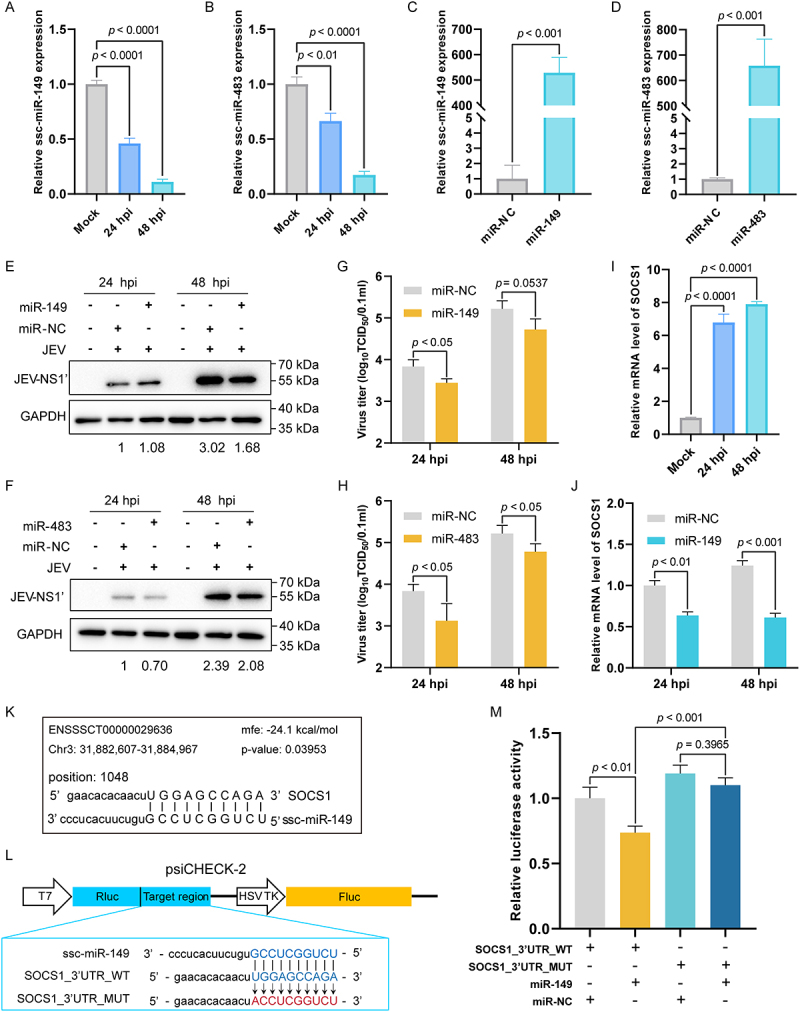
Experimental validation of the roles of ssc-miR-149 and ssc-miR-483 in repressing JEV replication (A,B) RT-qPCR analysis for ssc-miR-149 (A) and ssc-miR-483 (B) expression (normalized to U6) in pTr cells during JEV infection. Data are mean ± SD, *n* = 3. Two-tailed *P* values were analyzed using Student’s t-test. (C, D) RT-qPCR analysis for ssc-miR-149 and ssc-miR-483 expression (normalized to U6) after transfection with their respective mimics for 48 hours. Data are mean ± SD, *n* = 3. Two-tailed *P* values were analyzed using Student’s t-test. (E–H, J) PTr cells were transfected with ssc-miR-149 and ssc-miR-483 mimics (miR-149 and miR-483) and negative control (miR-NC) for 24 hours and then infected with JEV at an MOI of 5. The cells and culture supernatants were harvested at 24 and 48 hpi, respectively. (E,F) According to the previous method, JEV NS1’ protein levels were determined by Western blotting. At each time point, the three treatment groups from left to right include: 1) blank control group, with neither miRNA mimics transfection nor JEV infection; 2) cells transfected with negative control (miR-NC) for 24 hours and then infected with JEV; 3) cells transfected with miRNA mimics (miR-149/miR-483) for 24 hours and then infected with JEV. The values at bottom represent the relative expression level of JEV NS1’ protein. The band intensities were quantified and normalized to GAPDH, with the expression level of NS1’ in the miR-NC transfection group set as 1. (G, H) JEV titers were determined by TCID_50_ assay in BHK-21 cells. Data are mean ± SD, *n* = 3. Two-tailed *P* values were analyzed using Student’s t-test. (I) RT-qPCR analysis for SOCS1 expression (normalized to GAPDH) in pTr cells during JEV infection. Data are mean ± SD, *n* = 3. Two-tailed *P* values were analyzed using Student’s t-test. (J) SOCS1 mRNA levels were determined by RT-qPCR and normalized to GAPDH. Data are mean ± SD, *n* = 3. Two-tailed *P* values were analyzed using Student’s t-test. (K) Visualization of the target site of ssc-miR-149 in the 3’UTR of *SOCS1*. The Ensembl transcript ID, the genomic position of the 3’UTR, and the starting position of the ssc-miR-149 binding site within the 3’UTR are indicated. The *p*-value and minimum free energy (MFE) were computed using RNAhybrid. (L) Schematic diagram showing the construction of the *SOCS1* 3’UTR dual-luciferase reporter plasmid containing the predicted binding site of ssc-miR-149. All the mutated nucleotides of the target site are indicated in red. (M) Comparison of luciferase activity for pTr transfected with ssc-miR-149 mimics (miR-149) or negative control (miR-149) along with a WT or mutated *SOCS1* 3’UTR luciferase reporter plasmid. The luciferase activity was assessed 36 hours after transfection. Data are presented as relative Renilla luciferase activities normalized to firefly luciferase. Data are mean ± SD, *n* = 3. One-way ANOVA was used for statistical analysis. JEV infection was performed with MOI = 5 for all experiments relevant to this figure.

Reverse transcription and quantitative PCR (RT-qPCR) demonstrates that both ssc-miR-149 and ssc-miR-483 are gradually down-regulated following JEV infection (Figure 5(A,B)), in consistent with their expression pattern according to our sRNA-seq data. To test whether they can influence JEV replication, pTr cells were transfected with control miRNA mimic (miR-NC) or the miRNA mimics which effectively overexpress ssc-miR-149 and ssc-miR-483, respectively (Figure 5(C,D)), and then subjected to JEV infection. Following overexpression of ssc-miR-149 or ssc-miR-483, western blotting revealed a remarkable reduction of viral NS1’ protein levels at 24 hpi for ssc-miR-483 and 48 hpi for both miRNAs (Figure 5(E,F)), TCID_50_ assay demonstrated a significant reduction in viral titer within the culture supernatant at 24 hpi for both miRNAs and 48 hpi for ssc-miR-483 (Figure 5(G,H)) and RT-qPCR showed a marked decrease in JEV RNA levels at 24 hpi for ssc-miR-149 and 48 hpi for ssc-miR-483 (**Figure S5**). Therefore, both ssc-miR-149 and ssc-miR-483 can inhibit JEV replication in pTr cells.

Among the predicted target genes of ssc-miR-149, we noticed that suppressor of cytokine signaling 1 (*SOCS1*) is a well-recognized negative regulator of the IFN pathway [64–67]. Moreover, *SOCS1* knockdown in macrophages leads to the suppression of JEV mRNA expression as previously reported [68], indicating its roles in modulating JEV infection. Therefore, we focused on SOCS1 for in-depth analysis. We confirmed that *SOCS1* is significantly up-regulated during JEV infection and shows significantly decreased expression after ssc-miR-149 overexpression (Figure 3(I), 5(I,J)). Closer inspection shows that the pairing between ssc-miR-149 and the 3’UTR binding region of *SOCS1* is highly stable (Figure 5(K)). To confirm whether *SOCS1* is directly regulated by ssc-miR-149, we constructed dual-luciferase reporter plasmids containing the 3’UTR of *SOCS1* harboring either the wild-type or mutated ssc-miR-149 binding region (Figure 5(L)). Our result demonstrates that the overexpression of ssc-miR-149 using miRNA mimics significantly reduces the luciferase activity of the pTr cells transfected with the wild-type (but not the mutant) *SOCS1* 3’UTR plasmid (Figure 5(M)). Therefore, *SOCS1* is validated as a direct target of ssc-miR-149. Taken together, we speculate that ssc-miR-149 may inhibit JEV replication at least partly by suppressing the expression of *SOCS1*.

## Discussion

Pig is the major amplifying host of JEV. Apart from the encephalitis symptom as observed in pig and other animals like human and mouse, increasing evidence suggests that JEV can infect pig placenta and lead to severe pregnancy disorders [12,14,16]. Nevertheless, most current studies on JEV are restrained to its infection in neuronal systems, with the regulatory mechanism of its infection in placenta remains poorly understood. Using pTr cells as an *in vitro* model, we characterized the miRNA-centric regulatory mechanism of JEV infection in pig placenta. From a list of genes which show altered expression after JEV infection, the two miRNAs ssc-miR-149 and ssc-miR-483 are validated to inhibit the replication of JEV in pTr cells; therefore, they may serve as valuable candidates for the prevention or treatment of JEV infection in pig placenta.

One obstacle that needs to be overcome in this study is the poor annotation of miRNAs in pig genome. Although 38,589 precursor and 48,860 mature miRNAs derived from 271 organisms have been released for the miRBase database [43], the number of miRNAs annotated in pig is far less than human and mouse, which precludes a comprehensive study of pig miRNAs. Therefore, we first refined the annotation of pig miRNAs by integrating newly generated sRNA-seq data for porcine placenta and pTr cells. We obtained a list of “high confidence” pig miRNAs, including 539 precursors and 778 mature miRNAs, and impressively, a high proportion (131 precursors and 322 mature miRNAs) of them are novel ones that are absent from miRBase. Taking advantage of the refined annotations, we further determined the pig miRNAs with placenta-specific expression which are then focused for analysis. For the annotated pig miRNAs, we demonstrate that the large cluster located on the scaffold AEMK02000452.1 is homologous to the miRNA cluster on human chr14 (C14MC) and is conserved in eutherian mammals [50]. Notably, of the 35 precursor miRNA genes in this cluster, 23 are newly annotated by our study (**Table S3)** – further highlighting the necessity to use improved pig miRNA annotations. In human, C14MC is embedded in the imprinted *Dlk1-Dio3* domain, with most involved miRNAs grouped into two parts: miR-127/miR-136 cluster and miR-379/miR-410 cluster [50]. However, *DLK1* and *DIO3* are distributed on chr7 and scaffold AEMK02000452.1, respectively, according in the pig reference genome, suggesting that the scaffold AEMK02000452.1 is likely to be a fragment of chr7. Collectively, the refined annotation by our study will facilitate extensive study of the regulatory role of pig miRNAs during JEV infection.

Despite that JEV-induced reproductive failures in pigs have long been recognized [13,14], direct evidence on JEV infection in pig trophoblast cells is still lacking. Therefore, we first assessed the infection capacity of JEV in pTr cells, followed by the inspection of phenotypic and molecular changes after JEV infection. Our experimental data demonstrate that JEV can not only efficiently infect pTr cells, but also cause severe cytopathic effects with prolonged infection time. Hence, our data provide sufficient evidence on JEV infection in pig trophoblasts and suggest that pTr cells are a promising *in vitro* model for studying JEV infection. Subsequently, we performed routine RNA-seq to determine mRNA gene expression changes in pTr cells following JEV infection. We revealed that the antiviral response of pTr cells was activated as early as 24 hours after JEV infection, and the majority of JEV-induced genes are related to the production and function of IFNs, which are a family of cytokines with antiviral activity and consist of three groups: IFN-I (IFN-α and IFN-β), IFN-II (IFN-γ), and IFN-III (IFN-λ) [69,70]. We found that after JEV infection in pTr cells, the induction of IFN-I (mainly *IFNB* and the laurasiatherian-specific *IFN-ALPHAOMEGA*) and IFN-III (*IL29* and *IL28B*) genes is particularly strong. Notably, IFN-I and IFN-III (but not IFN-II) share highly similar signal transduction cascades and also exert similar biological activities [69,71,72]. Interestingly, the trophoblast cells of human and mouse are reported to constitutively produce IFN-III, providing innate antiviral protection at the maternal–fetal interface [22,48,73], yet it is unclear whether the trophoblast cells of other mammals such as pigs also produce IFN-III constitutively. Surprisingly, we demonstrate that IFN-III genes have zero baseline expression level (as measured by TPM value) in pTr cells and become expressed only after JEV infection, suggesting that at least the *in vitro* pTr cells do not constitutively express IFN-III genes. Nevertheless, further evidence is needed regarding whether pig placentae constitutively produce IFN-III *in vivo* for antiviral activity, as observed in human and mouse [22,48]. As for IFN-I signaling pathway, its activation in the fetus and placenta is previously reported to cause embryo abortions and growth restriction in pregnant mice infected with the Zika virus [74], which also belongs to Flavivirus genus as JEV. Further research is desirable to examine where the reproductive failure in pregnant sows infected with JEV is also due to IFN-I signaling activation.

After uncovering the JEV-induced transcriptomic changes as discussed above, we turned our focus to miRNAs for several reasons: 1) recent studies in humans and mice suggest that specific miRNAs contribute to the unique immune properties of trophoblast cells [48,75]; 2) while multiple miRNAs (*e.g*., miR-370, miR-146a, miR-301a) have been reported to regulating JEV infection in neuronal cells [37,76–79]; 3) miRNAs with immune modulatory roles can serve as promising targets for antiviral therapy strategies [80–82]. By integrating our sRNA-seq data, we identified dozens of miRNAs with altered expression in pTr cells after JEV infection. To achieve more mechanistical understanding about the roles of these miRNAs, we constructed a negative regulatory network between differentially expressed miRNA and mRNA genes. In this network, certain miRNAs are known to regulate the viral infection process, whereas the roles of remaining miRNAs are still unclear. Globally, we found that there are much more JEV-repressed miRNAs relative to JEV-induced miRNAs. Given that the subgenomic flavivirus RNA (sfRNA) derived from the 3’UTR of West Nile virus can suppress RNA interference pathway by inhibiting the cleavage of double-stranded RNA by Dicer [83–85], it is highly plausible that such mechanism is also shared by JEV and other flaviviruses.

Among the JEV-affected miRNAs, we experimentally validated that the two candidates ssc-miR-149 and ssc-miR-483 both can inhibit JEV infection in pTr cells. Strikingly, these two miRNAs are both repressed by JEV infection, hinting that by affecting them, JEV may attenuate the antiviral response of pTr cells against their infection. In this sense, those miRNAs can be regarded as a route for JEV to escape the antiviral response from porcine trophoblast cells. Similar strategy has also been reported for JEV (in neuronal cells) and some other viruses [86]. From the mechanistical perspective, we identified *SOCS1* as a target of ssc-miR-149, which may influence the antiviral activity. Among the multiple potential target genes of ssc-miR-149, we chose *SOCS1* for in-depth analysis for several reasons: 1) *SOCS1* is a highly possible target of ssc-miR-149 considering both the bioinformatic prediction result and their reversed expression pattern; 2) JEV infection of pTr cells activates the type I interferon signaling pathway, and SOCS1 (and other SOCS proteins) is a well-recognized negative regulator of the JAK/STAT signaling pathway [67,87]; 3) Host miRNAs can inhibit viral replication by targeting SOCS proteins during the infection of different viruses, including JEV [64–66]; 4) Knockdown of *SOCS1* in macrophages can significantly decrease the viral gene expression of JEV [68], indicating that it is capable to inhibit JEV infection. Given that *SOCS1* is validated as a direct target of ssc-miR-149, we speculate that ssc-miR-149 most likely inhibits JEV replication in pTr cells by targeting *SOCS1*.

We would like to note that this study also has several limitations. First, as this study mainly focuses on the global dynamics and functions of miRNA during JEV infection, we did not conduct in-depth mechanistical investigation into the identified miRNAs and their targets. Currently, we only experimentally confirmed the function of two miRNAs (ssc-miR-149 and ssc-miR-483), and validated the direct regulation of ssc-miR-149 over *SOCS1*. However, we cannot exclude the possibility that other miRNAs and their targets may also participate in the regulation of JEV infection. For example, ZFP36 and MAPKAPK – which are predicted targets of ssc-miR-483 – are also known to assist some viruses in evading the immune response [88,89], and these genes may be valuable candidates for further studies. Second, we did not perform further experiments to validate the function of *SOCS1* in regulating JEV infection, given that its roles in regulating interferon signaling pathway and viral infection (including JEV) have already been reported by numerous previous studies. However, it should be noted that the function of SOCS1 has not been directly examined in pTr yet, and the exact roles of SOCS1 in regulating JEV infection in porcine placenta deserve further investigation.

Overall, this study uncovers the dynamics of miRNAs and mRNAs during JEV infection in pTr cells, and pinpoints a list of miRNAs and their targeted mRNAs with potential roles for regulating JEV infection in pig placenta. Our study not only provides important insights into the miRNA-centric regulation of JEV infection in pig placenta, but also provides valuable candidate targets for developing novel treatment therapies against JEV infection.

## Materials and methods

### Cells and viruses

The pTr cell line was originally maintained by Chaohui Dai’s lab and cultured in DMEM/F-12 (Gibco, 21,331,020) containing 10% FBS (Sigma-Aldrich, F8318), 100 IU/mL penicillin and 100 ug/mL streptomycin (Solarbio, P1400) and 5 ug/mL insulin (Shanghai Yuanye, S24703) at 37 °C in a 5% CO_2_ incubator. BHK-21 cells were maintained by Yanhua Li’s lab and grown in DMEM supplemented with 10% FBS (Sigma–Aldrich, F8318), 100 IU/mL penicillin, and 100 ug/mL streptomycin (Solarbio, P1400) at 37 °C in a 5% CO_2_ incubator. The JEV SD-12 strain (GenBank No. MH753127) used in this study was maintained by Yanhua Li’s lab. All experiments involving JEV were approved by the Institutional Biosafety Committee of Yangzhou University and performed in the biosafety level 2 facility of Yangzhou University

### Reagents and antibodies

Mouse monoclonal anti-NS1’ antibody was purchased from GeneTex (GT1345). Mouse-derived anti-JEV positive serum was a gift from Yanhua Li’s lab of Yangzhou University. Mouse monoclonal anti-GAPDH antibody and HRP-conjugated Goat Anti-Mouse IgG (H+L) antibody were purchased from Proteintech Group (60004–1-Ig, SA00001-1). Anti-mouse IgG (H+L), F(ab’)2 Fragment (Alexa Fluor® 488 Conjugate) antibody was purchased from Cell Signaling Technology (4408S).

The miRNA mimics for ssc-miR-149 and ssc-miR-483 and the siRNA for porcine *DICER1* were purchased from GenePharma. Their sequence details are provided in **Table S4.**

### Plasmid construction

To construct wild-type psiCHECK-2-SOCS1_3’UTR, the fragment containing approximately 200 bp sequences flanking both upstream and downstream of the binding site of ssc-miR-149 on SOCS1 3’UTR was amplified from cDNA derived from pTr cells. The PCR product was digested with NotI-HF (NEB, R3189V) and XhoI (NEB, R0146S), and subsequently cloned into the psiCHECK-2 dual-luciferase reporter vector (GenePharma). For the mutant psiCHECK-2-SOCS1_3’UTR, the inserted fragment was obtained via overlap-extension PCR. All primers used for plasmid construction are listed in **Table S4**.

### Transfection and RT-qPCR

All of the miRNA mimics (50 nM/well) or siRNA (30 nM/well) were transfected into pTr cells in 12-well plates using Lipofectamine™ RNAiMAX (Invitrogen, 13,778,150). For the viral infection experiments, cells were infected with JEV SD-12 at 5 MOI at 24 hours post-transfection. Cells and supernatants from cell cultures were collected 24 hours and 48 hours post-infection. Total RNAs were extracted from cells with RNAiso Plus (TaKaRa, 9109). HiScript III 1st Strand cDNA Synthesis Kit (+gDNA wiper) (Vazyme, R312) was used to reverse transcribe 1 μg of RNAs into cDNAs according to the manufacturer’s instructions. Quantitative real-time PCR analysis was performed in a Quant Gene 9600 real-time PCR System (Bioer, FQD-96C) with Hieff® qPCR SYBR Green Master Mix (YEASEN, 11203ES08). Gene-specific primers are listed in **Table S4**.

To quantify miRNAs, miRNA 1st Strand cDNA Synthesis Kit (by stem-loop) (Vazyme, MR101) was used to reverse transcribe 1 μg of total RNAs into cDNAs according to the manufacturer’s instructions. And miRNA Unimodal SYBR qPCR Master Mix (Vazyme, MQ102) was used for quantitative real-time PCR reactions. The 2^−ΔΔCt^ method was used to calculate the relative expression of the target gene. The expression of mRNAs were normalized to *GAPDH*, while miRNAs were normalized to *U6* snRNA.

### Virus infection and viral titration assay

When pTr cells reach 90%–100% confluence, the viral inoculum is diluted in DMEM/F12 (Gibco, 21,331,020) and added to each well. Following a 2-hour incubation period, the medium is replaced with fresh DMEM/F12 with 2% FBS. For different MOIs of infection, we incubated pTr cells with JEV suspensions at concentrations of 1 MOI, 5 MOI, and 10 MOI, respectively, at the same time, and then collected the cells and supernatants at 24 and 48 hours after infection. For infections of different durations, we conducted the analysis at the same time point. And 12 hours, 24 hours, 36 hours, and 48 hours before the analysis time, we infected the cells with 5 MOI.

The culture supernatants of the infected cells were collected, and 50% tissue culture infective dose (TCID_50_) assay was performed to assess the viral titer as described previously [90]. First, BHK-21 cells were plated into 96-well plates. Once the cells formed a single layer, the indicated infection supernatant was serially diluted tenfold to infect the cells. After about 4 d, the wells with cytopathic effect were counted. The viral titers were determined using the Reed–Muench method.

### Immunoblotting and indirect immunofluorescence assay

Immunoblotting assay was performed as described previously [11]. Briefly, the cells were lysed using RIPA lysis buffer (Epizyme Biotech, PC101) containing protease inhibitor (Beyotime, P1005). After centrifugating, cell lysate supernatant was collected and separated using 12% SDS-PAGE and further transferred onto a PVDF membrane (Merk Millipore, ISEQ00010). The membrane was blocked with 5% skimmed milk in TBST, and then incubated with corresponding primary and secondary antibodies.

For immunofluorescence assay, the cells were first fixed with 4% PFA (YEASEN, 60536ES60) at 4 °C overnight and permeabilized with 0.5% Nonidet (R)P-40 (Sangon Biotech, A600385-0100) at room temperature for 10 min. After being blocked with 5% BSA (Sangon Biotech, A600332-0100) in PBS at room temperature for 1 hour, the cells were subsequently incubated with serum and secondary antibody at room temperature for 1 hour. The cell nucleus was stained with Hoechst (Cell Signaling Technology, 4082S) for 20 min.

### Dual-luciferase reporter assay

For the *SOCS1* 3’UTR luciferase reporter assay, 400 ng of the aforementioned psiCHECK-2 dual-luciferase plasmid and 30 nM ssc-miR-149 mimics (miR-149) or mimics negative control (miR-NC) were co-transfected into pTr cells in 24-well plates using jetPRIME transfection reagent (Polyplus, 101,000,046) according to the manufacturer’s instructions. At 36 hour post-transfection, the activities of Renilla and firefly luciferase were detected using a dual-luciferase reporter assay kit (Vazyme, DL101). The final luciferase activity data are expressed as the Renilla luciferase activity relative to Firefly luciferase activity.

### Placental sample preparation and RNA isolation

Porcine placenta samples were collected from Duroc gilts housed at a swine breeding facility in Jiangsu Province, China. The samples were obtained on-site immediately following the delivery of their first litter. Animal experiments were approved by the Institutional Animal Care and Use Committee of Yangzhou University (IACUC No: SYXK2021-0026). The fetal membranes were subsequently removed from the placental tissues, with similar procedure following previous studies [91,92]. Collected placenta samples were frozen in −80 °C for later use. For total RNA extraction, 100 mg porcine placenta tissue samples were lysed with RNAiso Plus (TaKaRa, 9109) and isolated following manufacturer’s protocol.

### RNA-seq

Total RNA was submitted for library construction by DNBSEQ Eukaryotic Strand-specific mRNA library sample preparation kit (BGI). Each treatment has two biological replicates. RNA-seq libraries were sequenced as 150 bp paired-end reads with DNBSEQ platform (DNBSEQ Technology).

Raw reads were trimmed with Trim Galore v0.6.5. Transcript Per Million (TPM) values were calculated with RSEM v1.3.2 [93]. To perform differential expression analysis, trimmed reads were aligned to the reference genome (Sscrofa11.1 for pig) using STAR v2.7.3 [94], and then obtained gene-level read counts using the *featureCount* function from subread v2.0.0 [95]. At last, differentially expressed genes were identified using DESeq2 v1.42.1 [96] with the cutoff: FDR < 0.05 and |log_2_Foldchange| >1.

### Small RNA-seq

Total RNA was submitted for library construction by UMI small RNA library sample preparation kit (BGI). Each group has two biological replicates. Small RNA-seq libraries were sequenced as 50 bp single-end reads with DNBSEQ platform (DNBSEQ Technology).

Raw reads were trimmed with Trim Galore v0.6.5 to remove reads that contain low-quality bases and with length less than 18 bp or greater than 25 bp. To predict novel miRNA, trimmed reads were aligned to the pig reference genome (Sscrofa11.1) using the *mapper.pl* function of miRDeep2 v0.1.3 [97], and then obtained novel miRNAs with corresponding scores using the *miRDeep2.pl* function of miRDeep2 v0.1.3. We just keep the novel miRNAs with a score greater than 4 as candidate miRNAs. Then, a series of filters are performed to eliminate the false positive candidate miRNAs: 1) Keep precursor miRNAs that can be aligned to the known miRNAs of other species in miRbase v22 [43] using BLAST v2.13.0+ and regard them as conserved miRNAs; 2) For the other candidate miRNAs except those in 1), successively eliminate those aligned to other small RNAs (including rRNA, tRNA, sRNA, and snRNA) and pig mRNA using BLAST v2.13.0+, and the reads of mature miRNA less than 10; 3) retain the candidate precursor miRNAs that have read distributions in both the predicted mature and star sequences as pig‑specific miRNAs [43]. Because miRDeep2 distinguishes the two arms of a precursor miRNA as mature and star sequences, and in both human and mouse, the mature and star sequences are often annotated as the two mature miRNAs of the precursor miRNA. For novel mature miRNA, we keep all the mature and star sequences with reads >10 of the novel precursor miRNAs. For a certain arm of a precursor miRNA that has been annotated but is not annotated itself and has reads >10, we also classified it as a novel mature miRNA. We combined the newly identified novel miRNAs with the pig miRNAs that already annotated in miRBase.

We obtained miRNA-level read counts and Reads Per Million (RPM) using the *quantifier.pl* of miRDeep2 v0.1.3. Differentially expressed miRNAs were identified using DESeq2 v1.42.1 with the cutoff: FDR < 0.05 and |log_2_Foldchange| >1. We also used DESeq2 v1.42.1 to conduct pairwise differential analysis of miRNA expression in porcine placental tissue versus other tissues one by one. Then, we took the intersection of all miRNAs with FDR < 0.05 and log_2_Foldchange > 3 to define the miRNAs enriched expression in porcine placenta.

### Analysis of miRNA clusters

For miRNA clusters analysis, the precursor miRNAs within 10 kb are first merged using bedtools merge of BEDTools v2.29.2 [98]. Then, the number of miRNAs in each merged interval is counted using *windowBed* of BEDTools v2.29.2, and this will yield the miRNA clusters for each species. We determined the localization of miRNAs in the genome using bedtools intersect of BEDTools v2.29.2 to analyze the precursor miRNAs with a minimum, reciprocal overlap of 80% with which genomic elements. The percentage distribution of miRNA across genomic elements for each species is defined as the number of miRNAs overlapping with a given genomic element divided by the total number of miRNAs with genomic coordinates in that species. Due to overlaps between TEs and other genomic elements, the calculated proportion of miRNAs located in non-TE genomic regions excludes those miRNAs that overlap with TEs.

### miRNA target gene prediction

Regarding miRNAs that up-regulated or down-regulated at 48 hour post-infection compared with the mock-infected group, we only conducted target gene prediction for those miRNAs with RPM > 10 in the 48 hours post-infection samples. We used miRanda v3.3a [99] and RNAhybrid v2.1.2 [100] for prediction, and took the intersection of their results as the candidate miRNA target genes.

### Reference genome and annotation

Reference genome of pig (Sscrofa11.1) were downloaded from the Ensembl database (release 108). The precursor and mature miRNAs of pig, human, and mouse were downloaded from miRbase v22 [43]. The BED files showing the positions of precursor miRNA for human and mouse were extracted from the miRNA annotation files downloaded from miRbase v22. The BED file containing the positions of precursor miRNA for pig was obtained by aligning the precursor miRNA sequences to the pig genome using BLAST v2.13.0+. The porcine mRNA sequences were downloaded from UCSC Genome Browser. The rRNA, tRNA, sRNA, and snRNA sequences were downloaded from Rfam 15 [101]. The coding sequence regions, introns, 3’UTR, 5’UTR, and intergenic regions of the pig protein coding genes were extracted from the pig genome annotation file which was downloaded from Ensembl database (release 108). The 3’UTR sequences of porcine protein-coding genes were downloaded using Ensembl BioMart.

### Functional enrichment analysis

GO and KEGG enrichment analyses for DEGs and target genes of differentially expressed miRNAs were performed using DAVID functional annotation tools [102]. Gene set enrichment analysis was performed on the RNA-seq data using clusterProfiler v4.10.1 [103].

### Correlation analysis of differentially expressed miRNAs and mRNAs

We constructed the negative regulatory network of differentially expressed miRNAs and mRNAs between the 48 hpi and the mock-infected group using Cytoscape v3.9.1 [104].

### Statistical analysis and data visualization

All statistical analyses were performed with R statistical programming language (R 4.3.2) or GraphPad Prism 8 software. Statistical testing methods for each result were described in the corresponding figure legends. Heatmaps for gene or miRNA expression clustering analysis were generated using pheatmap v1.0.12 (https://github.com/raivokolde/pheatmap) and ComplexHeatmap v2.18.0 (https://github.com/jokergoo/ComplexHeatmap). PCA analysis and visualization were per formed using the plotPCA function in DESeq2 v1.42.1. The circos diagrams representing miRNA clusters were performed using circlize v0.4.16 [105].

## Supplementary Material

Table S2.xlsx

Figure S2.tif

Table S3.xlsx

Figure S5.tif

Figure S3.tif

Figure S4.tif

Table S4.xlsx

Supplemental figures.docx

Figure S1.tif

Table S1.xlsx

FigShare.zip

## Data Availability

All the data generated in this study have been deposited to NCBI Gene Expression Ominibus database (GEO: https://www.ncbi.nlm.nih.gov/geo) under accession GSE317296. All raw experimental data and images have been deposited to FigShare at https://doi.org/10.6084/m9.figshare.31228216 [106]. All these data are available under the license: CC BY 4.0. The public porcine multi-tissue sRNA-seq data [51] was retrieved from NCBI GEO database under accession GSE162147.
