# Supplementary figures and images for: Integrative transcriptomic analysis uncovers the microRNA-centric regulation of Japanese encephalitis virus infection in porcine trophoblast cells
Source: Virulence. 2026 Jun 17;17(1):2690825. doi: 10.1080/21505594.2026.2690825 (PMC13313263; doi:10.1080/21505594.2026.2690825)

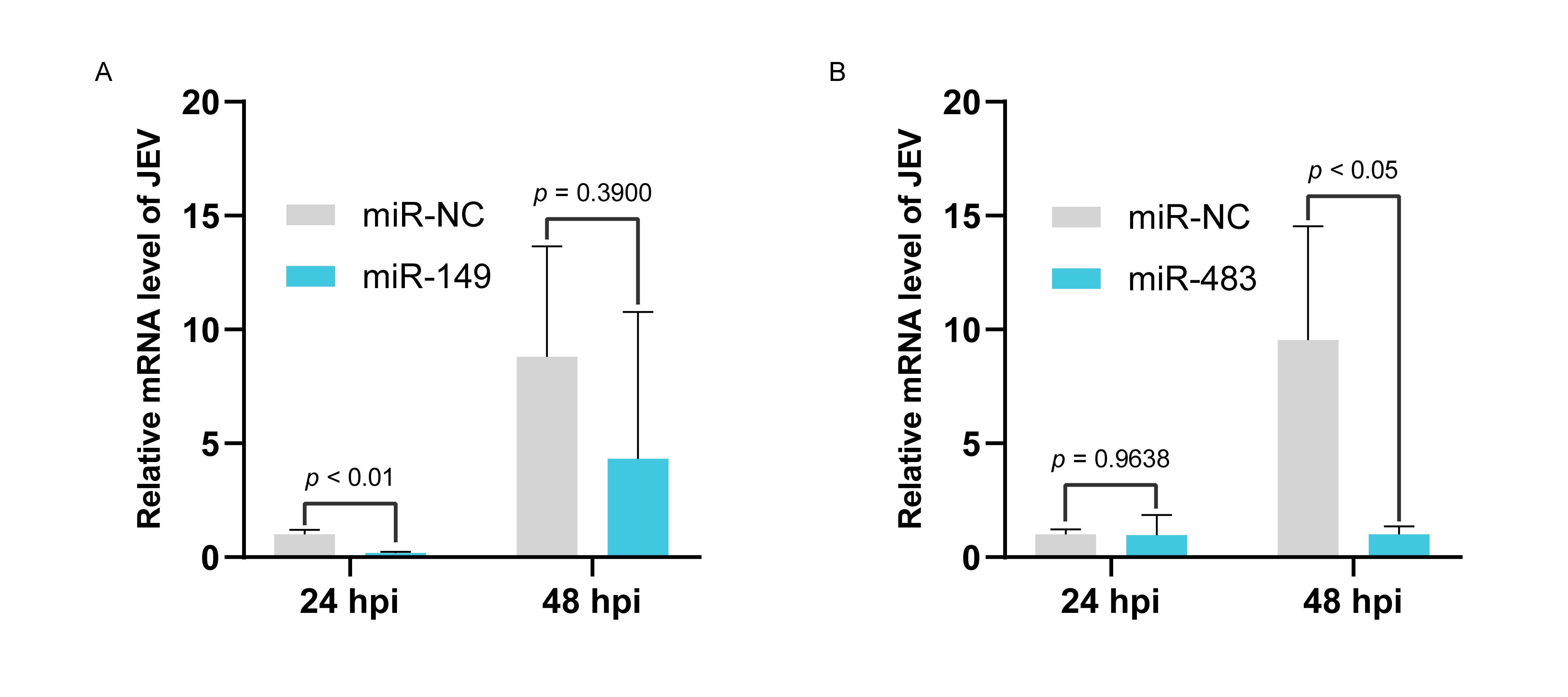

Supplement: Figure S5.tif [file KVIR_A_2690825_SM3836.tif]

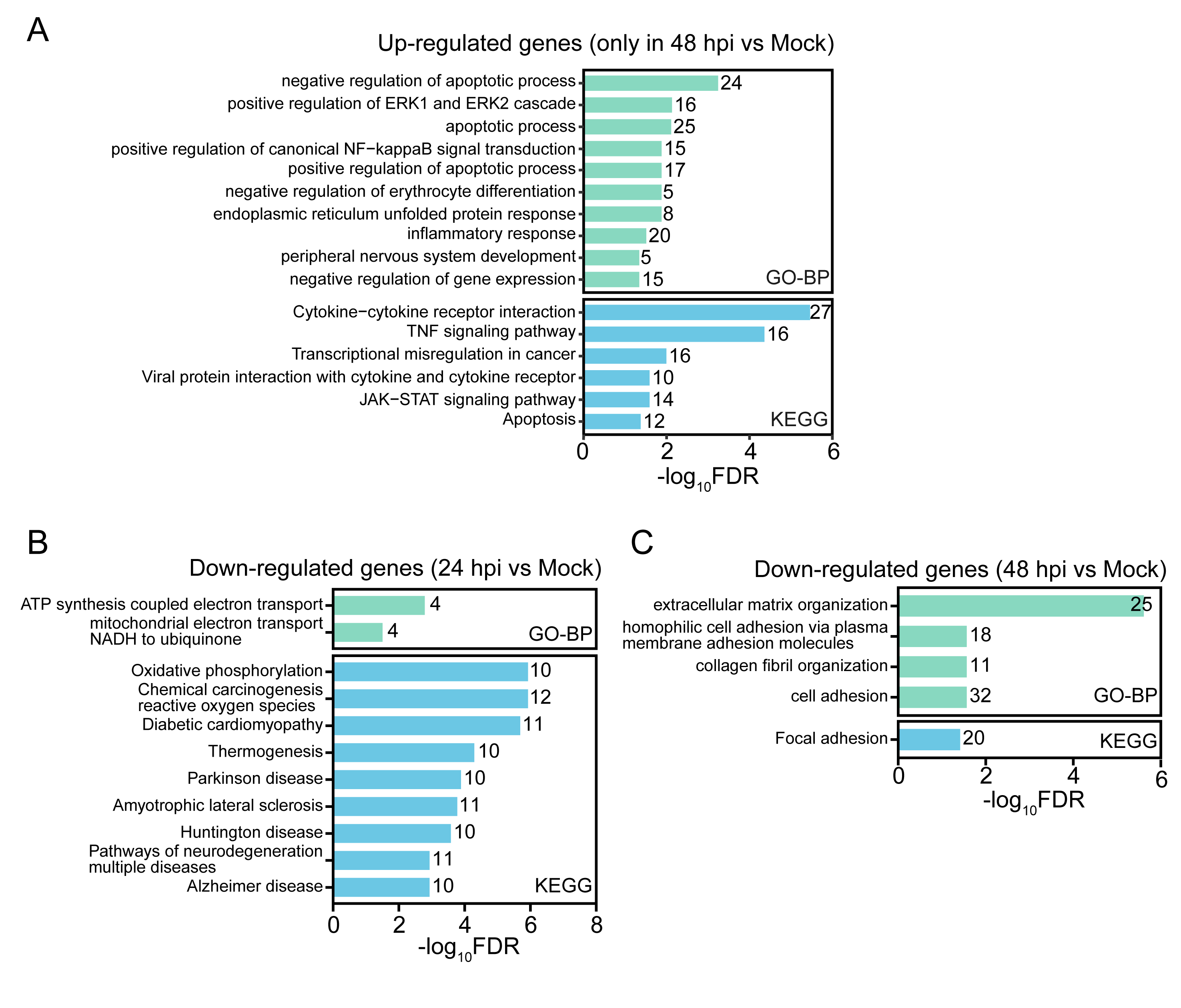

Supplement: Figure S3.tif [file KVIR_A_2690825_SM3835.tif]

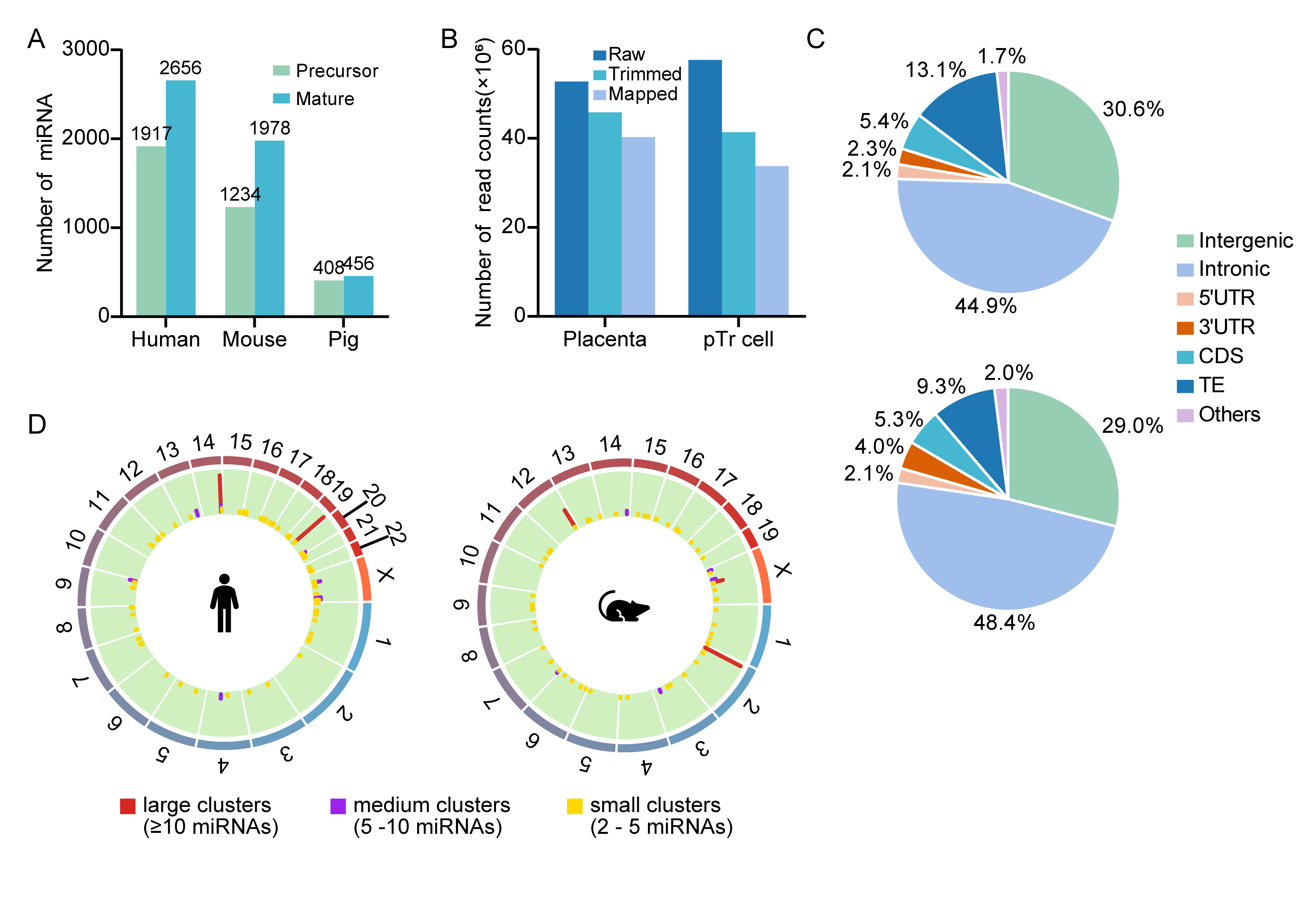

Supplement: Figure S1.tif [file KVIR_A_2690825_SM3831.tif]

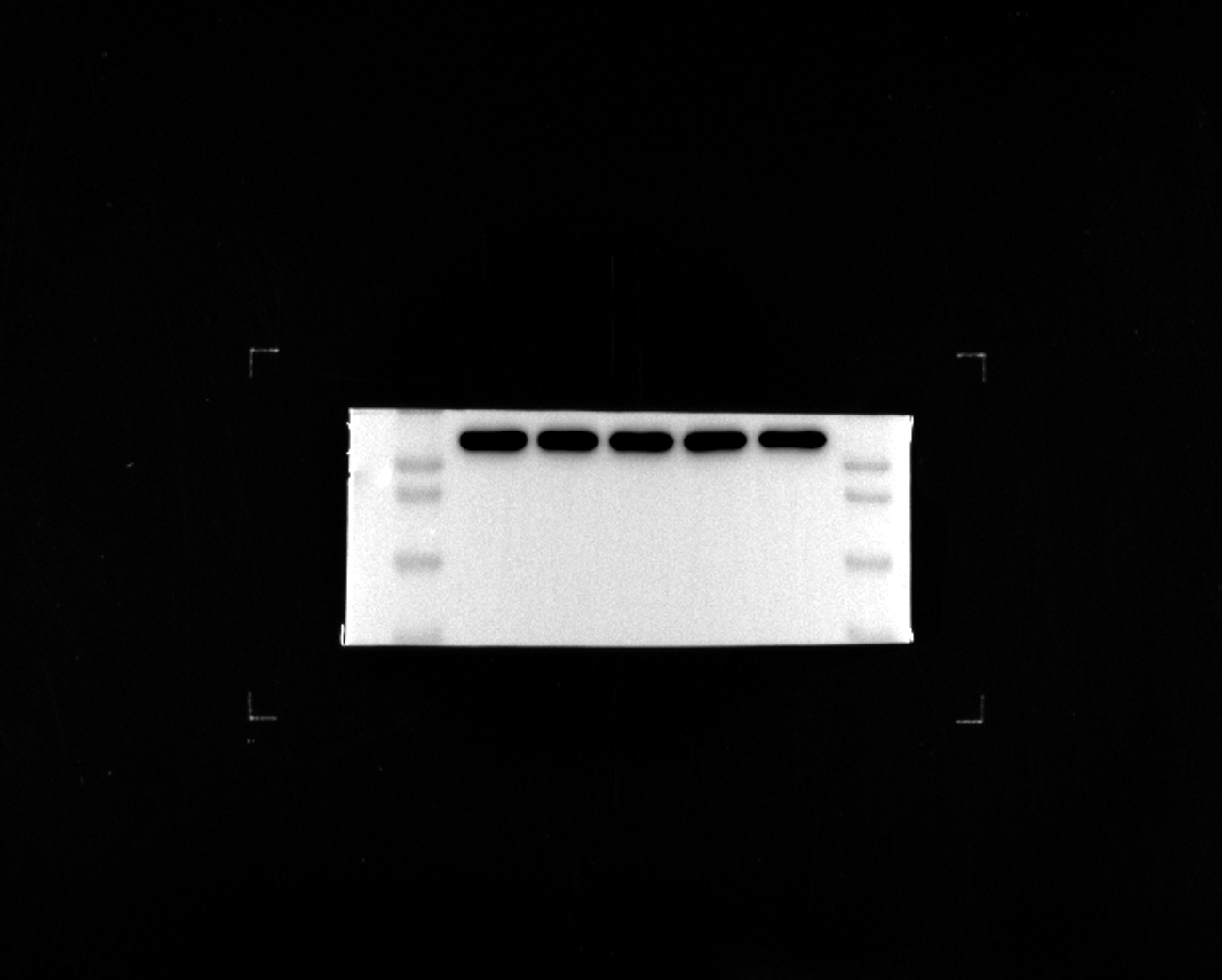

Supplement: FigShare.zip [file KVIR_A_2690825_SM3829.zip › FigShare/Data for figure 2D/GAPDH-10S-M.Tif]

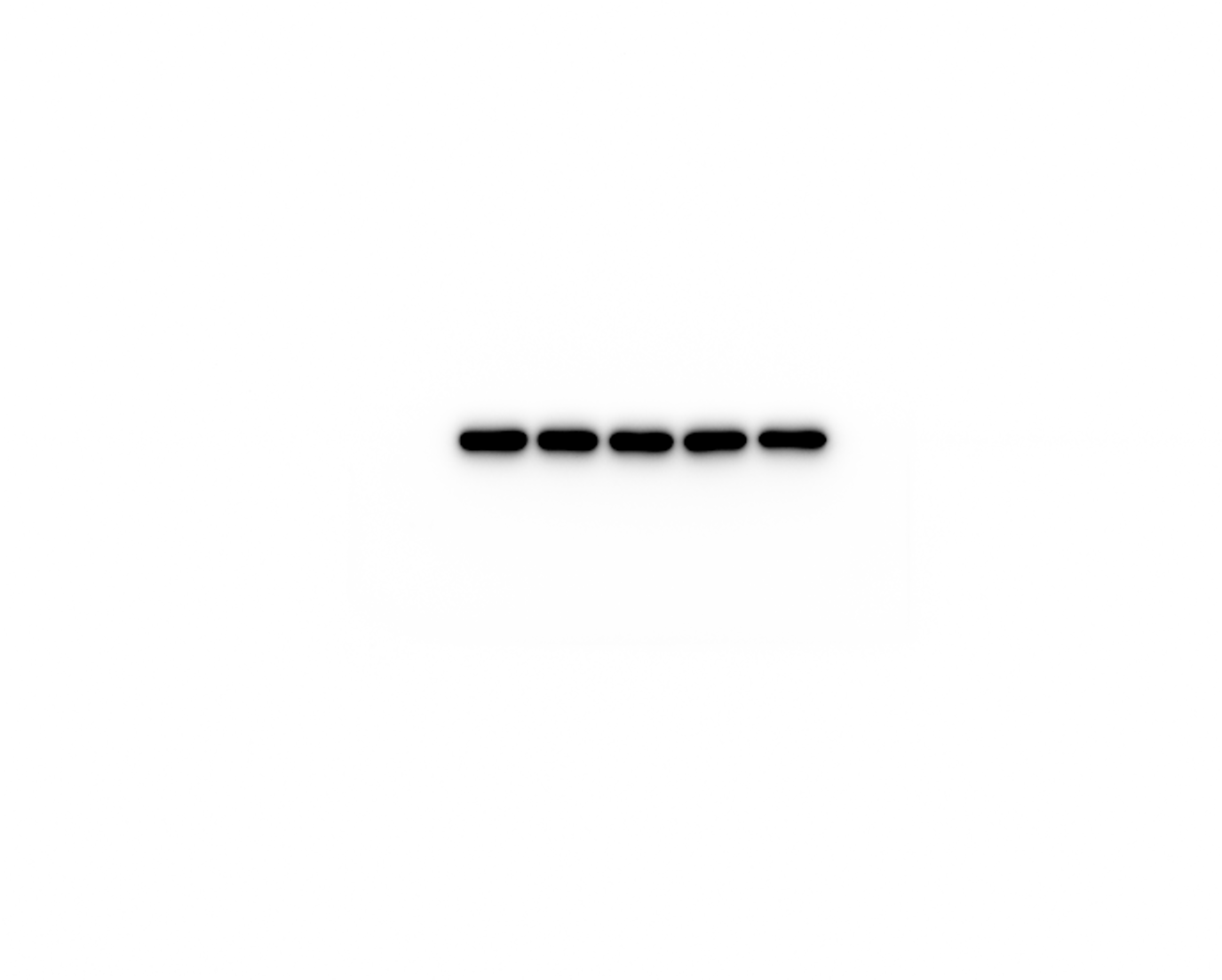

Supplement: FigShare.zip [file KVIR_A_2690825_SM3829.zip › FigShare/Data for figure 2D/GAPDH-10S.Tif]

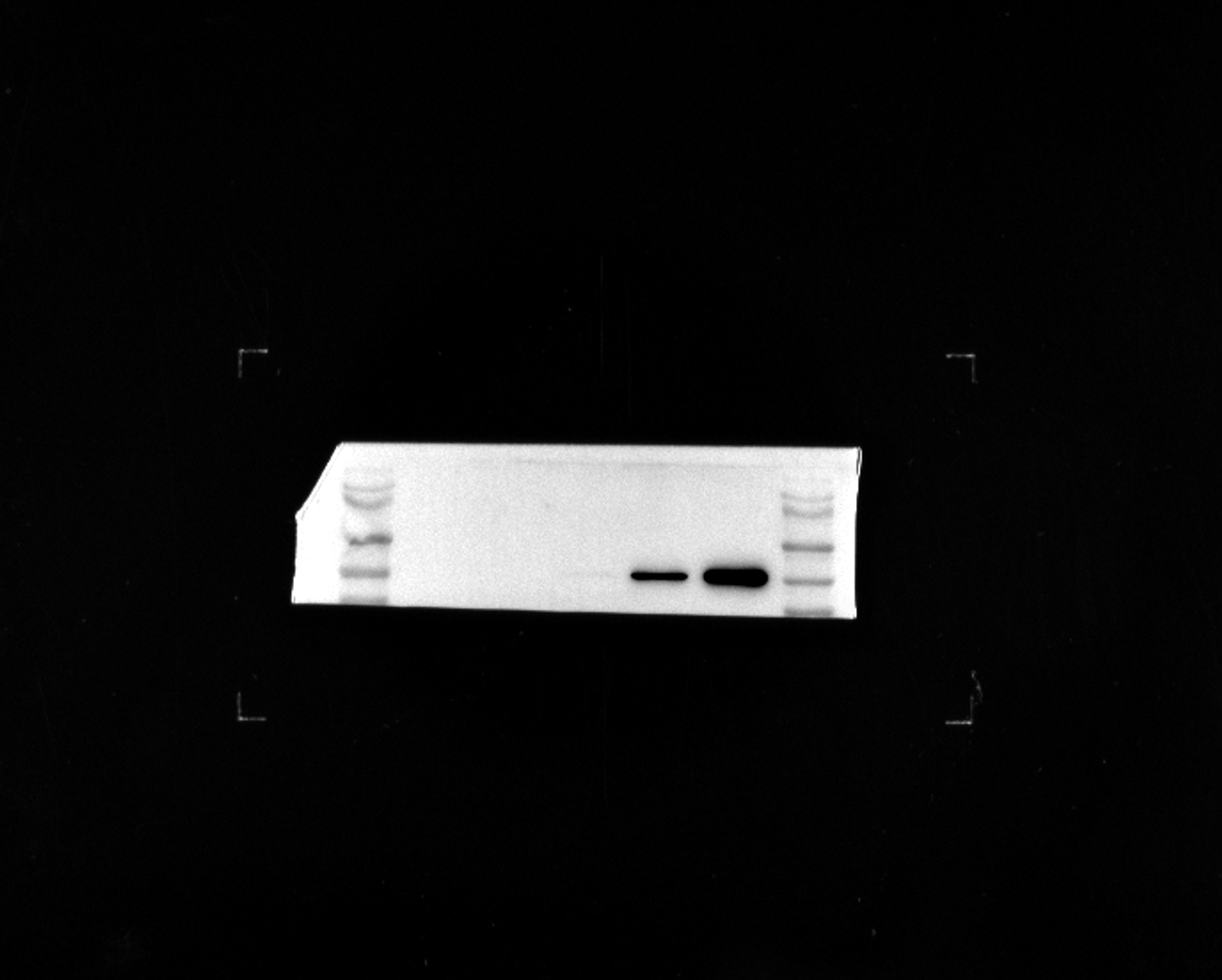

Supplement: FigShare.zip [file KVIR_A_2690825_SM3829.zip › FigShare/Data for figure 2D/ns1'-120s-M.Tif]

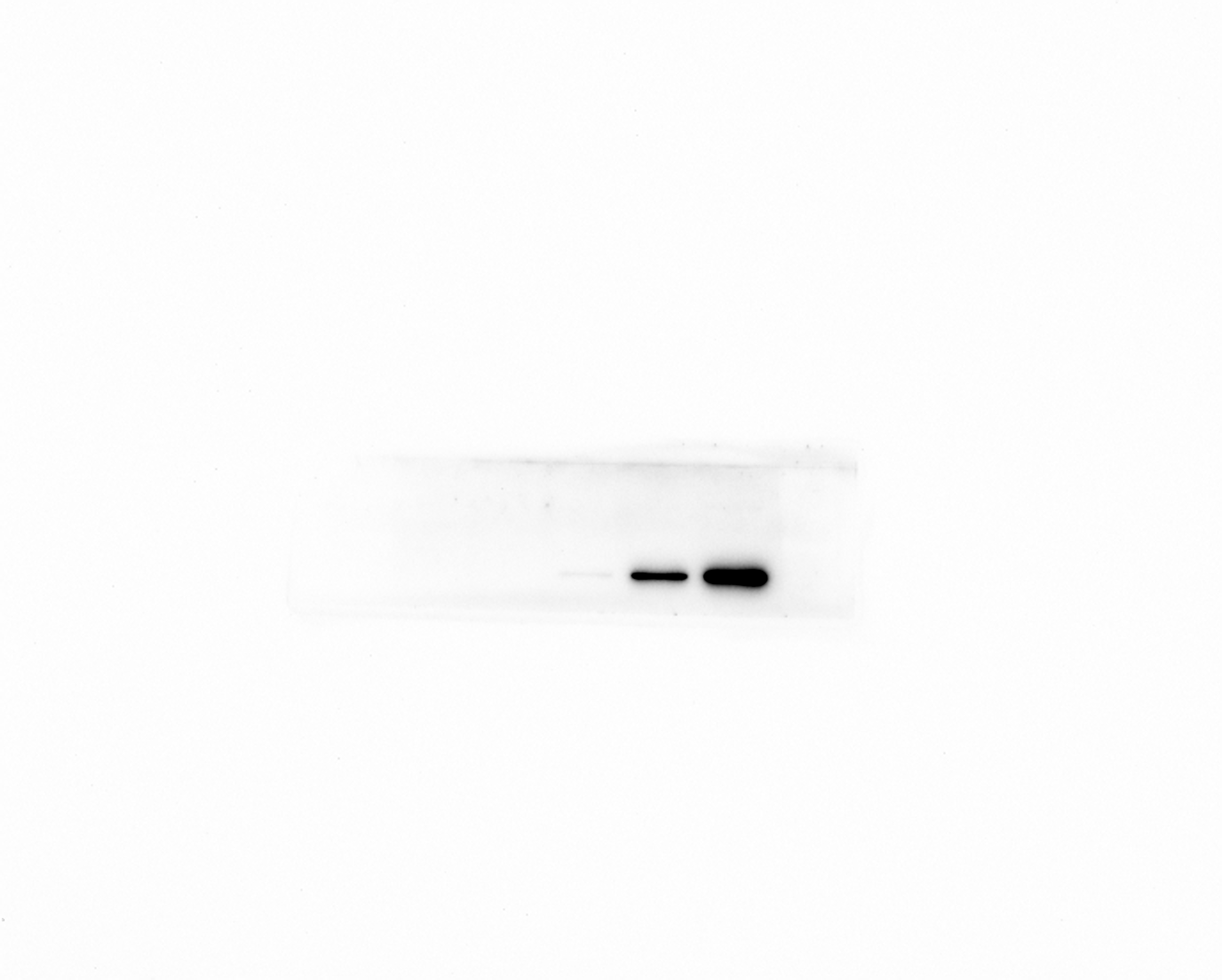

Supplement: FigShare.zip [file KVIR_A_2690825_SM3829.zip › FigShare/Data for figure 2D/ns1'-120s.Tif]

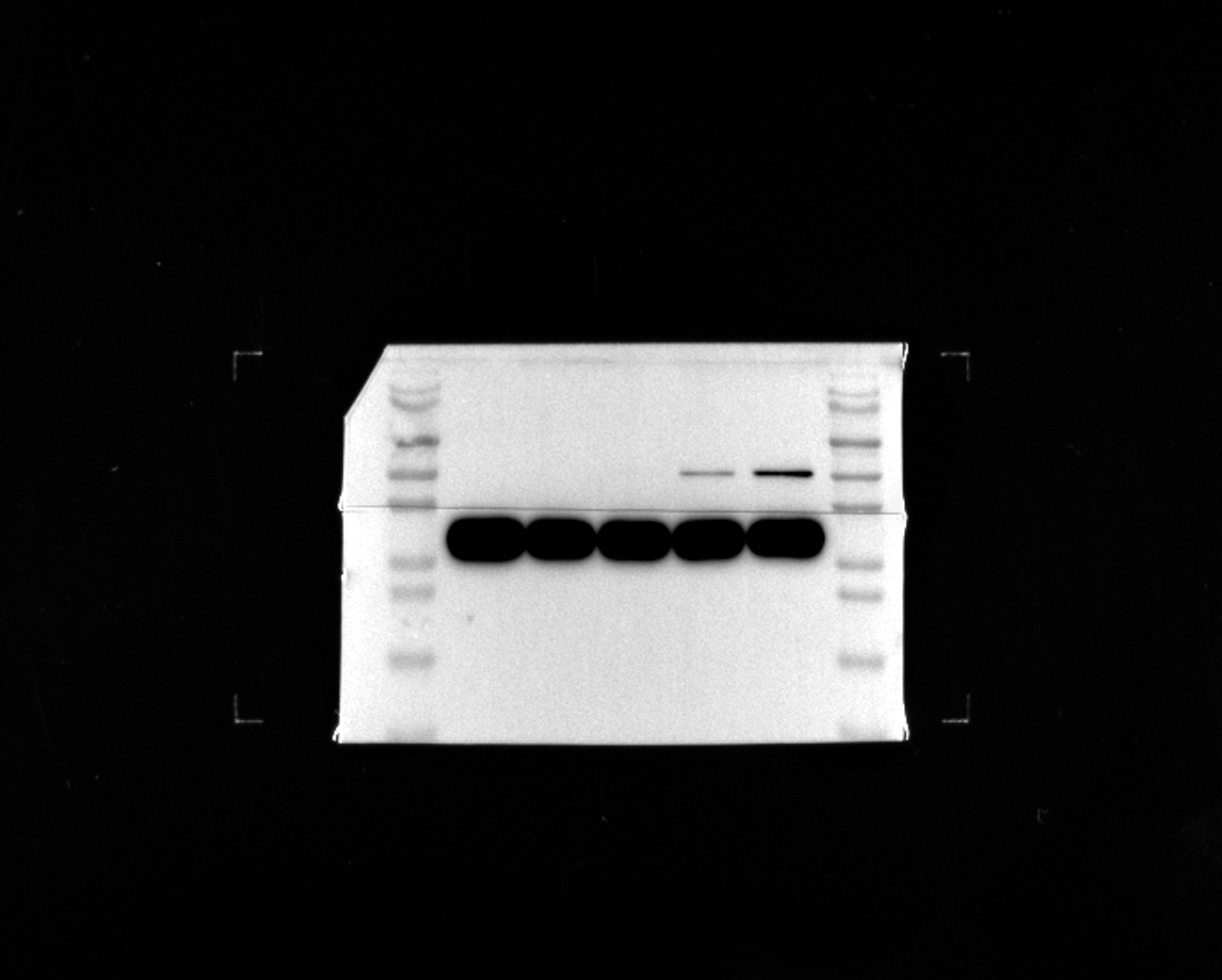

Supplement: FigShare.zip [file KVIR_A_2690825_SM3829.zip › FigShare/Data for figure 2D/ns1'-GAPDH-120S-MERGE-M.Tif]

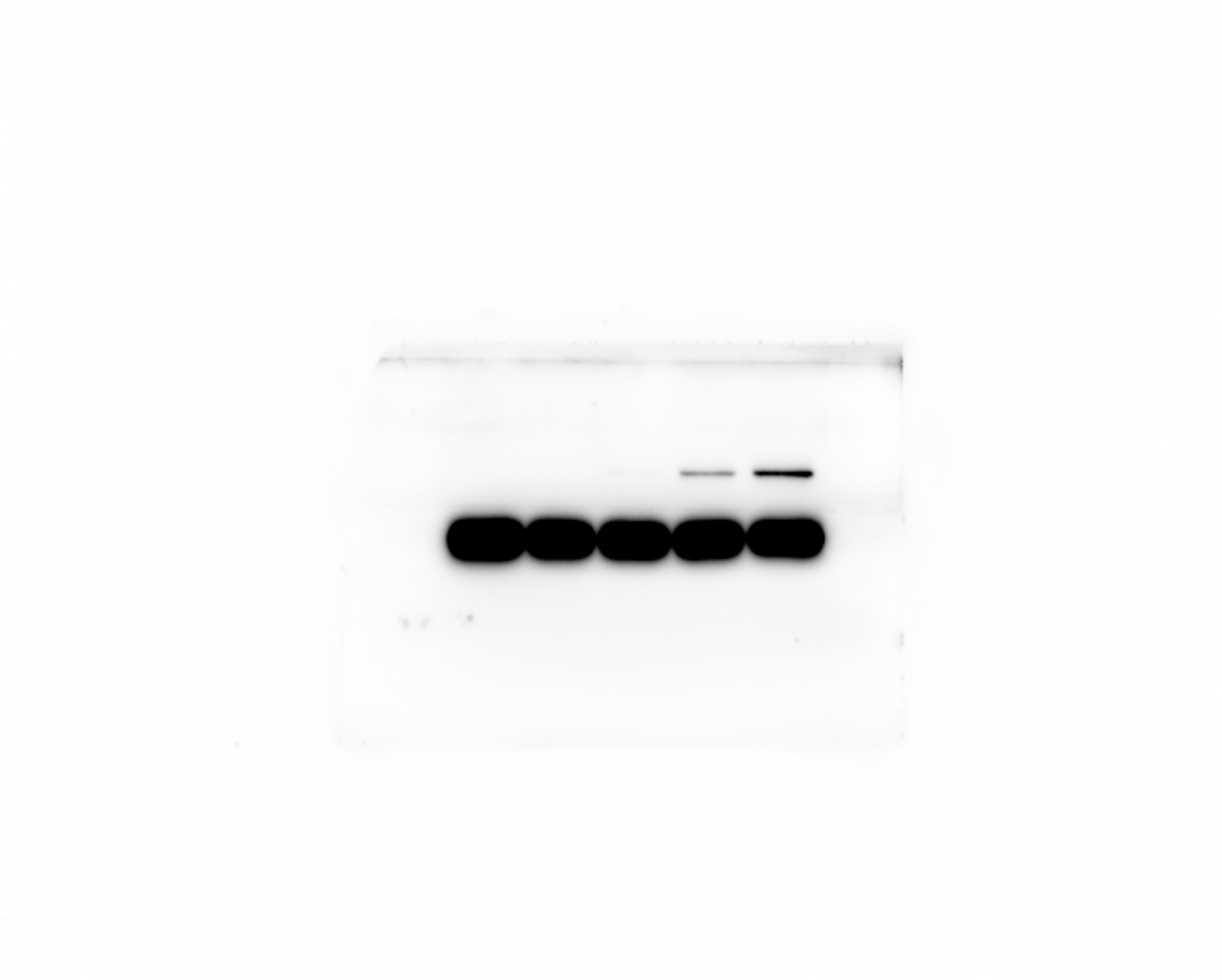

Supplement: FigShare.zip [file KVIR_A_2690825_SM3829.zip › FigShare/Data for figure 2D/ns1'-GAPDH-120S-MERGE.Tif]

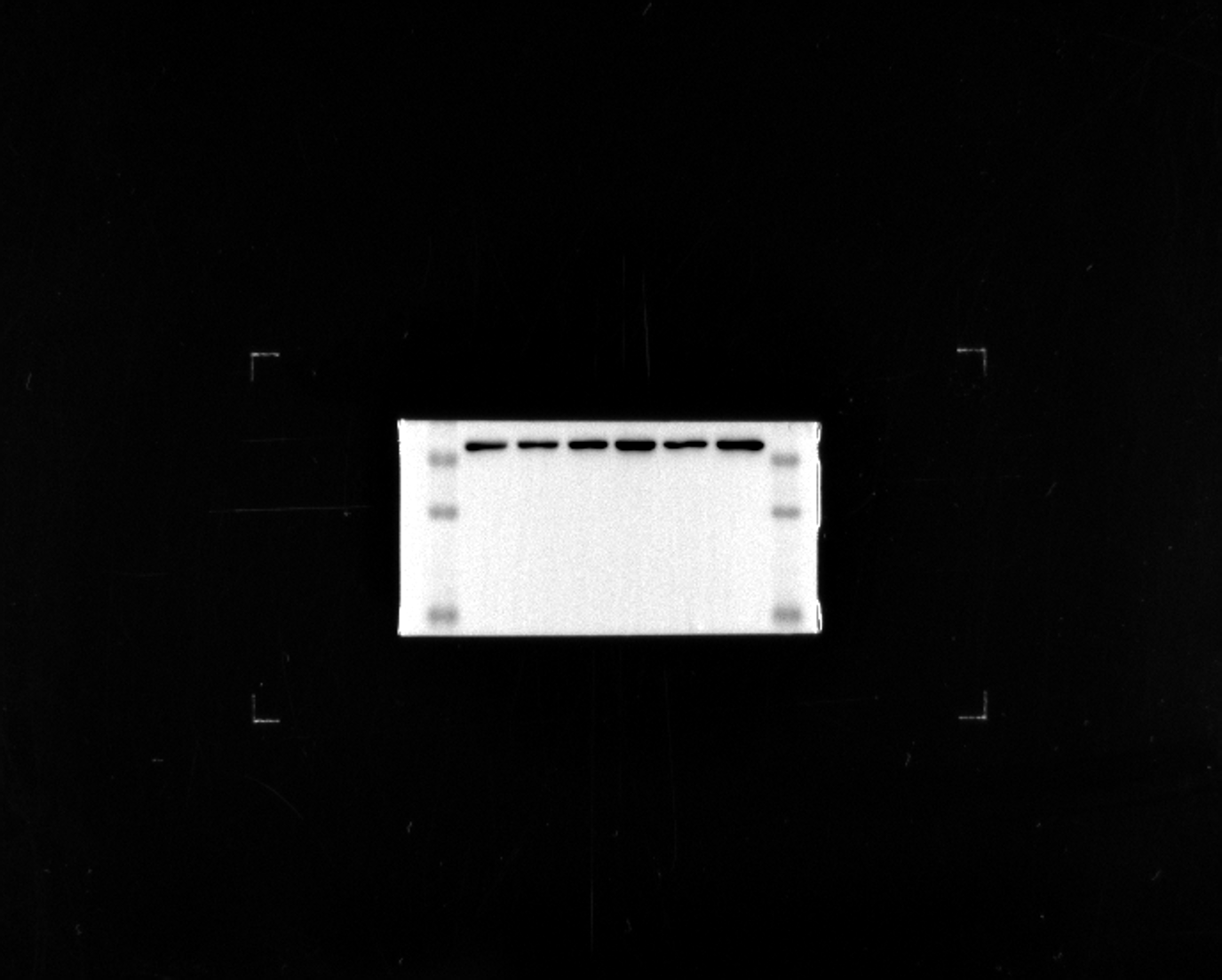

Supplement: FigShare.zip [file KVIR_A_2690825_SM3829.zip › FigShare/Data for figure 5E/149-GAPDH-10s-m.Tif]

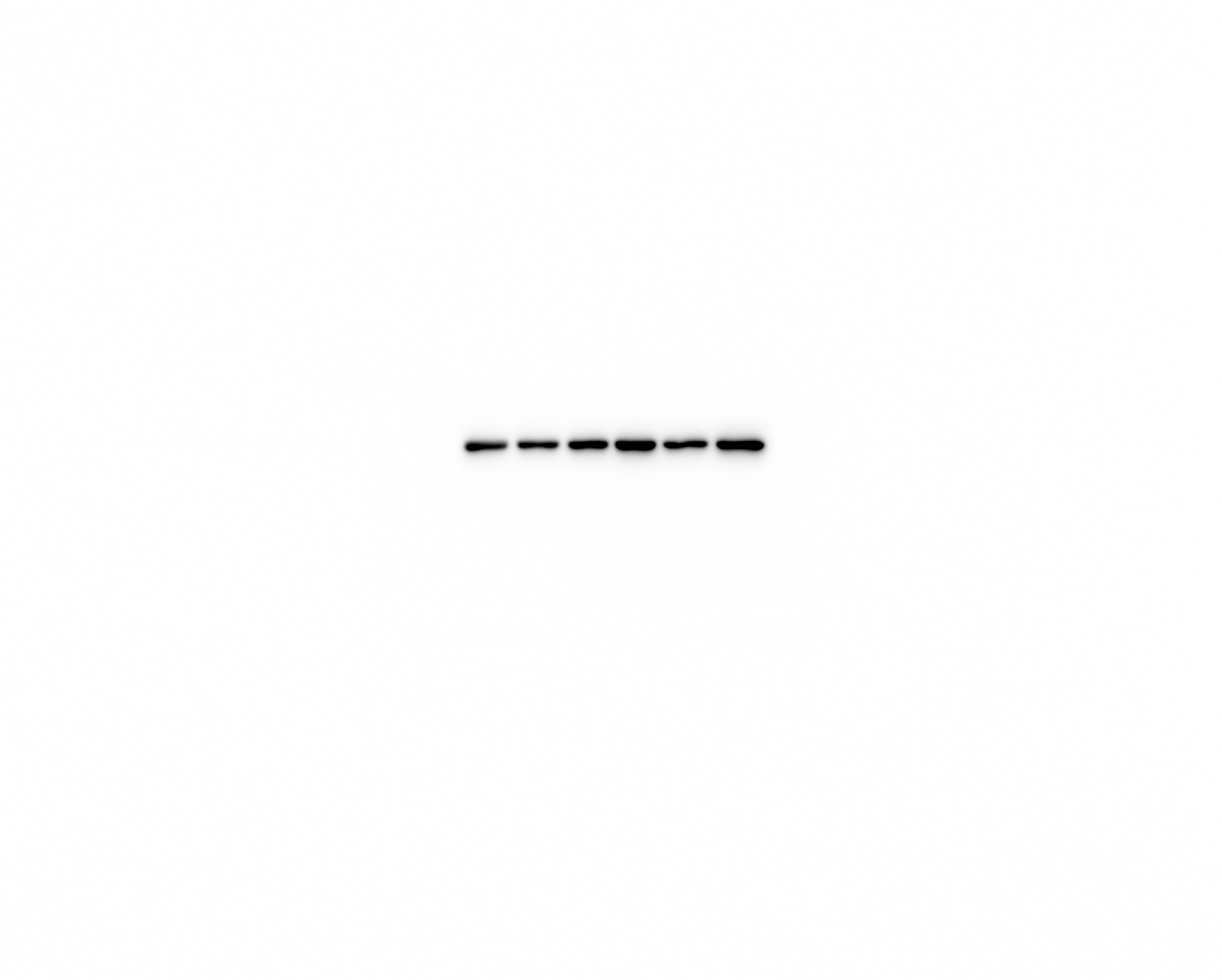

Supplement: FigShare.zip [file KVIR_A_2690825_SM3829.zip › FigShare/Data for figure 5E/149-GAPDH-10s.Tif]

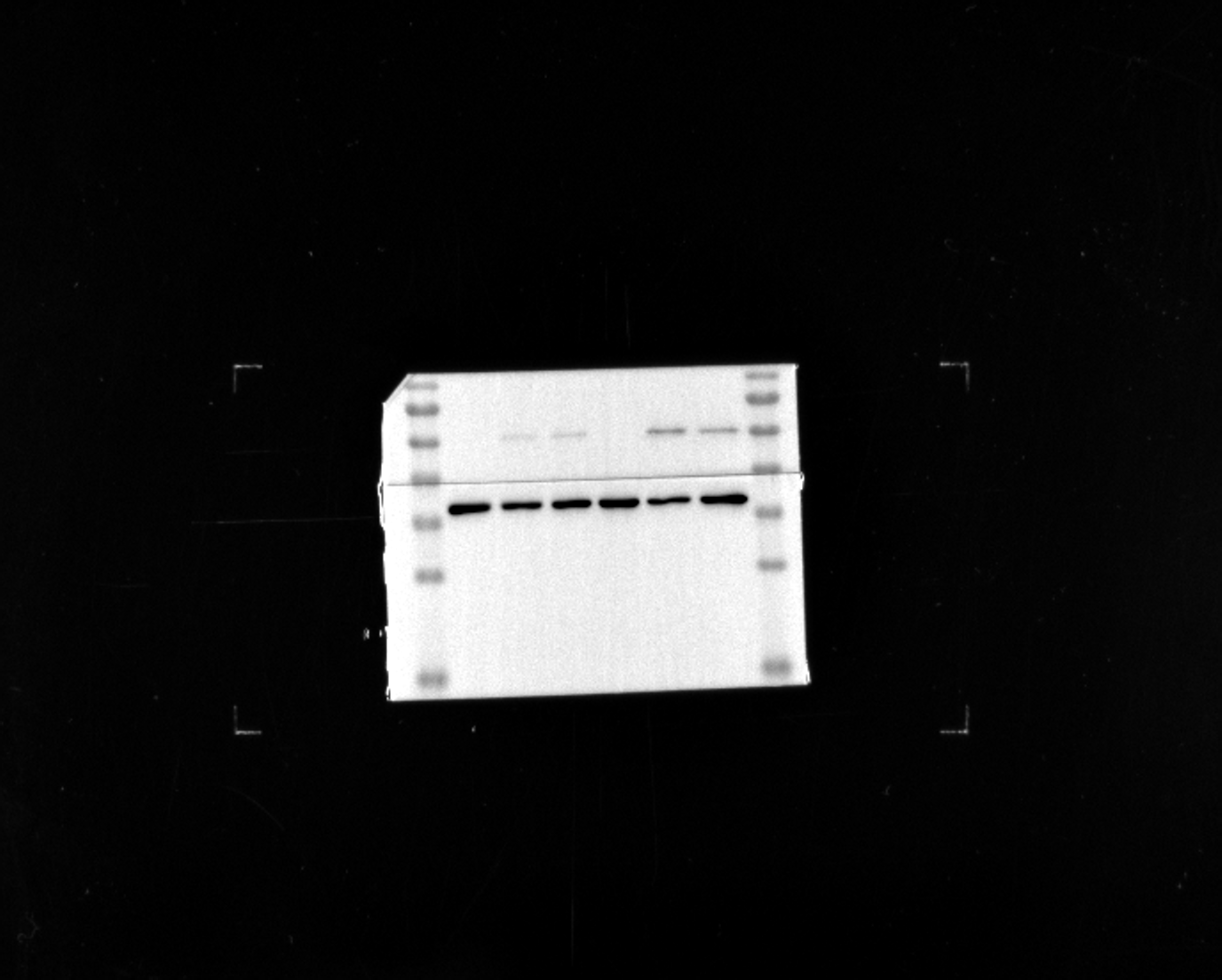

Supplement: FigShare.zip [file KVIR_A_2690825_SM3829.zip › FigShare/Data for figure 5E/149-merge-10s-m.Tif]

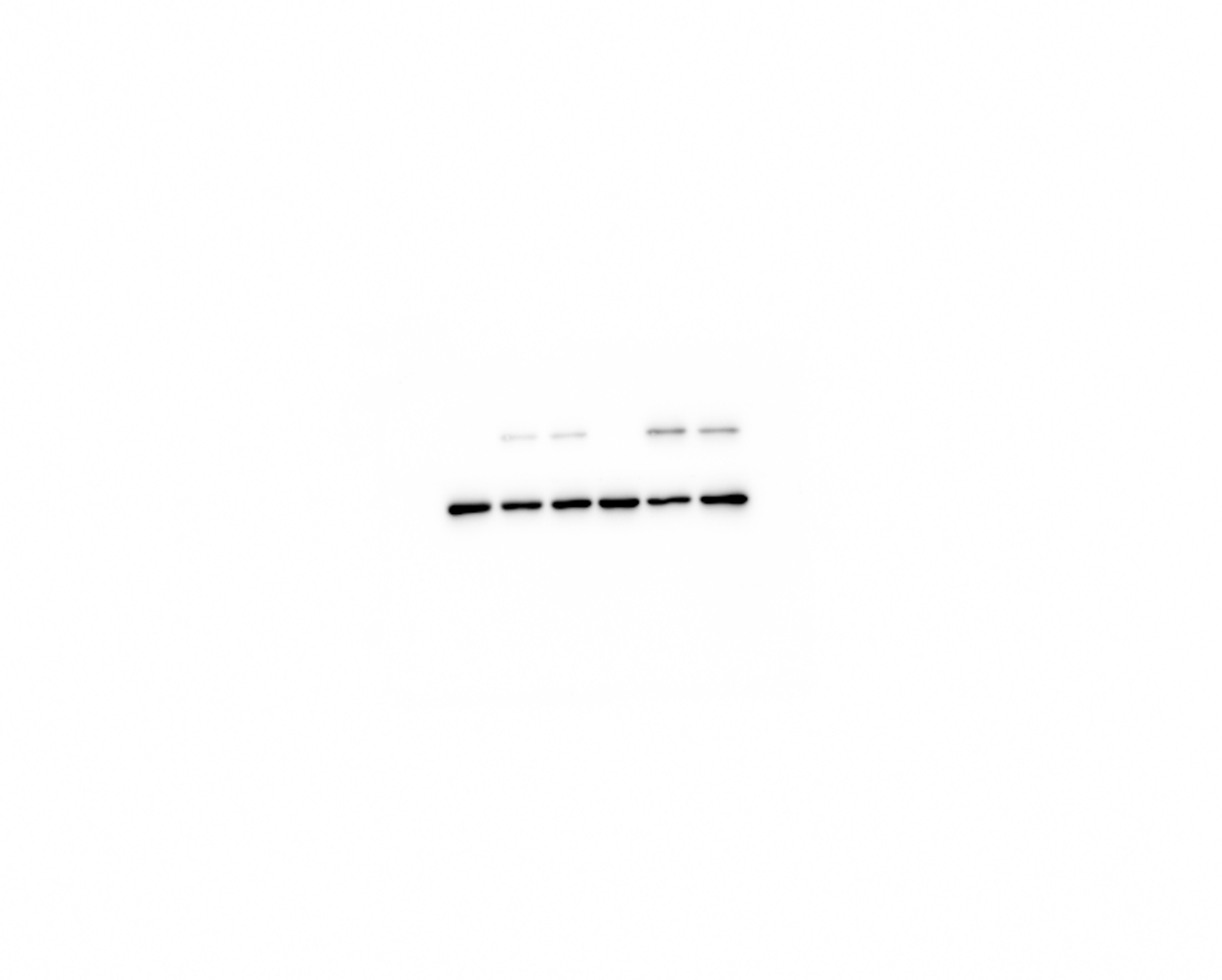

Supplement: FigShare.zip [file KVIR_A_2690825_SM3829.zip › FigShare/Data for figure 5E/149-merge-10s.Tif]

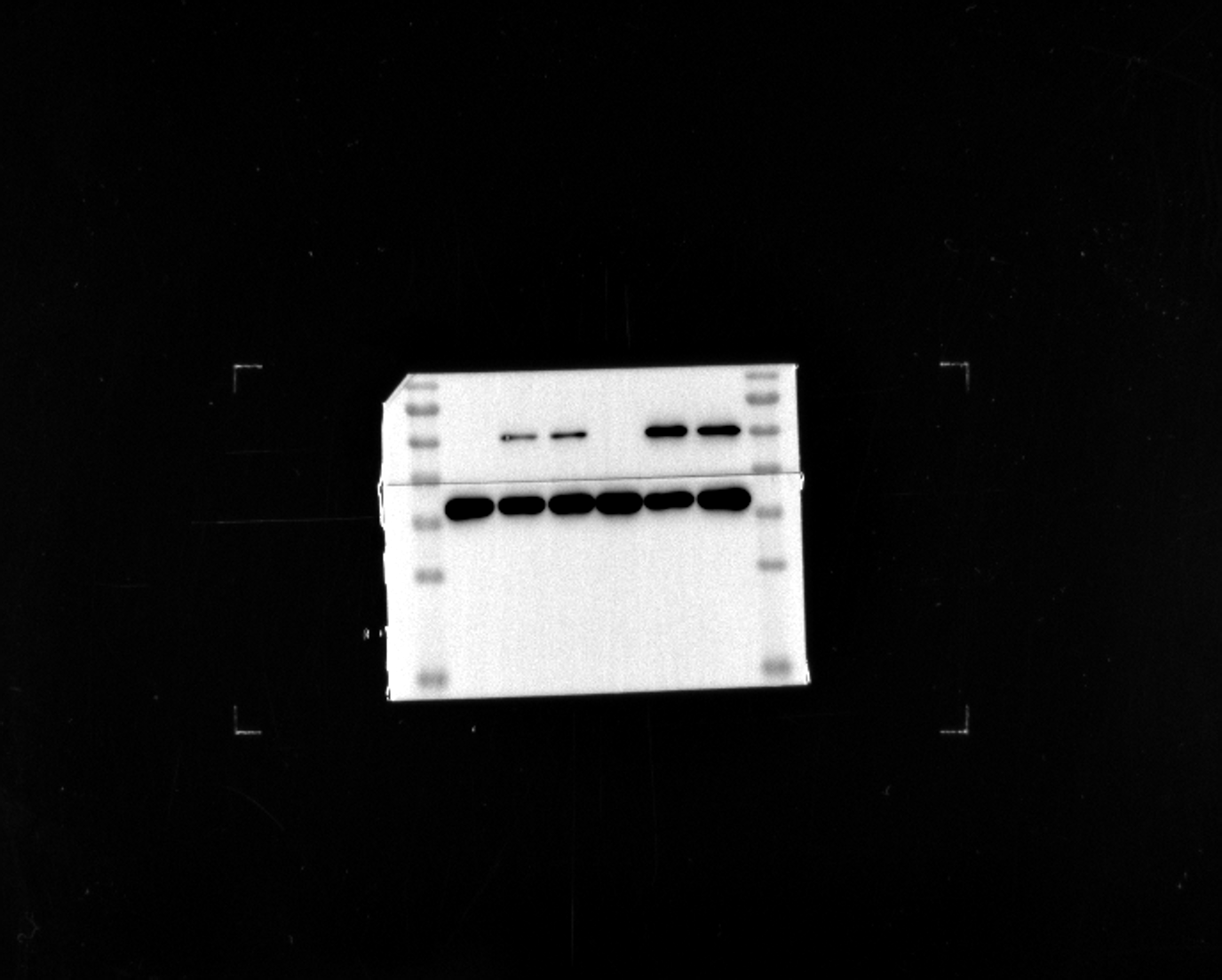

Supplement: FigShare.zip [file KVIR_A_2690825_SM3829.zip › FigShare/Data for figure 5E/149-merge-60s-m.Tif]

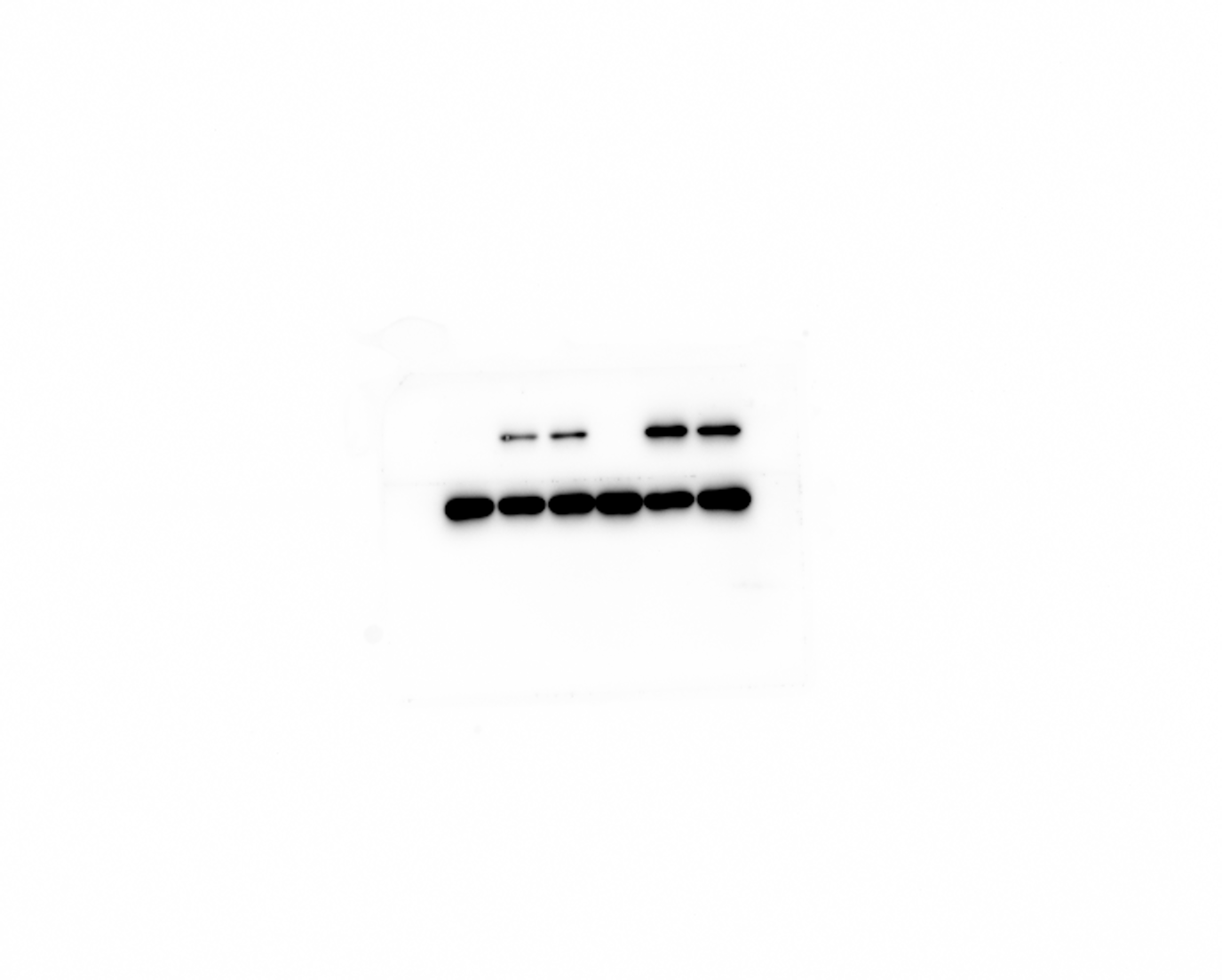

Supplement: FigShare.zip [file KVIR_A_2690825_SM3829.zip › FigShare/Data for figure 5E/149-merge-60s.Tif]

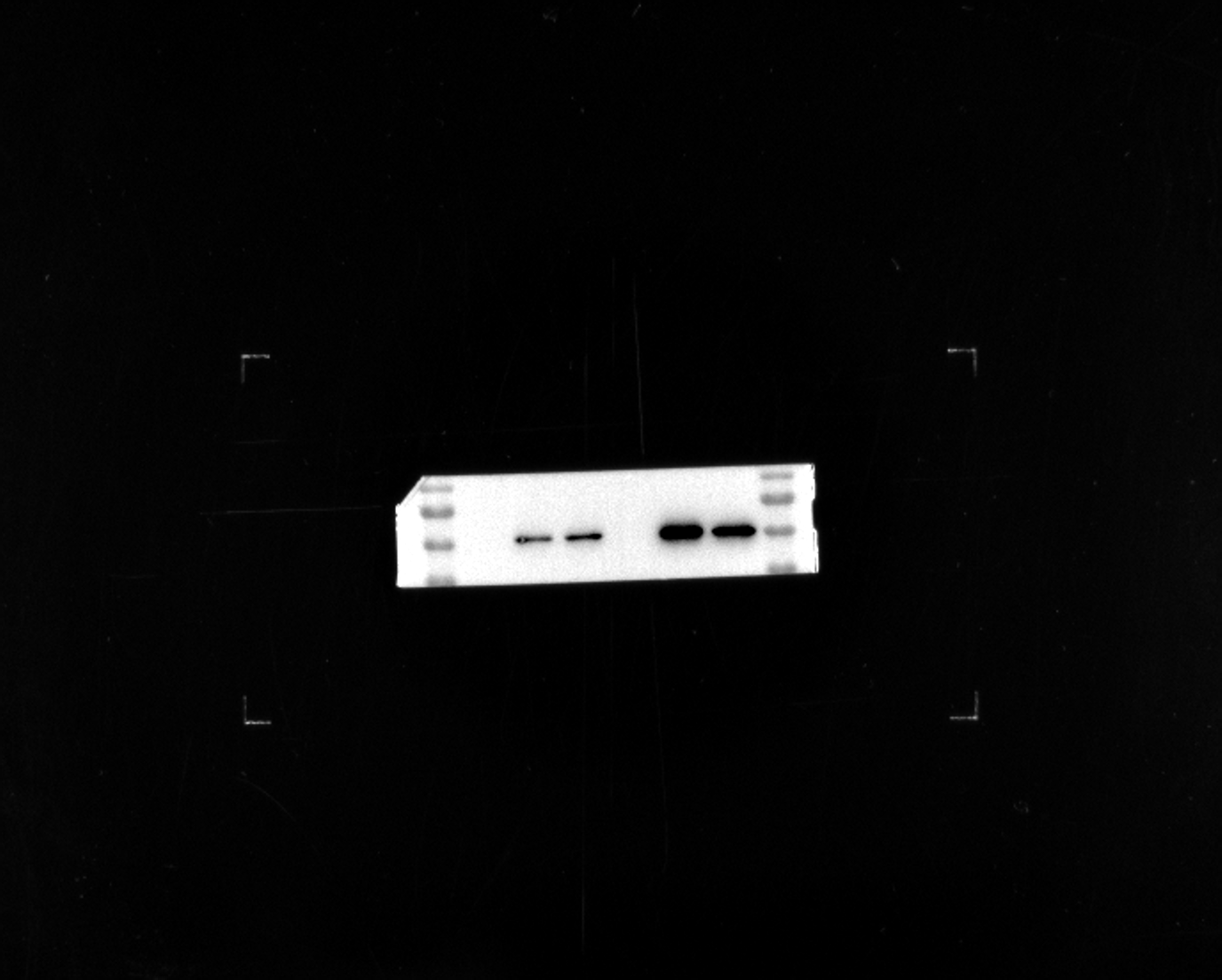

Supplement: FigShare.zip [file KVIR_A_2690825_SM3829.zip › FigShare/Data for figure 5E/149-NS1'-10s-m.Tif]

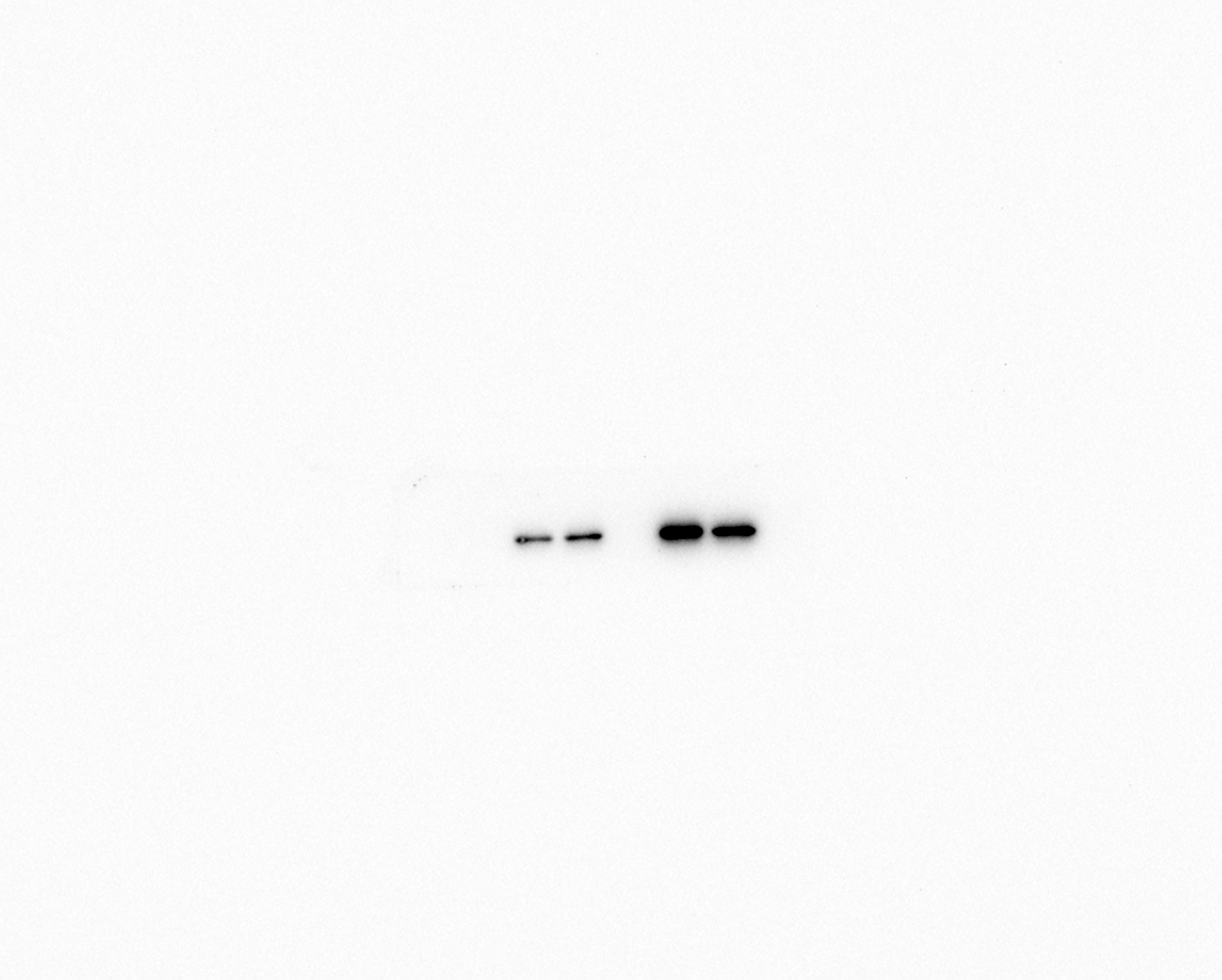

Supplement: FigShare.zip [file KVIR_A_2690825_SM3829.zip › FigShare/Data for figure 5E/149-NS1'-10s.Tif]

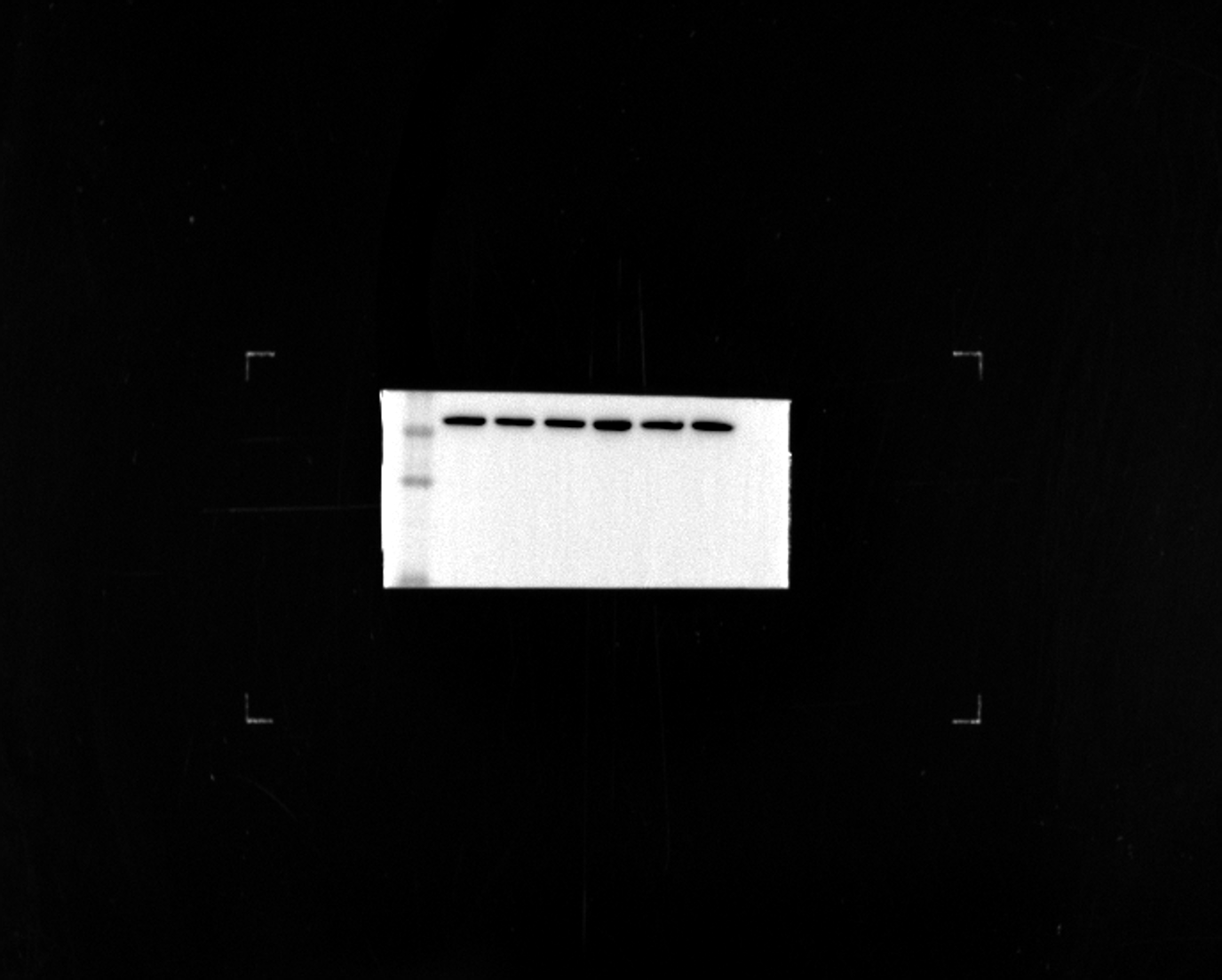

Supplement: FigShare.zip [file KVIR_A_2690825_SM3829.zip › FigShare/Data for figure 5F/483-gapdh-5s-M.Tif]

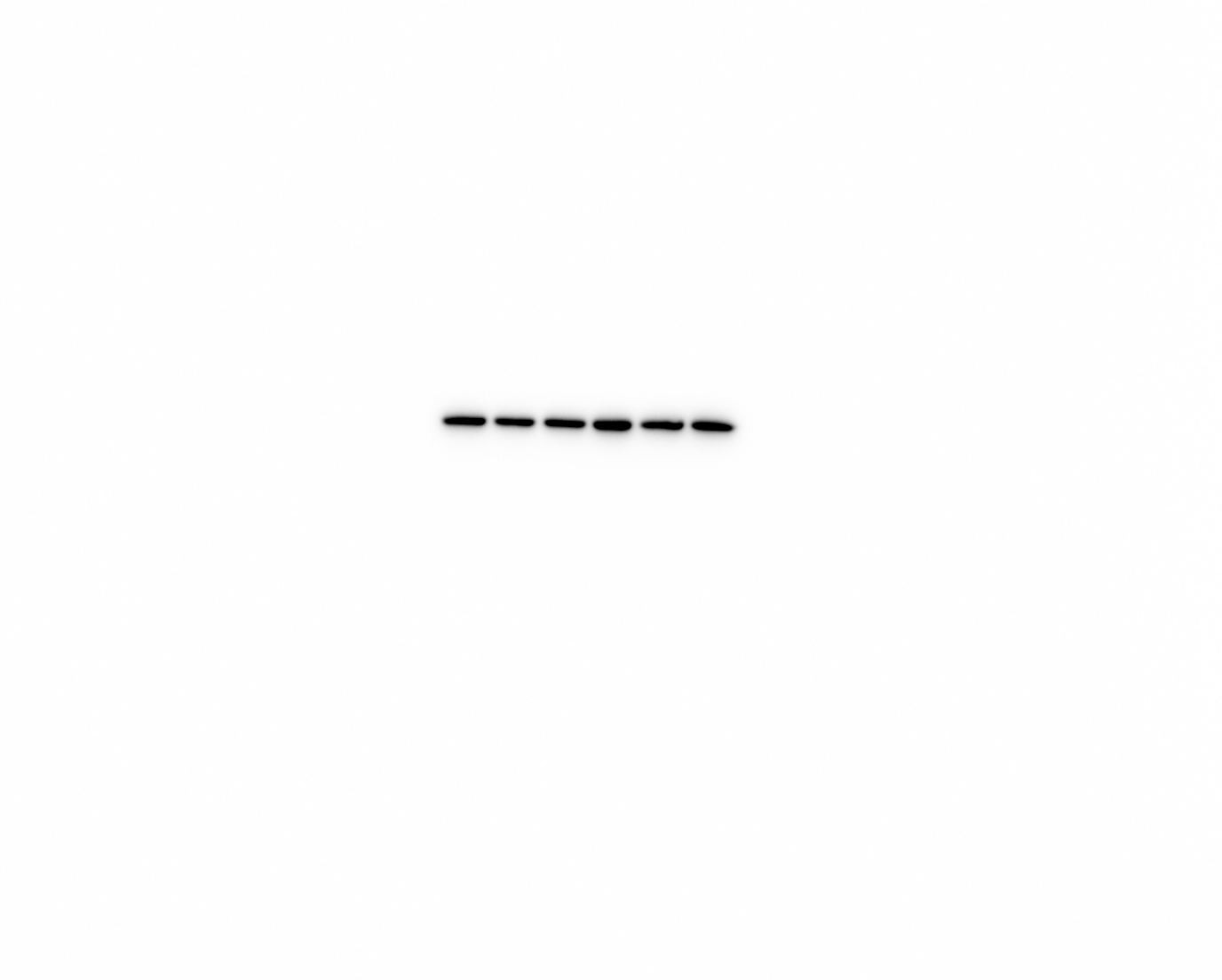

Supplement: FigShare.zip [file KVIR_A_2690825_SM3829.zip › FigShare/Data for figure 5F/483-gapdh-5s.Tif]

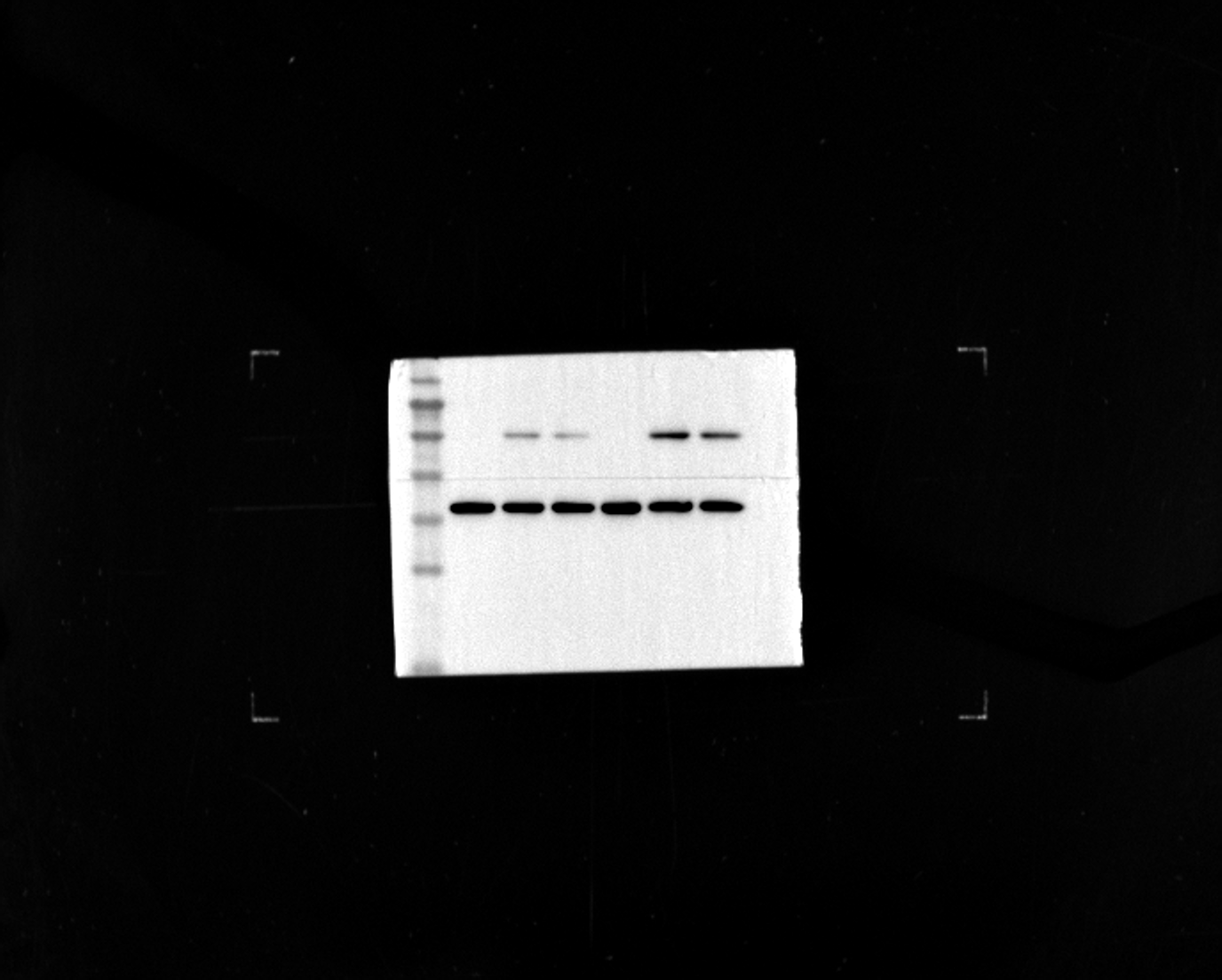

Supplement: FigShare.zip [file KVIR_A_2690825_SM3829.zip › FigShare/Data for figure 5F/483-merge-10s-M.Tif]

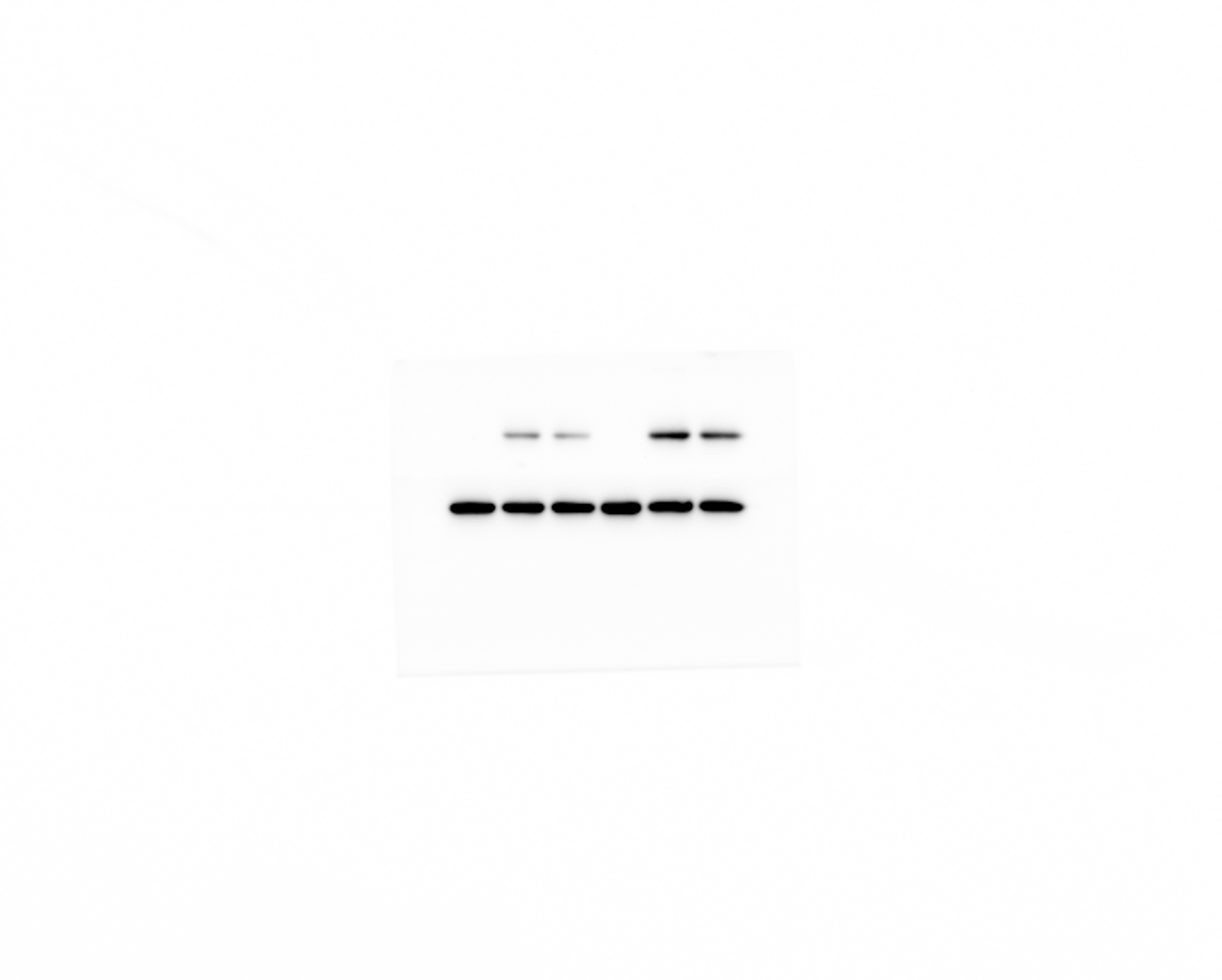

Supplement: FigShare.zip [file KVIR_A_2690825_SM3829.zip › FigShare/Data for figure 5F/483-merge-10s.Tif]

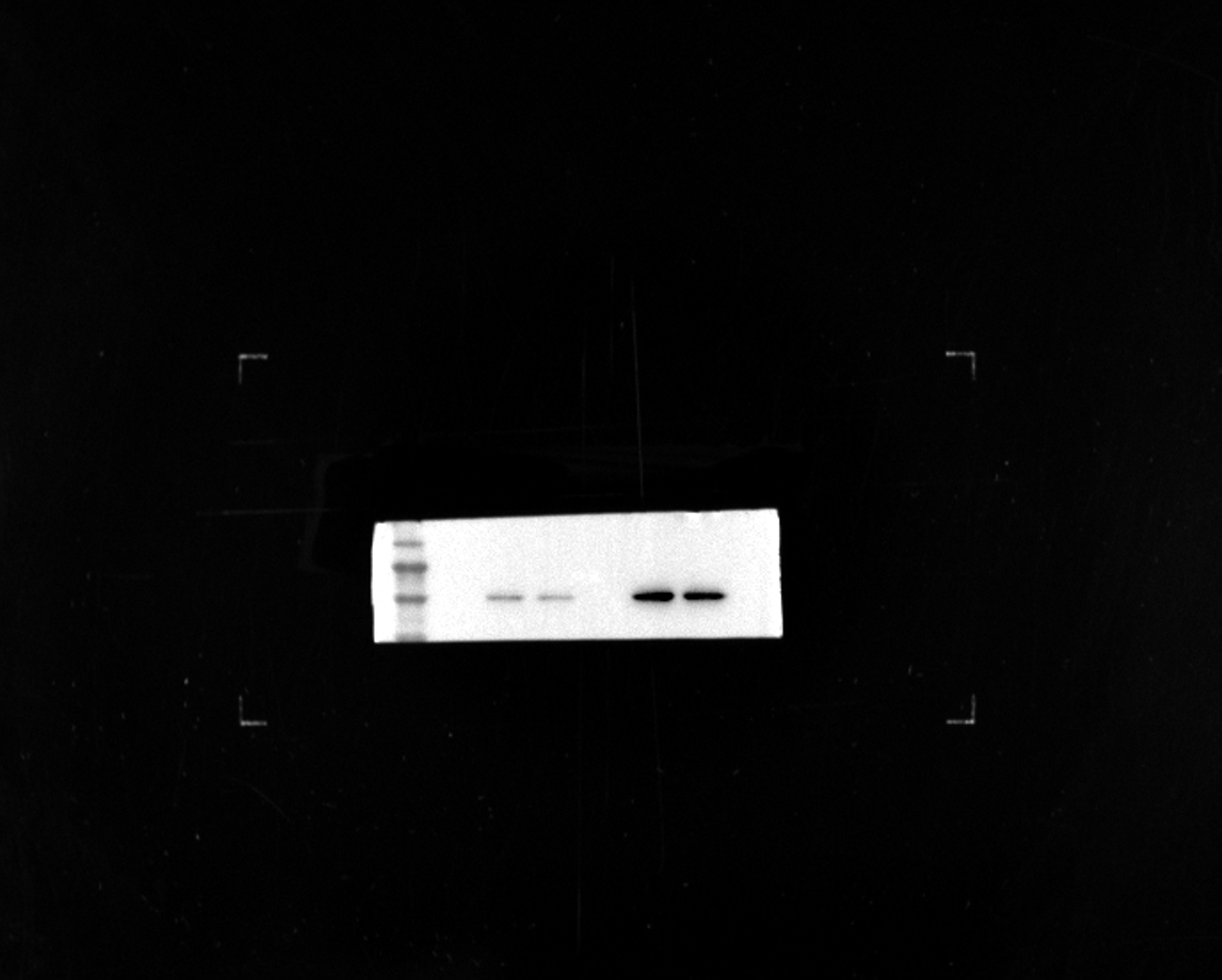

Supplement: FigShare.zip [file KVIR_A_2690825_SM3829.zip › FigShare/Data for figure 5F/483-NS1'-1s-M.Tif]

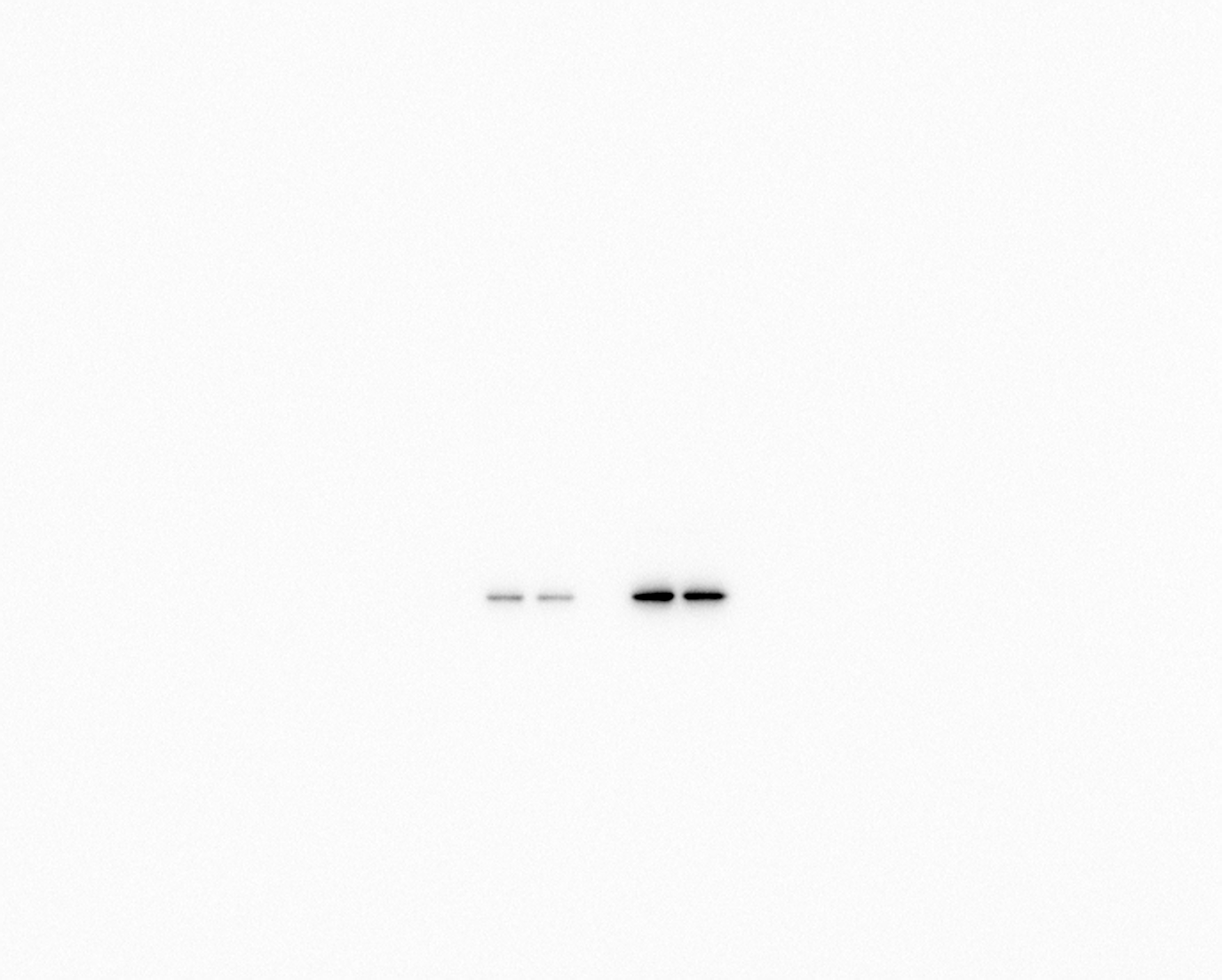

Supplement: FigShare.zip [file KVIR_A_2690825_SM3829.zip › FigShare/Data for figure 5F/483-NS1'-1s.Tif]

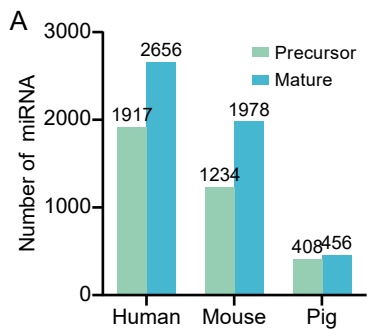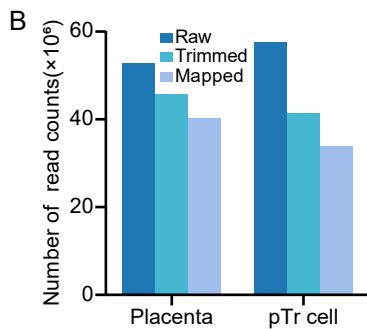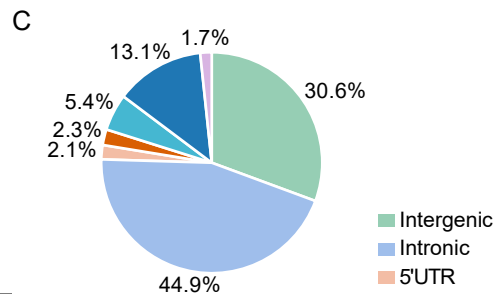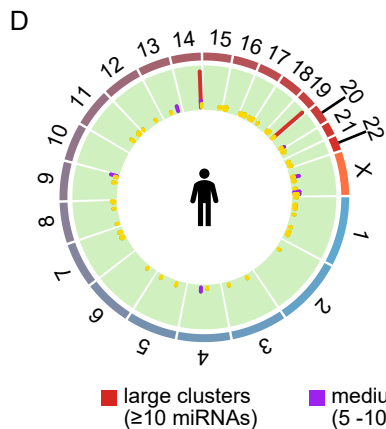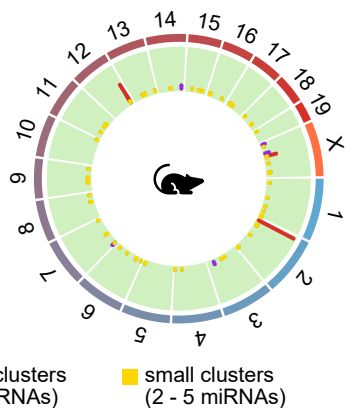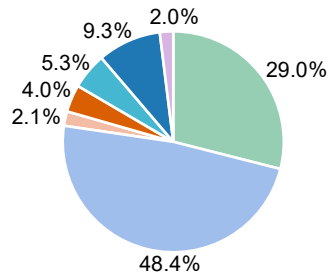

Supplement: FigShare.zip [file KVIR_A_2690825_SM3829.zip › FigShare/Supplementary tables and figures/figure-S1.pdf]

A

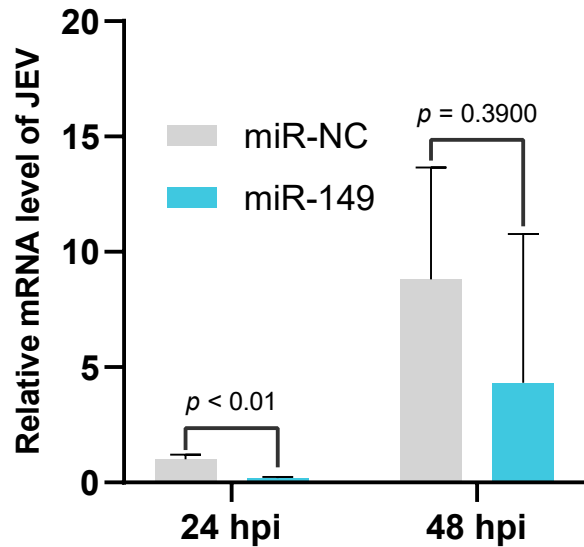

B

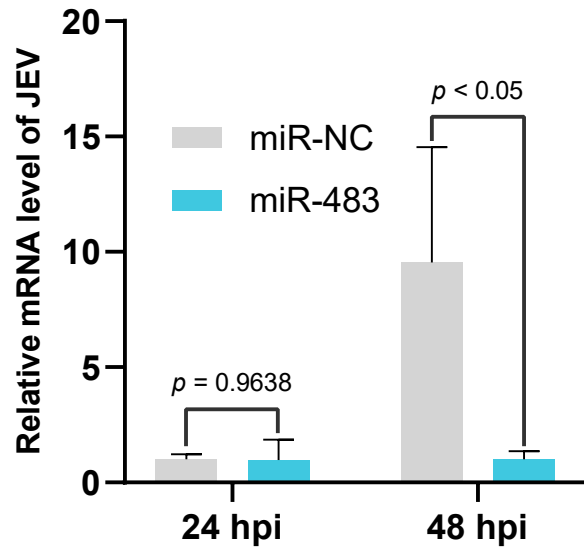

Supplement: FigShare.zip [file KVIR_A_2690825_SM3829.zip › FigShare/Supplementary tables and figures/figure-S4.pdf]
