# Supplementary material for: Integrative transcriptomic analysis uncovers the microRNA-centric regulation of Japanese encephalitis virus infection in porcine trophoblast cells
Source: Virulence. 2026 Jun 17;17(1):2690825. doi: 10.1080/21505594.2026.2690825 (PMC13313263; doi:10.1080/21505594.2026.2690825)
Supplement: Supplemental figures.docx [file KVIR_A_2690825_SM3832.docx]

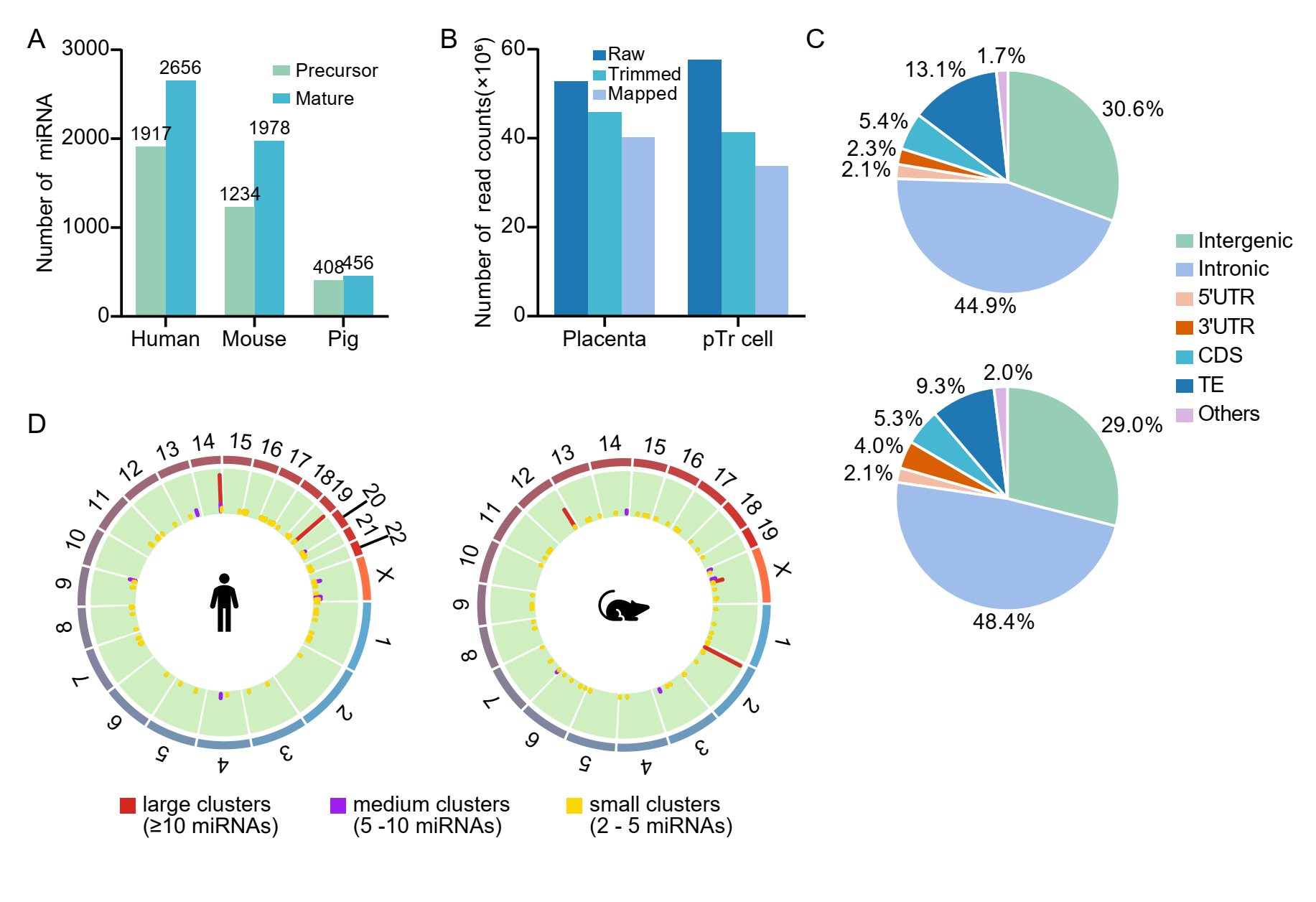


**Figure S1. Comparison of the miRNAs annotated in human, mouse, and pig genomes**

**(A)** The number of miRNAs recorded in the miRBase database for human, mouse and pig. **(B)** Distribution of reads from small RNA-seq data derived from porcine placenta and pTr cells. "Raw" refers to all reads obtained directly from sequencing; "Trimmed" indicates reads following adapter removal and quality control; "Mapped" represents reads successfully mapped to the reference porcine genome. **(C)** The distribution of human (upper panel) and mouse (lower panel) precursor miRNA genes across various genomic elements. The overlapping region of the precursor miRNA gene with a certain genomic element accounts for at least 80% of its length. TE: transposable element. **(D)** Circos diagram illustrating the sizes of human (left panel) and mouse (right panel) miRNA clusters and their distributions across chromosomes. The outermost ring displays individual chromosomes, and the inner ring represents distinct miRNA clusters. The length and color of the lines indicate the size of each cluster, defined as the number of miRNAs it contains.


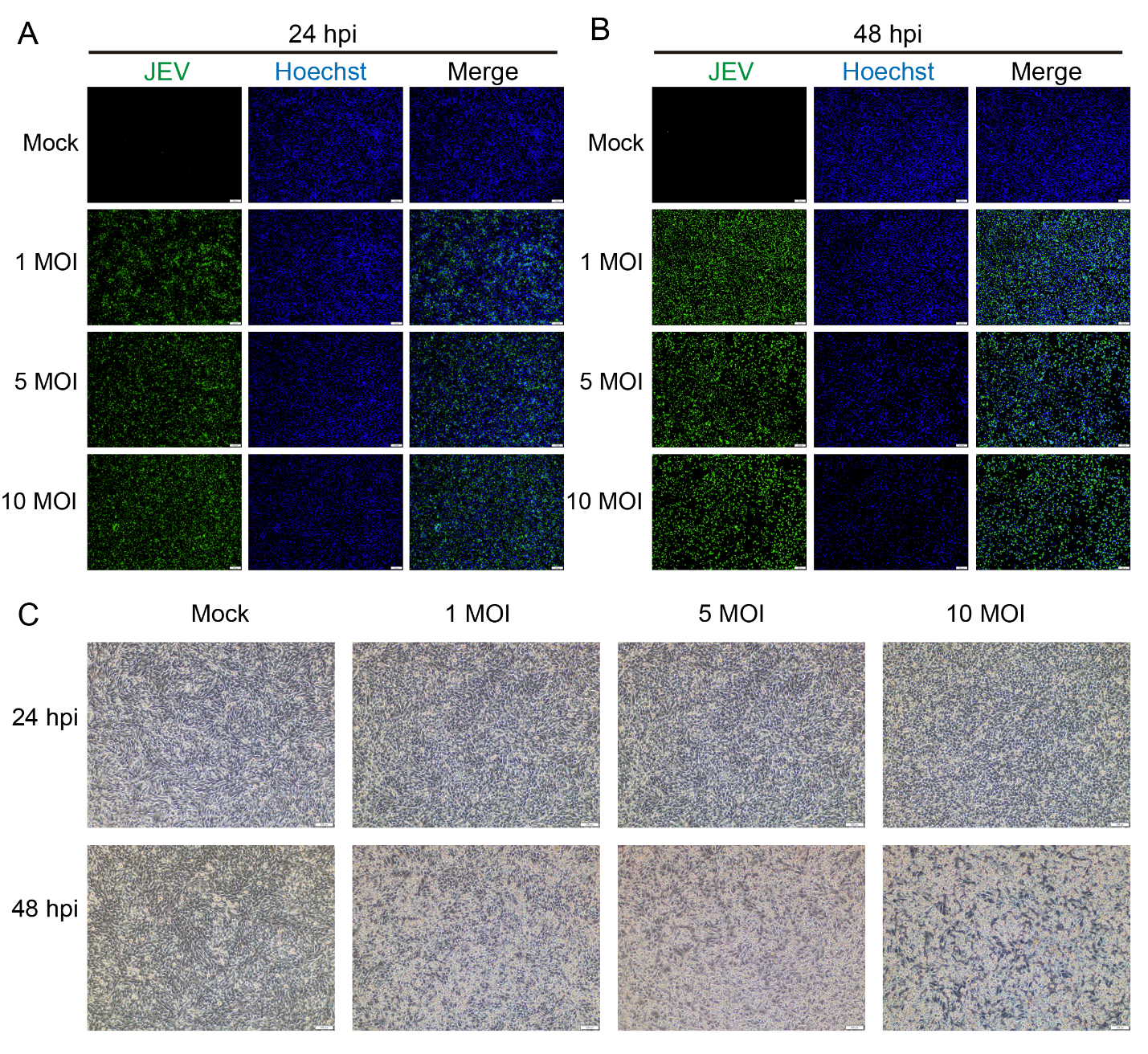


**Figure S2. JEV infection in BHK-21 cells with different doses and durations**

**(A,B)** Indirect immunofluorescence imaging to visualize JEV infection of BHK-21 cells with different doses at 24 and 48 hpi, respectively. Mouse-derived anti-JEV serum was used for this experiment. **(C)** Cytopathic effects of BHK-21 cells following JEV infection with different doses at 24 and 48 hpi, respectively.


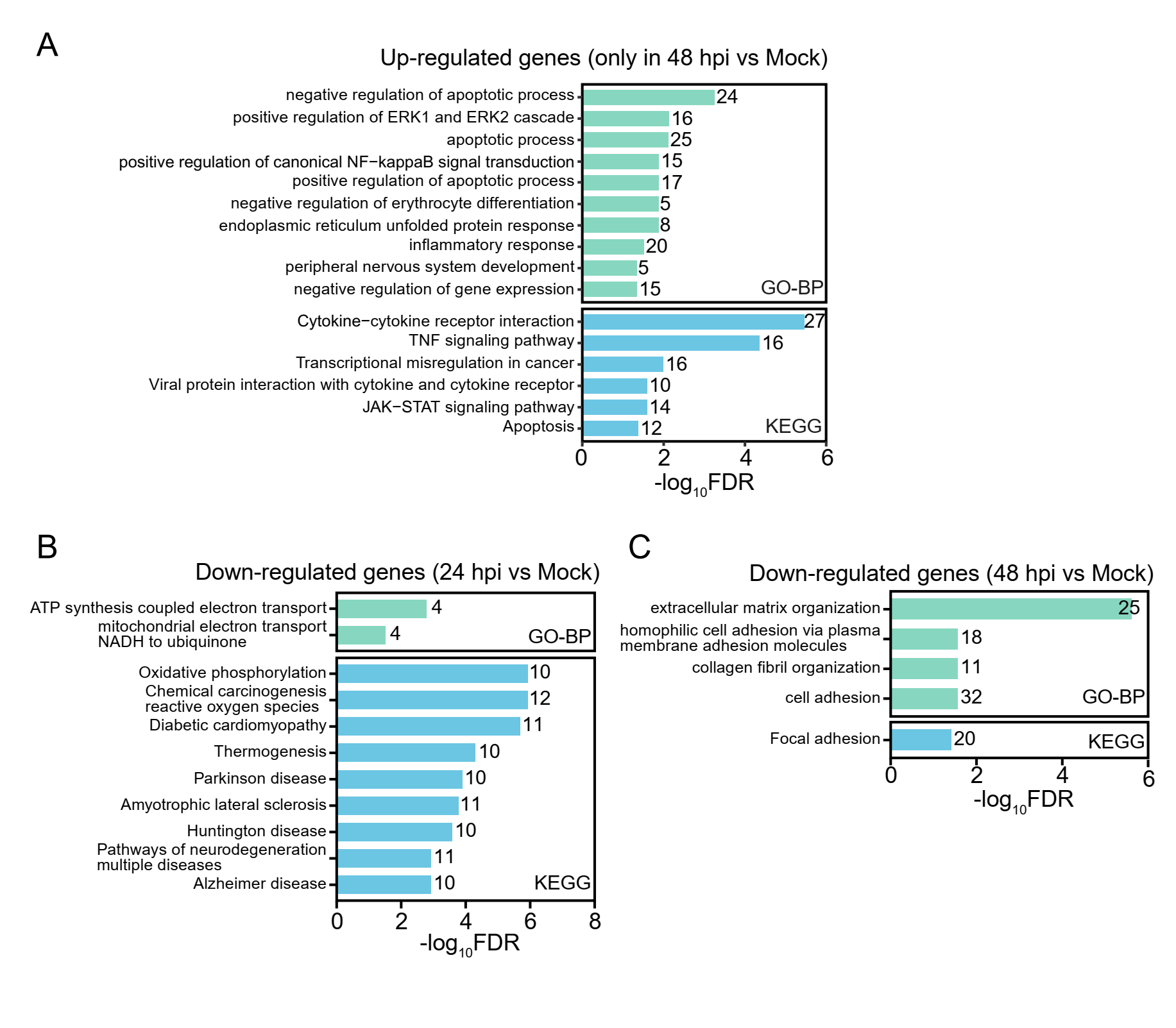


**Figure S3. Functional enrichment analysis of the JEV-affected genes in pTr cells**

**(A)** Enriched GO and KEGG terms for genes that were only up-regulated (FDR < 0.05 and log2foldchange > 1) at 48 hpi but not at 24 hpi compared to the Mock-infected group. **(B,C)** Enriched GO and KEGG terms for genes exhibiting down-regulated expression in the 24 hpi **(B)** and 48 hpi **(C)** compared to the Mock-infected group. Only top ten terms from “Biological Process” category and KEGG pathway are shown. The numeric value displayed at the terminus of each bar represents the count of genes enriched for the corresponding functional term or pathway.

**
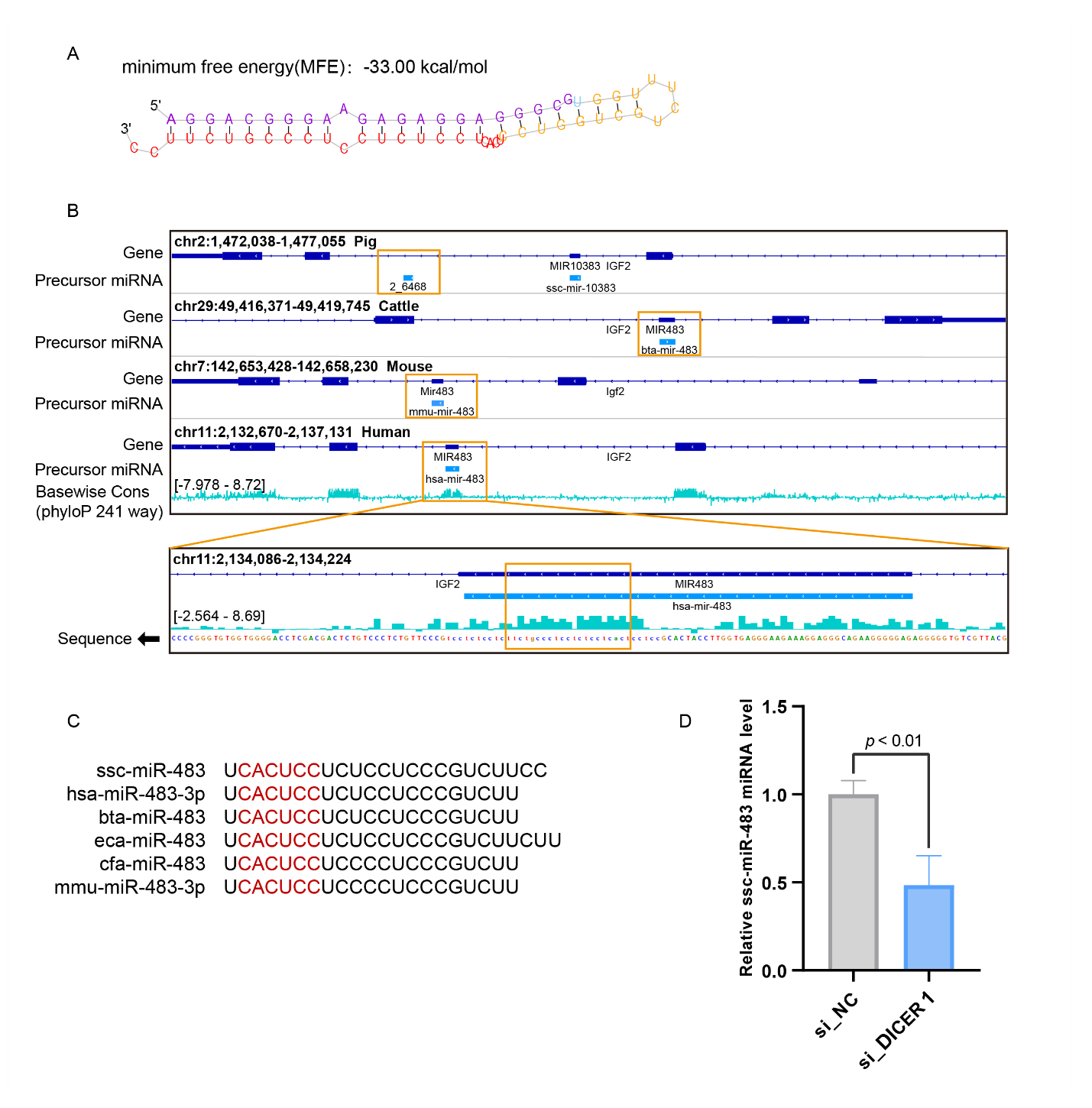
**

**Figure S4. Validation of ssc-miR-483 as a *bona fide* porcine miRNA**

**(A)** The secondary structure and stability of the precursor miRNA of ssc-miR-483. The minimum free energy (MFE) were computed using RNAfold. **(B)** The IGV tracks show the location of the precursor miRNA genes of miR-483 in the genomes of pig, cattle, mouse, and human, as well as its conservation in placental mammals. Here, the precursor miRNA gene 2_6468 of miR-483 in pig was generated by miRDeep2 as a provisional id. **(C)** Sequence alignments of miR-483(-3p) in different species. **(D)** RT-qPCR analysis for ssc-miR-483 expression (normalized to *U6*) in pTr cells after transfection with siRNA of DICER1 for 36 hours. Data are mean ± SD, n =3. Two-tailed *P* values were analyzed using Student's t-test.

**
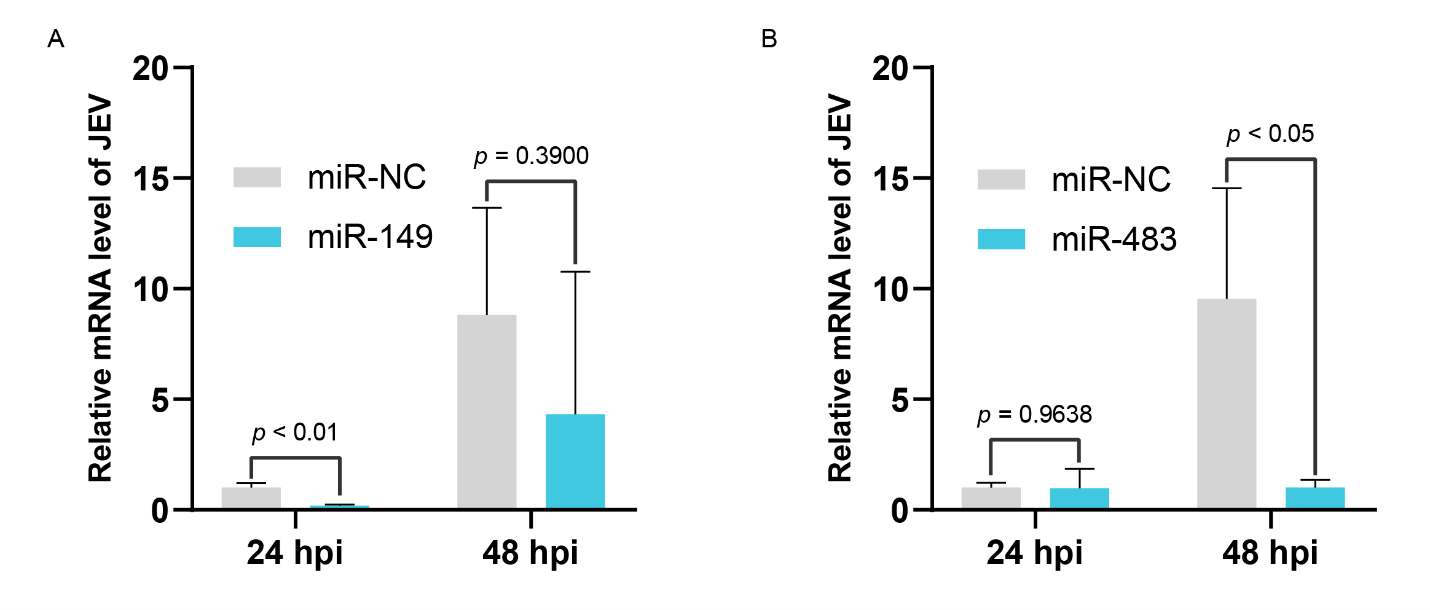
**

**Figure S5. Evaluation of JEV RNA levels by RT-qPCR**

PTr cells were transfected with ssc-miR-149 **(A)** and ssc-miR-483 **(B)** mimics (miR-149 and miR-483) and negative control (miR-NC) for 24 h and then infected with JEV at a MOI of 5. Cells were harvested at 24 and 48 hpi, respectively. Data are mean ± SD, n =3. Two-tailed *P* values were analyzed using Student's t-test.
