# Supplementary material for: Integrative transcriptomic analysis uncovers the microRNA-centric regulation of Japanese encephalitis virus infection in porcine trophoblast cells
Source: Virulence. 2026 Jun 17;17(1):2690825. doi: 10.1080/21505594.2026.2690825 (PMC13313263; doi:10.1080/21505594.2026.2690825)
Supplement: FigShare.zip [file KVIR_A_2690825_SM3829.zip › FigShare/Data for figure S3A.pdf]

Provisional ID	:	2_6468
Score total	:	44043.1
Score for star read(s)	:	3.9
Score for read counts	:	44035.3
Score for mfe	:	2.4
Score for randfold	:	1.6
Score for cons. seed	:	
Total read count	:	86385
Mature read count	:	79192
Loop read count	:	2
Star read count	:	7191

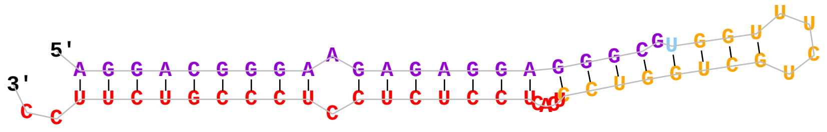

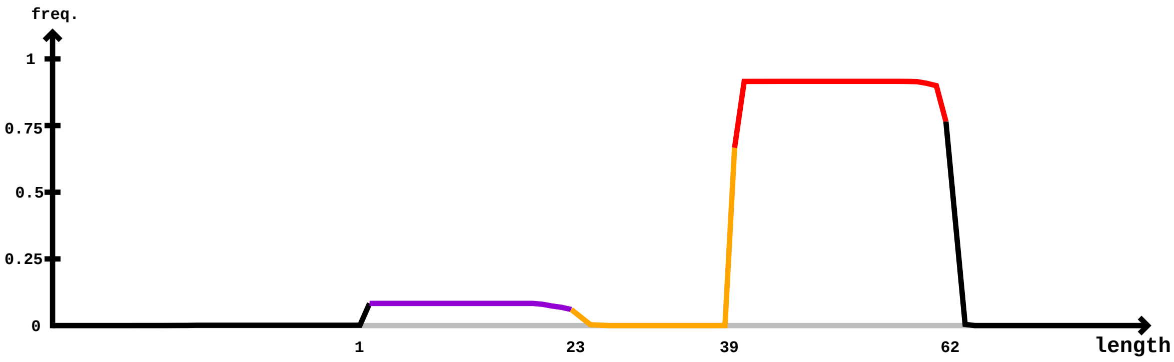

Star Mature

```
5'- agccgagucggagcugucggaggcgagggcgaggacgggaagagaggagggcgugguuucugcugguccucacuccucuccucccgucuuccuccuccuccucccauuccca -3'  obs
    agccgagucggagcugucggaggcgagggcgaggacgggaagagaggagggcgugguuucugcugguccucacuccucuccucccgucuuccuccuccuccucccauuccca       exp
    .........((((.((..(((((.(((((.((((((((((.(((((((((((.((((....))))))))....))))))).)))))))))).)))))..)))))))))))..       reads  mm  sample
    .......ucggagcugucggaggcgagggcg.................................................................................       7      0   pl1
    ........cggagcugucggaggcgagggcg.................................................................................       1      0   pl1
    .........ggagcugucggaggcgagggcg.................................................................................       1      0   pl1
    ..........gagcugucggaggcgagggcg.................................................................................       4      0   pl1
    ...........agcugucggaggcgagggcg.................................................................................       10     0   pl1
    ............gcugucggaggcgagggcg.................................................................................       8      0   pl1
    .............cugucggaggcgagggcg.................................................................................       16     0   pl1
    ..............................Uaggacgggaagagaggagggc............................................................       3      1   pl1
    ..............................Uaggacgggaagagaggagggcg...........................................................       2      1   pl1
    ...............................aggacgggaagGgaggag...............................................................       1      1   pl1
    ...............................aggacAggaagagaggag...............................................................       2      1   pl1
    ...............................aggacgggaagUgaggag...............................................................       1      1   pl1
    ...............................Uggacgggaagagaggag...............................................................       1      1   pl1
    ...............................aggacgggaagagUggag...............................................................       1      1   pl1
    ...............................aggacgggCagagaggag...............................................................       2      1   pl1
    ...............................aggacCggaagagaggag...............................................................       1      1   pl1
    ...............................aggacggCaagagaggag...............................................................       1      1   pl1
    ...............................aggacgggaagagagAag...............................................................       3      1   pl1
    ...............................aggacgggaagagaggag...............................................................       98     0   pl1
    ...............................aggacgggaagagaggNg...............................................................       1      1   pl1
    ...............................aggacgCgaagagaggag...............................................................       2      1   pl1
    ...............................aggacgggaagUgaggagg..............................................................       3      1   pl1
    ...............................aggacgggaagagaggaAg..............................................................       2      1   pl1
    ...............................aggacgggaagagaggagA..............................................................       9      1   pl1
    ...............................aggacgggaagagaggagC..............................................................       1      1   pl1
    ...............................aggacgggaagagaggagU..............................................................       14     1   pl1
    ...............................aggacgggaagGgaggagg..............................................................       2      1   pl1
    ...............................agUacgggaagagaggagg..............................................................       1      1   pl1
    ...............................aAgacgggaagagaggagg..............................................................       1      1   pl1
    ...............................aggacgggCagagaggagg..............................................................       1      1   pl1
    ...............................aggacggCaagagaggagg..............................................................       1      1   pl1
    ...............................Nggacgggaagagaggagg..............................................................       1      1   pl1
    ...............................aggacgggaagagaggNgg..............................................................       1      1   pl1
```

Star / Mature			
agccgagucggagcugucggaggcgagggcgaggacgggaagagaggagggcgugguuucugcugguccucacuccucuccucccgucuuccuccuccuccucccauuccca			
...............................Cggacgggaagagaggagg..	1	1	pl1
...............................aggacgggaagagaCgagg..	1	1	pl1
...............................aggacgggaagagaggagg..	215	0	pl1
...............................aggacggCaagagaggaggg...	1	1	pl1
...............................aggacAggaagagaggaggg...	1	1	pl1
...............................aggacgggaagagaggaggg...	173	0	pl1
...............................aggacgggaagagaggNggg...	2	1	pl1
...............................aggacgNgaagagaggaggg...	1	1	pl1
...............................aggacgggaagagaCgaggg...	1	1	pl1
...............................aggacgCgaagagaggaggg...	2	1	pl1
...............................aggacgggaaCagaggaggg...	1	1	pl1
...............................aggacggAaagagaggaggg...	1	1	pl1
...............................aggacCggaagagaggaggg...	1	1	pl1
...............................aggacgggaagagaggUggg...	1	1	pl1
...............................aggacgggaagagaggaggA...	2	1	pl1
...............................agCacgggaagagaggaggg...	1	1	pl1
...............................aggacgggaagagaggaggU...	19	1	pl1
...............................aggacggAaagagaggagggc..	1	1	pl1
...............................aggacgggaagagaggagggU..	22	1	pl1
...............................aggCcgggaagagaggagggc..	1	1	pl1
...............................aggacgggaagagaggaggCc..	3	1	pl1
...............................aggacAggaagagaggagggc..	1	1	pl1
...............................aggacgCgaagagaggagggc..	2	1	pl1
...............................aggacgggaagagaggNgggc..	1	1	pl1
...............................aggacgggaagagaggaCggc..	1	1	pl1
...............................aggacgggaagagaggagggG..	2	1	pl1
...............................aggacgggaagUgaggagggc..	1	1	pl1
...............................aggacgggaUgagaggagggc..	2	1	pl1
...............................agCacgggaagagaggagggc..	1	1	pl1
...............................aggacgggaagagagCagggc..	2	1	pl1
...............................aggacgAgaagagaggagggc..	2	1	pl1
...............................aggacgggUagagaggagggc..	1	1	pl1
...............................aggacgggaagagaggagggc..	274	0	pl1
...............................Nggacgggaagagaggagggc..	2	1	pl1
...............................aggacgggaagagaggNgggcg...	9	1	pl1
...............................aggacggCaagagaggagggcg...	4	1	pl1
...............................aggacgAgaagagaggagggcg...	2	1	pl1
...............................aggacgNgaagagaggagggcg...	8	1	pl1
...............................aggacgggaagagaggagggGg...	1	1	pl1
...............................aggacgggaaCagaggagggcg...	2	1	pl1
...............................aggacgggCagagaggagggcg...	3	1	pl1
...............................aggUcgggaagagaggagggcg...	1	1	pl1
...............................aggacgggaagagaggaggAcg...	9	1	pl1
...............................agCacgggaagagaggagggcg...	7	1	pl1
...............................aggacUggaagagaggagggcg...	1	1	pl1
...............................aggacgggUagagaggagggcg...	2	1	pl1
...............................aggacgggaagagaggaggCcg...	6	1	pl1
...............................aggacgggaagagaAgagggcg...	1	1	pl1
...............................agAacgggaagagaggagggcg...	1	1	pl1
...............................aUgacgggaagagaggagggcg...	1	1	pl1
...............................aggacgCgaagagaggagggcg...	7	1	pl1
...............................Nggacgggaagagaggagggcg...	4	1	pl1
...............................aggacgggaagagaggagggcg...	1825	0	pl1
...............................aggacgggaUgagaggagggcg...	1	1	pl1
...............................aggacgggaagagaggUgggcg...	5	1	pl1
...............................aggacgggaagagaggagggcA...	16	1	pl1
...............................aggacgggaagagagAagggcg...	1	1	pl1
...............................aggacgggGagagaggagggcg...	4	1	pl1
...............................aggacgggaagagaggagCgcg...	2	1	pl1
...............................aggacgggaagagaggagggUg...	2	1	pl1
...............................aggacgggaagUgaggagggcg...	3	1	pl1
...............................Gggacgggaagagaggagggcg...	1	1	pl1
...............................aggacgggaagagaggagAgcg...	5	1	pl1
...............................aggacgggaagagaggagUgcg...	1	1	pl1
...............................aggacgggaagagaggagggcU...	18	1	pl1
...............................aggacgggaagaAaggagggcg...	3	1	pl1
...............................aggacgggaagagaggaCggcg...	2	1	pl1
...............................aggacggAaagagaggagggcg...	3	1	pl1
...............................aggacgggaagagaggaggUcg...	2	1	pl1
...............................aggacgggaagagaCgagggcg...	1	1	pl1

```
                              Star                                  Mature
agccgagucggagcugucggaggcgagggcgaggacgggaagagaggagggcgugguuucugcugguccucacuccucuccucccgucuuccuccuccuccucccauuccca
...............................aggacgggaagaCaggagggcg...........................................................	1	1	pl1
...............................aggacgggaagagaggagggcC...........................................................	1	1	pl1
...............................aggacgggaagagagCagggcg...........................................................	4	1	pl1
...............................Uggacgggaagagaggagggcg...........................................................	3	1	pl1
...............................aggacgggaagagaggaUggcg...........................................................	2	1	pl1
...............................aggacgggaagagUggagggcg...........................................................	1	1	pl1
...............................aggacgggaagagaggagggcUu..........................................................	6	1	pl1
...............................aggacgggaagagaggaggCcgu..........................................................	2	1	pl1
...............................Gggacgggaagagaggagggcgu..........................................................	1	1	pl1
...............................aggUcgggaagagaggagggcgu..........................................................	1	1	pl1
...............................aggacgggaagagaggagggcCu..........................................................	1	1	pl1
...............................aggacgggaagGgaggagggcgu..........................................................	1	1	pl1
...............................Cggacgggaagagaggagggcgu..........................................................	1	1	pl1
...............................aggacgggaagagaggagggAgu..........................................................	2	1	pl1
...............................aggacgggaagagaggagggcgu..........................................................	236	0	pl1
...............................aggacgggaagagaggNgggcgu..........................................................	2	1	pl1
...............................aggacgggaagUgaggagggcgu..........................................................	1	1	pl1
...............................Uggacgggaagagaggagggcgu..........................................................	2	1	pl1
...............................aggacgggaagagaggagggcgA..........................................................	124	1	pl1
...............................aggacgggaagagaggagggcgG..........................................................	2	1	pl1
...............................aggacgAgaagagaggagggcgu..........................................................	1	1	pl1
...............................aggacgggaagagaggaggAcgu..........................................................	3	1	pl1
...............................aggacgggCagagaggagggcgu..........................................................	1	1	pl1
...............................aggacgggaagagagCagggcgu..........................................................	1	1	pl1
...............................aggacgggaagagaggagggcgAg.........................................................	9	1	pl1
...............................aggacgggaagagaggagggcgug.........................................................	8	0	pl1
...............................aggacgggaagagaggagggcguU.........................................................	41	1	pl1
...............................aggacgggaagagaggagggcguA.........................................................	18	1	pl1
...............................aggacgUgaagagaggagggcgugg........................................................	1	1	pl1
...............................aggacgggaagagaggagggcgAgg........................................................	5	1	pl1
...............................aggacgggaagagaCgagggcgugg........................................................	1	1	pl1
...............................aggacgggaagagaggagggcgugg........................................................	46	0	pl1
...............................aggacgggaagagaggagggcguAg........................................................	4	1	pl1
...............................aggacgggaagagaggagggcgugU........................................................	1	1	pl1
...............................aggacgggaagagaggagggcgugA........................................................	3	1	pl1
...............................aggacgggaagagaggagggcgNgg........................................................	1	1	pl1
...............................aggacgNgaagagaggagggcgugg........................................................	1	1	pl1
................................ggacgggaagagaggagggcg...........................................................	3	0	pl1
................................ggacgggaagagaggagggcgu..........................................................	1	0	pl1
................................ggacgggaagagaggagggcgugg........................................................	4	0	pl1
..................................acgggaagagaggagggcgugg........................................................	3	0	pl1
...................................cgggaagagaggagggcgugg........................................................	2	0	pl1
....................................................................cucacuccucuccucccgucuu......................	2	0	pl1
....................................................................Uucacuccucuccucccgucuuc.....................	2	1	pl1
....................................................................Uucacuccucuccucccgucuucc....................	6	1	pl1
....................................................................cucacuccucuccucccgucuucc....................	1	0	pl1
....................................................................Uucacuccucuccucccgucuuccu...................	3	1	pl1
.....................................................................ucacuccucuccucccgu.........................	17	0	pl1
.....................................................................ucacuccucuccCcccgu.........................	1	1	pl1
.....................................................................ucacuccucuccucccguc........................	43	0	pl1
.....................................................................ucacuccucuccucccguA........................	2	1	pl1
.....................................................................ucacuccucuccucccNucu.......................	5	1	pl1
.....................................................................ucacuccucuccucccgucu.......................	342	0	pl1
.....................................................................Acacuccucuccucccgucu.......................	1	1	pl1
.....................................................................ucacuccucuccuUccgucu.......................	1	1	pl1
.....................................................................ucacuccucuccucccgucA.......................	18	1	pl1
.....................................................................Ncacuccucuccucccgucu.......................	1	1	pl1
.....................................................................ucacuccucuAcucccgucuu......................	6	1	pl1
.....................................................................ucacucNucuccucccgucuu......................	2	1	pl1
.....................................................................ucacuccucuccucccgGcuu......................	1	1	pl1
.....................................................................ucacuccucuccucccNucuu......................	2	1	pl1
.....................................................................ucacuccucuccucccgucuC......................	1	1	pl1
.....................................................................ucacuccucuccucccgucuu......................	455	0	pl1
.....................................................................ucacuccucuccuUccgucuu......................	2	1	pl1
.....................................................................ucacucUucuccucccgucuu......................	1	1	pl1
.....................................................................ucacuccucuccucccgucuA......................	16	1	pl1
.....................................................................ucacuccucuccucccCucuu......................	1	1	pl1
.....................................................................ucacuccucuccucUcgucuuc.....................	5	1	pl1
.....................................................................ucacuccucuccucccguAuuc.....................	2	1	pl1
.....................................................................uAacuccucuccucccgucuuc.....................	1	1	pl1
```

Star Mature

agccgagucggagcugucggaggcgagggcgaggacgggaagagaggagggcgugguuucugcugguccucacuccucuccucccgucuuccuccuccuccucccauuccca			
....ucacuccucuccuUccgucuuc.....................	9	1	pl1
....ucacuccucuccucccgucuuG.....................	7	1	pl1
....Acacuccucuccucccgucuuc.....................	5	1	pl1
....ucacuccucucGucccgucuuc.....................	1	1	pl1
....ucacCccucuccucccgucuuc.....................	1	1	pl1
....uUacuccucuccucccgucuuc.....................	2	1	pl1
....ucacuccCcuccucccgucuuc.....................	15	1	pl1
....ucacuccucuccucccUucuuc.....................	4	1	pl1
....ucacuccucuccAcccgucuuc.....................	2	1	pl1
....ucacuccucuccuccUgucuuc.....................	6	1	pl1
....ucacuccucuccucccNucuuc.....................	44	1	pl1
....ucacuccucuccucccgGcuuc.....................	3	1	pl1
....ucacuccucuccucccgAcuuc.....................	1	1	pl1
....ucacuccucucUucccgucuuc.....................	9	1	pl1
....ucacuccuUuccucccgucuuc.....................	9	1	pl1
....ucacucNucuccucccgucuuc.....................	37	1	pl1
....ucacuccucuccucccgucuAc.....................	1	1	pl1
....ucacuccucuccucccgucuuc.....................	6724	0	pl1
....ucacuGcucuccucccgucuuc.....................	1	1	pl1
....ucacuccucuccucccgCcuuc.....................	4	1	pl1
....ucacuccucuccucccAucuuc.....................	5	1	pl1
....Ncacuccucuccucccgucuuc.....................	14	1	pl1
....ucacuccucuccucccguGuuc.....................	1	1	pl1
....ucacuccucuccucccCucuuc.....................	15	1	pl1
....ucaUuccucuccucccgucuuc.....................	1	1	pl1
....ucacuccucuUcucccgucuuc.....................	8	1	pl1
....ucacuccucuccCcccgucuuc.....................	1	1	pl1
....ucacuAcucuccucccgucuuc.....................	3	1	pl1
....ucacuccucuccucccguUuuc.....................	5	1	pl1
....ucacuccucuccucccgucuCc.....................	4	1	pl1
....ucacucUucuccucccgucuuc.....................	3	1	pl1
....ucacuccucuccucccgucuuA.....................	86	1	pl1
....ucacuccucuccucccgucuuU.....................	156	1	pl1
....ucacuAcucuccucccgucuucc....................	1	1	pl1
....Gcacuccucuccucccgucuucc....................	2	1	pl1
....ucCcuccucuccucccgucuucc....................	2	1	pl1
....ucacuccucuccuUccgucuucc....................	16	1	pl1
....ucacuccucuccucccgGcuucc....................	13	1	pl1
....ucacuccucuccucccgucCucc....................	1	1	pl1
....ucacuccucuccucccAucuucc....................	28	1	pl1
....ucGcuccucuccucccgucuucc....................	1	1	pl1
....ucacucAucuccucccgucuucc....................	1	1	pl1
....ucacuccucuccucccgucuAcc....................	3	1	pl1
....ucacuccucuccucccgucuucA....................	1113	1	pl1
....ucacuccucuccucccguUuucc....................	6	1	pl1
....Ccacuccucuccucccgucuucc....................	11	1	pl1
....ucacuccucuccucccCucuucc....................	63	1	pl1
....ucacuccucuccuGccgucuucc....................	5	1	pl1
....uAacuccucuccucccgucuucc....................	4	1	pl1
....ucacuccucuccucccgAcuucc....................	6	1	pl1
....ucacuccucuccucccNucuucc....................	186	1	pl1
....ucacuccucuccuccUgucuucc....................	13	1	pl1
....Ncacuccucuccucccgucuucc....................	51	1	pl1
....ucacucUucuccucccgucuucc....................	18	1	pl1
....ucacuUcucuccucccgucuucc....................	3	1	pl1
....ucacuccucuccucccgucuucU....................	1190	1	pl1
....ucacuccucuccucUcgucuucc....................	18	1	pl1
....ucacuccuUuccucccgucuucc....................	23	1	pl1
....ucacuccucuccAcccgucuucc....................	4	1	pl1
....ucacuccAcuccucccgucuucc....................	4	1	pl1
....ucacuccucuccCcccgucuucc....................	6	1	pl1
....ucacuccucuccucGcgucuucc....................	4	1	pl1
....ucacucNucuccucccgucuucc....................	114	1	pl1
....ucacuccucuccucccgucuCcc....................	21	1	pl1
....ucacuccucuGcucccgucuucc....................	1	1	pl1
....ucacCccucuccucccgucuucc....................	2	1	pl1
....ucacuccucCccucccgucuucc....................	1	1	pl1
....ucacuccucAccucccgucuucc....................	1	1	pl1
....ucacuccucuccucccgucuucc....................	24526	0	pl1
....ucacuccCcuccucccgucuucc....................	5	1	pl1

```
                                 Star                    Mature
agccgagucggagcugucggaggcgagggcgaggacgggaagagaggagggcgugguuucugcugguccucacuccucuccucccgucuuccuccuccuccucccauuccca
.....................................................................ucacuccucuAcucccgucuucc....................  4     1  pl1
.....................................................................uUacuccucuccucccgucuucc....................  11    1  pl1
.....................................................................ucacuccucuccucccgucAucc....................  3     1  pl1
.....................................................................ucacuccucucUucccgucuucc....................  18    1  pl1
.....................................................................ucacuccucuccucccgucuuUc....................  12    1  pl1
.....................................................................ucacuccuGuccucccgucuucc....................  3     1  pl1
.....................................................................Acacuccucuccucccgucuucc....................  21    1  pl1
.....................................................................ucacuccucuccucccUucuucc....................  15    1  pl1
.....................................................................ucacuccucuccucccgCcuucc....................  22    1  pl1
.....................................................................ucacuccucuccGcccgucuucc....................  2     1  pl1
.....................................................................ucacAccucuccucccgucuucc....................  3     1  pl1
.....................................................................ucaUuccucuccucccgucuucc....................  11    1  pl1
.....................................................................ucUcuccucuccucccgucuucc....................  8     1  pl1
.....................................................................ucacuccucuccucccgucuucG....................  64    1  pl1
.....................................................................ucacuccucuccucccgucuuGc....................  1     1  pl1
.....................................................................ucacuccucGccucccgucuucc....................  1     1  pl1
.....................................................................ucacuccucuUcucccgucuucc....................  17    1  pl1
.....................................................................ucacuccucuccucccgucGucc....................  2     1  pl1
.....................................................................ucaUuccucuccucccgucuuccu...................  1     1  pl1
.....................................................................ucacuccucuccuccUgucuuccu...................  1     1  pl1
.....................................................................ucacuccucuccucccgCcuuccu...................  1     1  pl1
.....................................................................ucacuccucuccucccgucuuccu...................  2772  0  pl1
.....................................................................ucacuccCcuccucccgucuuccu...................  2     1  pl1
.....................................................................ucacucNucuccucccgucuuccu...................  8     1  pl1
.....................................................................ucacuccuUuccucccgucuuccu...................  2     1  pl1
.....................................................................ucacuccucuccucccgucCuccu...................  2     1  pl1
.....................................................................ucacuccucuccucccgucuucGu...................  6     1  pl1
.....................................................................ucacuccucuccucccgucuuccA...................  2347  1  pl1
.....................................................................ucacuccucuccucAcgucuuccu...................  1     1  pl1
.....................................................................ucacuccucuccucUcgucuuccu...................  3     1  pl1
.....................................................................ucacuccucuccucccgucAuccu...................  1     1  pl1
.....................................................................ucacuccucuccucccAucuuccu...................  5     1  pl1
.....................................................................ucacuccucuccucccgucuuccG...................  68    1  pl1
.....................................................................ucacuccucuccucccCucuuccu...................  8     1  pl1
.....................................................................ucacuccucuccucccUucuuccu...................  2     1  pl1
.....................................................................ucacuccucuccucccgucuucNu...................  23    1  pl1
.....................................................................ucacuGcucuccucccgucuuccu...................  1     1  pl1
.....................................................................ucacucAucuccucccgucuuccu...................  1     1  pl1
.....................................................................Ncacuccucuccucccgucuuccu...................  6     1  pl1
.....................................................................ucacuccucuccucccgucuuccC...................  11    1  pl1
.....................................................................Acacuccucuccucccgucuuccu...................  3     1  pl1
.....................................................................ucacuccucuccucccgucuuUcu...................  1     1  pl1
.....................................................................ucacuccucuccucccNucuuccu...................  21    1  pl1
.....................................................................ucacuccucuccucccgucuucAu...................  44    1  pl1
.....................................................................ucacuccucucUucccgucuuccu...................  2     1  pl1
.....................................................................ucacuccucuccCcccgucuuccu...................  1     1  pl1
.....................................................................ucacuccucuccucccgGcuuccu...................  5     1  pl1
.....................................................................ucacucUucuccucccgucuuccu...................  1     1  pl1
.....................................................................ucacuccucuccucccgAcuuccu...................  1     1  pl1
.....................................................................ucacuccucuccuUccgucuuccu...................  2     1  pl1
.....................................................................ucacuccucuccucccgucuucUu...................  51    1  pl1
.....................................................................ucacuccucuccucccgucuuccAc..................  1     1  pl1
.....................................................................ucacuccucuccucccgucuuccuU..................  195   1  pl1
.....................................................................ucacuccucuccucccgucuuccuG..................  3     1  pl1
.....................................................................ucacuccucuccucccgucuuccuA..................  56    1  pl1
......................................................................cacuccucuccucccguc........................  1     0  pl1
......................................................................cacuccucuccucccgucA.......................  2     1  pl1
......................................................................cacuccucuccucccgucu.......................  10    0  pl1
......................................................................cacuccucucUucccgucuu......................  1     1  pl1
......................................................................cacuccucuccuUccgucuu......................  1     1  pl1
......................................................................cacuccucuccucccgucuu......................  41    0  pl1
......................................................................cacuccucuccucccgucuuU.....................  15    1  pl1
......................................................................cacucUucuccucccgucuuc.....................  1     1  pl1
......................................................................cacuccucuccucccgCcuuc.....................  1     1  pl1
......................................................................cacuccucuccucccgGcuuc.....................  2     1  pl1
......................................................................cacuccucuccucccCucuuc.....................  4     1  pl1
......................................................................Uacuccucuccucccgucuuc.....................  1     1  pl1
......................................................................cacuccNcuccucccgucuuc.....................  7     1  pl1
......................................................................cacuccucuccucccgucuuA.....................  10    1  pl1
......................................................................caUuccucuccucccgucuuc.....................  6     1  pl1
```

```
                              Star                                    Mature
agccgagucggagcugucggaggcgagggcgaggacgggaagagaggagggcgugguuucugcugguccucacuccucuccucccgucuuccuccuccuccucccauuccca
......................................................................cacuccucucUucccgucuuc.....................    1       1       pl1
......................................................................cacuccucuccucccgucuuc.....................    1144    0       pl1
......................................................................cacuccucuccucAcgucuuc.....................    1       1       pl1
......................................................................cacuccucuccucccgAcuuc.....................    3       1       pl1
......................................................................cacuccucuccuUccgucuuc.....................    3       1       pl1
......................................................................cacuccucuccucccguGuuc.....................    1       1       pl1
......................................................................cacuccucuUcucccgucuuc.....................    2       1       pl1
......................................................................cacuccucuccucccgNcuuc.....................    8       1       pl1
......................................................................cacuccucuAcucccgucuuc.....................    1       1       pl1
......................................................................Nacuccucuccucccgucuuc.....................    1       1       pl1
......................................................................cacuccNcuccucccgucuucc....................    58      1       pl1
......................................................................Gacuccucuccucccgucuucc....................    2       1       pl1
......................................................................cacucGucuccucccgucuucc....................    2       1       pl1
......................................................................cacuccucuUcucccgucuucc....................    16      1       pl1
......................................................................cacuccucuccucccgucAucc....................    2       1       pl1
......................................................................Nacuccucuccucccgucuucc....................    22      1       pl1
......................................................................cacuccucuccCcccgucuucc....................    3       1       pl1
......................................................................cacuccucuccucccAucuucc....................    10      1       pl1
......................................................................cacuccucuccucccgCcuucc....................    12      1       pl1
......................................................................cacuccucucAucccgucuucc....................    5       1       pl1
......................................................................cacuccGcuccucccgucuucc....................    1       1       pl1
......................................................................cacuccucuccucccgucCucc....................    3       1       pl1
......................................................................cacuccucuccucAcgucuucc....................    2       1       pl1
......................................................................cacuccucuccucccgucuuUc....................    2       1       pl1
......................................................................cacuccucuccucccgucuucU....................    114     1       pl1
......................................................................cacCccucuccucccgucuucc....................    3       1       pl1
......................................................................cacuccucuccucccgucuCcc....................    10      1       pl1
......................................................................cacuccucuccucccUucuucc....................    4       1       pl1
......................................................................cacuccucuccucccgAcuucc....................    14      1       pl1
......................................................................cacucUucuccucccgucuucc....................    1       1       pl1
......................................................................cacuccucuccucccguUuucc....................    3       1       pl1
......................................................................cacuccucuccuccUgucuucc....................    12      1       pl1
......................................................................cacuccucuccucccgGcuucc....................    4       1       pl1
......................................................................cacuccucuccucccgucuucc....................    11137   0       pl1
......................................................................cacuccucucUucccgucuucc....................    17      1       pl1
......................................................................cacuccucuccucccCucuucc....................    20      1       pl1
......................................................................cacuccucuccucccgucuucA....................    114     1       pl1
......................................................................cacuccucuccucccgNcuucc....................    89      1       pl1
......................................................................Aacuccucuccucccgucuucc....................    2       1       pl1
......................................................................cacuccCcuccucccgucuucc....................    4       1       pl1
......................................................................cacuccuUuccucccgucuucc....................    1       1       pl1
......................................................................caUuccucuccucccgucuucc....................    7       1       pl1
......................................................................cacuccucuccucccgucuucG....................    15      1       pl1
......................................................................cacuccucuccucUcgucuucc....................    5       1       pl1
......................................................................cGcuccucuccucccgucuucc....................    1       1       pl1
......................................................................cUcuccucuccucccgucuucc....................    3       1       pl1
......................................................................cacuccucCccucccgucuucc....................    3       1       pl1
......................................................................cacuccucAccucccgucuucc....................    1       1       pl1
......................................................................Uacuccucuccucccgucuucc....................    8       1       pl1
......................................................................cacuccucuccuUccgucuucc....................    18      1       pl1
......................................................................cCcuccucuccucccgucuucc....................    1       1       pl1
......................................................................cacuccucuccucccguGuucc....................    2       1       pl1
......................................................................cacuccucuccuGccgucuuccu...................    1       1       pl1
......................................................................cacuccucuccucccgucuuccC...................    2       1       pl1
......................................................................cacuccucuUcucccgucuuccu...................    1       1       pl1
......................................................................cacuccNcuccucccgucuuccu...................    2       1       pl1
......................................................................cacuccucuccucccgNcuuccu...................    6       1       pl1
......................................................................cacuccucuccucccgucuucAu...................    3       1       pl1
......................................................................cacuccucuccucccgucuuccA...................    1280    1       pl1
......................................................................cacuUcucuccucccgucuuccu...................    1       1       pl1
......................................................................cacuccucuccucccgGcuuccu...................    1       1       pl1
......................................................................cacuccucuccucccgucuuccG...................    14      1       pl1
......................................................................cacuccucuccucccgAcuuccu...................    1       1       pl1
......................................................................cacuccucuccucccgucuucUu...................    1       1       pl1
......................................................................cacuccucuccucccgucuuccu...................    401     0       pl1
......................................................................cacuccucuccucccgucuuccuU..................    25      1       pl1
......................................................................cacuccucuccucccgucuuccuA..................    21      1       pl1
.......................................................................acuccucuccucccgucuuc.....................    2       0       pl1
.......................................................................acuccucuccucccgucuucc....................    2       0       pl1
.......................................................................acuccucuccucccgucuuccu...................    5       0       pl1
```

```
                              Star                                       Mature
agccgagucggagcugucggaggcgagggcgaggacgggaagagaggagggcgugguuucugcugguccucacuccucuccucccgucuuccuccuccuccucccauuccca
........................................................................cuccucuccucccgucuucc....................  2    0  pl1
.........................................................................uccucuccucccgucuucc....................  7    0  pl1
..........................................................................ccucuccucccgucuucc....................  3    0  pl1
...........................................................................cucuccucccgucuuccu...................  1    0  pl1
......................................................................................ucuuccuccuccucGucc........  3    1  pl1

..........gagcugucggaggcgagggcg.................................................................................  7    0  pl2
...........agcugucggaggcgagggcg.................................................................................  2    0  pl2
...........agcugucggaggcgagggcga................................................................................  1    0  pl2
............gcugucggaggcgagggcg.................................................................................  3    0  pl2
.............cugucggaggcgagggcg.................................................................................  34   0  pl2
.............cugucggaggcgagggNg.................................................................................  1    1  pl2
..............................Uaggacgggaagagaggagggcg...........................................................  1    1  pl2
...............................aggaUgggaagagaggag...............................................................  1    1  pl2
...............................aggacgggaagagaggaU...............................................................  1    1  pl2
...............................Gggacgggaagagaggag...............................................................  1    1  pl2
...............................aggacgggaagagaggUg...............................................................  2    1  pl2
...............................aggacgggaagagaggGg...............................................................  1    1  pl2
...............................aggacgCgaagagaggag...............................................................  1    1  pl2
...............................aggacgggaagagagCag...............................................................  1    1  pl2
...............................aggacgAgaagagaggag...............................................................  1    1  pl2
...............................aggacgNgaagagaggag...............................................................  1    1  pl2
...............................aggacgggUagagaggag...............................................................  1    1  pl2
...............................aggacAggaagagaggag...............................................................  1    1  pl2
...............................aggacgggaagagaggag...............................................................  152  0  pl2
...............................aggacgggaagagaCgag...............................................................  2    1  pl2
...............................aggacgCgaagagaggagg..............................................................  2    1  pl2
...............................aggacggCaagagaggagg..............................................................  2    1  pl2
...............................aggacgggaaAagaggagg..............................................................  1    1  pl2
...............................aggacgggaagagaCgagg..............................................................  2    1  pl2
...............................aggacgggaagagaggUgg..............................................................  1    1  pl2
...............................agCacgggaagagaggagg..............................................................  1    1  pl2
...............................aggacCggaagagaggagg..............................................................  2    1  pl2
...............................aggacgggaagagaggagA..............................................................  30   1  pl2
...............................aggacgggaagagaggagU..............................................................  41   1  pl2
...............................aggacgNgaagagaggagg..............................................................  1    1  pl2
...............................aggacgggaagagaggaAg..............................................................  1    1  pl2
...............................aggacgggCagagaggagg..............................................................  3    1  pl2
...............................aggacgggaagagaggagg..............................................................  227  0  pl2
...............................aggacgggaaCagaggagg..............................................................  1    1  pl2
...............................aggacgggaagagaCgaggg.............................................................  1    1  pl2
...............................aggacgggaagCgaggaggg.............................................................  1    1  pl2
...............................aggacgggaagUgaggaggg.............................................................  1    1  pl2
...............................aggacAggaagagaggaggg.............................................................  1    1  pl2
...............................agCacgggaagagaggaggg.............................................................  1    1  pl2
...............................aggacgggaagagaggNggg.............................................................  1    1  pl2
...............................aggaUgggaagagaggaggg.............................................................  1    1  pl2
...............................aggacgggCagagaggaggg.............................................................  1    1  pl2
...............................aggacgggaagagaggaggU.............................................................  8    1  pl2
...............................aggacgggaagaCaggaggg.............................................................  1    1  pl2
...............................aggacggCaagagaggaggg.............................................................  1    1  pl2
...............................aggacgggaagagaggaggg.............................................................  189  0  pl2
...............................aggacgggaagagaggaAgg.............................................................  1    1  pl2
...............................aggUcgggaagagaggaggg.............................................................  1    1  pl2
...............................aggacgggaagagagCaggg.............................................................  1    1  pl2
...............................aggacgggaagagaggCggg.............................................................  1    1  pl2
...............................aggacgggaagagUggaggg.............................................................  1    1  pl2
...............................aggacgUgaagagaggaggg.............................................................  1    1  pl2
...............................aggacgggaaAagaggaggg.............................................................  1    1  pl2
...............................aggacgggaagagaggaggA.............................................................  11   1  pl2
...............................aggacgggGagagaggaggg.............................................................  1    1  pl2
...............................aggacgggaagagaggagAg.............................................................  1    1  pl2
...............................Nggacgggaagagaggaggg.............................................................  2    1  pl2
...............................aggacgggCagagaggagggc............................................................  1    1  pl2
...............................aggacgggaagagaggaggAc............................................................  2    1  pl2
...............................Nggacgggaagagaggagggc............................................................  1    1  pl2
...............................aggacgggaagagaggaggCc............................................................  1    1  pl2
...............................aggacgggaagagaggagggU............................................................  25   1  pl2
...............................aCgacgggaagagaggagggc............................................................  1    1  pl2
...............................Gggacgggaagagaggagggc............................................................  1    1  pl2
```

Star **Mature**

agccgagucggagcugucggaggcgagggcgaggacgggaagagaggagggcgugguuucugcugguccucacuccucuccucccgucuuccuccuccuccucccauuccca			
...............................aggacgggaagagagCagggc..	1	1	pl2
...............................aggacgggaagagaggUgggc..	1	1	pl2
...............................aggacgggaagagaggNgggc..	1	1	pl2
...............................aggacgCgaagagaggagggc..	3	1	pl2
...............................aggacCggaagagaggagggc..	1	1	pl2
...............................aggacggUaagagaggagggc..	2	1	pl2
...............................aggacgggaagagaggGgggc..	2	1	pl2
...............................aggacgNgaagagaggagggc..	1	1	pl2
...............................aggacgggaagagaggaUggc..	1	1	pl2
...............................aggacgggaaCagaggagggc..	1	1	pl2
...............................aggacgUgaagagaggagggc..	1	1	pl2
...............................aggacgggaagUgaggagggc..	1	1	pl2
...............................aggacgggaagagaggagggc..	324	0	pl2
...............................Cggacgggaagagaggagggc..	1	1	pl2
...............................aggacggAaagagaggagggc..	2	1	pl2
...............................Uggacgggaagagaggagggc..	1	1	pl2
...............................aggacgggaagCgaggagggcg...	2	1	pl2
...............................aggaUgggaagagaggagggcg...	1	1	pl2
...............................aggacgggaagagGggagggcg...	1	1	pl2
...............................aggaGgggaagagaggagggcg...	1	1	pl2
...............................aggacgggaagGgaggagggcg...	2	1	pl2
...............................aggCcgggaagagaggagggcg...	1	1	pl2
...............................aggacgggaagagaggCgggcg...	3	1	pl2
...............................aggacgggaagagaggagAgcg...	2	1	pl2
...............................aggacgggaagagaUgagggcg...	1	1	pl2
...............................aggGcgggaagagaggagggcg...	1	1	pl2
...............................Cggacgggaagagaggagggcg...	4	1	pl2
...............................aggacggCaagagaggagggcg...	3	1	pl2
...............................aggacgggaagagaggagCgcg...	1	1	pl2
...............................aggacgCgaagagaggagggcg...	7	1	pl2
...............................aCgacgggaagagaggagggcg...	1	1	pl2
...............................aggacAggaagagaggagggcg...	2	1	pl2
...............................aggacgggaagagaggNgggcg...	6	1	pl2
...............................aggacgggaagagaggaggCcg...	3	1	pl2
...............................aggacgUgaagagaggagggcg...	1	1	pl2
...............................aggacgggaagagaggaggAcg...	8	1	pl2
...............................aggacgggaaAagaggagggcg...	1	1	pl2
...............................aggacgggaagagaggagggcg...	2023	0	pl2
...............................aggacgggCagagaggagggcg...	6	1	pl2
...............................aggacgggaagagUggagggcg...	3	1	pl2
...............................aggacgggaagagaggaggUcg...	2	1	pl2
...............................aggacgggaagagaggagggcU...	33	1	pl2
...............................aUgacgggaagagaggagggcg...	1	1	pl2
...............................aggacgggaagUgaggagggcg...	7	1	pl2
...............................aggacgggaGgagaggagggcg...	1	1	pl2
...............................aggacgggaagagaggagggcA...	6	1	pl2
...............................aggacUggaagagaggagggcg...	1	1	pl2
...............................Uggacgggaagagaggagggcg...	3	1	pl2
...............................aggacgggUagagaggagggcg...	1	1	pl2
...............................aggacgggaagagaggUgggcg...	2	1	pl2
...............................aggacgggaaCagaggagggcg...	1	1	pl2
...............................aggacgggaagagaggagggcC...	3	1	pl2
...............................aggUcgggaagagaggagggcg...	1	1	pl2
...............................aggacgggGagagaggagggcg...	4	1	pl2
...............................aggacgggaagagCggagggcg...	1	1	pl2
...............................aggacgNgaagagaggagggcg...	9	1	pl2
...............................aggacgggaagagaggaCggcg...	2	1	pl2
...............................agCacgggaagagaggagggcg...	3	1	pl2
...............................aggacgggaagagaggagggUg...	4	1	pl2
...............................Nggacgggaagagaggagggcg...	3	1	pl2
...............................aggacggAaagagaggagggcg...	3	1	pl2
...............................aggacgggaagagagCagggcg...	6	1	pl2
...............................aggacCggaagagaggagggcg...	5	1	pl2
...............................aggacgggaagagaggGgggcg...	1	1	pl2
...............................aggacgggaagagaggagggcgC..	1	1	pl2
...............................aggacgggaagagaggagggcgu..	241	0	pl2
...............................aggacgggaagagaCgagggcgu..	1	1	pl2
...............................aggacggAaagagaggagggcgu..	1	1	pl2
...............................aggacgCgaagagaggagggcgu..	2	1	pl2
...............................aggacgggaagagaggNgggcgu..	2	1	pl2

Star **Mature**

```
agccgagucggagcugucggaggcgagggcgaggacgggaagagaggagggcgugguuucugcugguccucacuccucuccucccgucuuccuccuccuccucccauuccca
...............................aggacgggaagagaggaggUcgu..........................................................   1     1   pl2
...............................aggacgggaagagaggUgggcgu..........................................................   1     1   pl2
...............................aggacgggGagagaggagggcgu..........................................................   1     1   pl2
...............................aggacgggaagaUaggagggcgu..........................................................   1     1   pl2
...............................aggacgggaagagaggaggCcgu..........................................................   2     1   pl2
...............................aggacgggaagagaggagggcAu..........................................................   4     1   pl2
...............................aggacgNgaagagaggagggcgu..........................................................   3     1   pl2
...............................aggacgggaagagaggagggcgA..........................................................   108   1   pl2
...............................aggacgggaagagaggagggcUu..........................................................   8     1   pl2
...............................aggacgggaagagaggagggcgG..........................................................   2     1   pl2
...............................aggacgggaagagaggagggcgNg.........................................................   1     1   pl2
...............................aggacgggaagagaggagggcgug.........................................................   5     0   pl2
...............................aggacgggaagagaggagggcguU.........................................................   33    1   pl2
...............................aggacgggUagagaggagggcgug.........................................................   1     1   pl2
...............................aggacgggaagagaggagggcguA.........................................................   11    1   pl2
...............................aggacgggaagagaggagggcgAg.........................................................   19    1   pl2
...............................aggacgggaagagaggagggcguAg........................................................   8     1   pl2
...............................aggacgggaagagaggagggcguUg........................................................   1     1   pl2
...............................aggacAggaagagaggagggcgugg........................................................   1     1   pl2
...............................aggacgggaagagaggagggcgAgg........................................................   1     1   pl2
...............................agCacgggaagagaggagggcgugg........................................................   1     1   pl2
...............................aggacgggaagagaggagggcgugA........................................................   1     1   pl2
...............................aggacgggaagagaggagggcgugg........................................................   35    0   pl2
................................ggacgggaagagaggagg..............................................................   1     0   pl2
................................ggacgggaagagagCagg..............................................................   1     1   pl2
................................ggacgggaagagaggagggcg...........................................................   1     0   pl2
................................ggacgggaagagaggagggcguA.........................................................   1     1   pl2
...................................cgggaagagaggagggcgugg........................................................   1     0   pl2
.....................................................ugguuucugcugguccuU.........................................   2     1   pl2
....................................................................Aucacuccucuccucccgucuucc....................   1     1   pl2
.....................................................................ucacuccucuccucccgu.........................   10    0   pl2
.....................................................................ucacuccucuccucccguA........................   1     1   pl2
.....................................................................ucacuccucuUcucccguc........................   1     1   pl2
.....................................................................ucacuccucuccucccguc........................   15    0   pl2
.....................................................................Ccacuccucuccucccgucu.......................   1     1   pl2
.....................................................................ucacuccucucUucccgucu.......................   1     1   pl2
.....................................................................Ncacuccucuccucccgucu.......................   1     1   pl2
.....................................................................ucacuccucuccucccgucA.......................   5     1   pl2
.....................................................................ucacuccucuccGcccgucu.......................   1     1   pl2
.....................................................................ucacucNucuccucccgucu.......................   2     1   pl2
.....................................................................ucacuccucuccucccgucu.......................   142   0   pl2
.....................................................................ucacuccucuccucccgCcu.......................   1     1   pl2
.....................................................................ucacuccucuccucccCucuu......................   1     1   pl2
.....................................................................ucacuccucuccucccgGcuu......................   1     1   pl2
.....................................................................ucacuccucuccucccgucuA......................   4     1   pl2
.....................................................................ucacucNucuccucccgucuu......................   1     1   pl2
.....................................................................ucacuccAcuccucccgucuu......................   1     1   pl2
.....................................................................ucacuccucuccucccgucuu......................   204   0   pl2
.....................................................................Ncacuccucuccucccgucuu......................   1     1   pl2
.....................................................................ucacuccucuccucccgucuuG.....................   7     1   pl2
.....................................................................ucacuccucuGcucccgucuuc.....................   4     1   pl2
.....................................................................ucacuccucuUcucccgucuuc.....................   2     1   pl2
.....................................................................ucacuccucuccucccNucuuc.....................   13    1   pl2
.....................................................................ucacuccucuccucccgCcuuc.....................   2     1   pl2
.....................................................................ucacuccucuccucccgucuuU.....................   42    1   pl2
.....................................................................ucaUuccucuccucccgucuuc.....................   5     1   pl2
.....................................................................ucacucNucuccucccgucuuc.....................   11    1   pl2
.....................................................................ucacuccucuccuUccgucuuc.....................   10    1   pl2
.....................................................................ucacuccucuccucccgucuGc.....................   1     1   pl2
.....................................................................ucacuccucuccucccUucuuc.....................   1     1   pl2
.....................................................................Acacuccucuccucccgucuuc.....................   3     1   pl2
.....................................................................ucaAuccucuccucccgucuuc.....................   2     1   pl2
.....................................................................ucacuccCcuccucccgucuuc.....................   5     1   pl2
.....................................................................ucacuccucuccucccgucuuc.....................   2578  0   pl2
.....................................................................ucacuccucuccuccUgucuuc.....................   2     1   pl2
.....................................................................ucacuccuUuccucccgucuuc.....................   2     1   pl2
.....................................................................ucacuccucuccucccCucuuc.....................   7     1   pl2
.....................................................................ucacuccucuccucccgAcuuc.....................   1     1   pl2
.....................................................................ucacuccucuccucccAucuuc.....................   2     1   pl2
.....................................................................ucacucUucuccucccgucuuc.....................   2     1   pl2
```

```
                               Star                                  Mature
agccgagucggagcugucggaggcgagggcgaggacgggaagagaggagggcgugguuucugcuggucuucacuccucuccucccgucuuccuccuccuccucccauuccca
.....................................................................ucacuccucuccucccgucuCc.....................    3      1    pl2
.....................................................................ucacuccucuccucccgucCuc.....................    1      1    pl2
.....................................................................Gcacuccucuccucccgucuuc.....................    1      1    pl2
.....................................................................Ccacuccucuccucccgucuuc.....................    1      1    pl2
.....................................................................ucacuccucuccucccgGcuuc.....................    1      1    pl2
.....................................................................ucacuccucuccucccgucuuA.....................    24     1    pl2
.....................................................................Ncacuccucuccucccgucuuc.....................    6      1    pl2
.....................................................................ucacuccucuccucccUucuucc....................    6      1    pl2
.....................................................................ucacuccucuccAcccgucuucc....................    1      1    pl2
.....................................................................ucacuccucuccCcccgucuucc....................    6      1    pl2
.....................................................................ucacuccucuccucccgucuucU....................    440    1    pl2
.....................................................................ucacuccuUuccucccgucuucc....................    10     1    pl2
.....................................................................ucacuccucuccucGcgucuucc....................    1      1    pl2
.....................................................................ucacuccucuccucAcgucuucc....................    1      1    pl2
.....................................................................ucacuUcucuccucccgucuucc....................    4      1    pl2
.....................................................................ucUcuccucuccucccgucuucc....................    1      1    pl2
.....................................................................ucacuccucuccucccgucuucG....................    9      1    pl2
.....................................................................ucacuccucuUcucccgucuucc....................    17     1    pl2
.....................................................................ucacuccucuccuAccgucuucc....................    1      1    pl2
.....................................................................ucacuccucuccucccgucCucc....................    6      1    pl2
.....................................................................ucacuccucuccucccgucGucc....................    1      1    pl2
.....................................................................Ncacuccucuccucccgucuucc....................    14     1    pl2
.....................................................................ucacuccucuccucccgucAucc....................    1      1    pl2
.....................................................................ucacuccucuccucccCucuucc....................    33     1    pl2
.....................................................................ucacuccucuccucccgucuucA....................    330    1    pl2
.....................................................................ucacuccucuccucccgucuuUc....................    1      1    pl2
.....................................................................ucacuccucuccucccgucuucc....................    10044  0    pl2
.....................................................................ucacuGcucuccucccgucuucc....................    1      1    pl2
.....................................................................uAacuccucuccucccgucuucc....................    1      1    pl2
.....................................................................ucacuccucCccucccgucuucc....................    4      1    pl2
.....................................................................ucacuccucuccucccAucuucc....................    16     1    pl2
.....................................................................ucacucUucuccucccgucuucc....................    9      1    pl2
.....................................................................ucacuccucuccucccNucuucc....................    60     1    pl2
.....................................................................ucacuccucuccucccgucuuGc....................    1      1    pl2
.....................................................................ucacucGucuccucccgucuucc....................    1      1    pl2
.....................................................................uUacuccucuccucccgucuucc....................    5      1    pl2
.....................................................................ucacuccAcuccucccgucuucc....................    1      1    pl2
.....................................................................ucacuccucuccucccgucuAcc....................    1      1    pl2
.....................................................................ucacucAucuccucccgucuucc....................    1      1    pl2
.....................................................................ucaUuccucuccucccgucuucc....................    6      1    pl2
.....................................................................ucacuccucucUucccgucuucc....................    19     1    pl2
.....................................................................ucacuccucuAcucccgucuucc....................    3      1    pl2
.....................................................................ucacuccucuccucccguUuucc....................    6      1    pl2
.....................................................................ucacuccucuccuccUgucuucc....................    2      1    pl2
.....................................................................ucacucNucuccucccgucuucc....................    41     1    pl2
.....................................................................ucacuccucuccucccguGuucc....................    1      1    pl2
.....................................................................ucacuccucuccucccgCcuucc....................    15     1    pl2
.....................................................................ucacuccucuccucUcgucuucc....................    6      1    pl2
.....................................................................ucacCccucuccucccgucuucc....................    3      1    pl2
.....................................................................ucacuccucuGcucccgucuucc....................    1      1    pl2
.....................................................................Acacuccucuccucccgucuucc....................    10     1    pl2
.....................................................................Ccacuccucuccucccgucuucc....................    5      1    pl2
.....................................................................ucacuccucuccucccgucuCcc....................    14     1    pl2
.....................................................................ucacuccCcuccucccgucuucc....................    7      1    pl2
.....................................................................ucacuccucuccucccgAcuucc....................    4      1    pl2
.....................................................................ucacuccucuccuGccgucuucc....................    1      1    pl2
.....................................................................ucacuccucuccuUccgucuucc....................    5      1    pl2
.....................................................................ucacuccucuccucccgGcuucc....................    14     1    pl2
.....................................................................ucacuccuGuccucccgucuucc....................    1      1    pl2
.....................................................................ucacuccucuccucccgucuuccA...................    696    1    pl2
.....................................................................ucacuccucuccucccgucuuccu...................    979    0    pl2
.....................................................................ucacuccucuccucccgucuucNu...................    9      1    pl2
.....................................................................ucacuccucuccucccgucuuUcu...................    2      1    pl2
.....................................................................ucacuccCcuccucccgucuuccu...................    1      1    pl2
.....................................................................ucacuccucuccucccgucuuccC...................    9      1    pl2
.....................................................................ucacuccucuccucccNucuuccu...................    6      1    pl2
.....................................................................ucacuccucuccucccUucuuccu...................    2      1    pl2
.....................................................................ucacuccucuccucccgucuucAu...................    11     1    pl2
.....................................................................ucacuccucuccucccCucuuccu...................    4      1    pl2
.....................................................................ucacuccucuccucccAucuuccu...................    1      1    pl2
```

```
                              Star                                    Mature
agccgagucggagcugucggaggcgagggcgaggacgggaagagaggagggcgugguuucugcugguccucacuccucuccucccgucuuccuccuccuccucccauuccca
.....................................................................ucacuccucuccuccUgucuuccu...................    1    1    pl2
.....................................................................ucacuccucuccucccgAcuuccu...................    1    1    pl2
.....................................................................ucacuccucucUucccgucuuccu...................    2    1    pl2
.....................................................................ucacucNucuccucccgucuuccu...................    2    1    pl2
.....................................................................ucacuccucuccucccgucuCccu...................    1    1    pl2
.....................................................................ucacuccuAuccucccgucuuccu...................    1    1    pl2
.....................................................................ucacuccucuccucccgucuuccG...................    23   1    pl2
.....................................................................Ncacuccucuccucccgucuuccu...................    6    1    pl2
.....................................................................ucacuccucuccucccgucuAccu...................    1    1    pl2
.....................................................................ucacuccucuUcucccgucuuccu...................    2    1    pl2
.....................................................................ucacuccucuccucccgucuucUu...................    17   1    pl2
.....................................................................ucacuccucuccucUcgucuuccu...................    2    1    pl2
.....................................................................ucacuccucuccucccgucCuccu...................    1    1    pl2
.....................................................................ucacuccucuccucccgucuuccuc..................    2    0    pl2
.....................................................................ucacuccucuccucccgucuuccuU..................    48   1    pl2
.....................................................................ucacuccucuccucccgucuuccuA..................    18   1    pl2
......................................................................cacuccucuccucccguc........................    4    0    pl2
......................................................................cacuccucuccucccgucu.......................    5    0    pl2
......................................................................cacuccNcuccucccgucuu......................    1    1    pl2
......................................................................cacuccucuccucccgucuu......................    14   0    pl2
......................................................................cacuccucuccucccgucuuU.....................    1    1    pl2
......................................................................cacuccucuccucccgucuuA.....................    1    1    pl2
......................................................................Nacuccucuccucccgucuuc.....................    2    1    pl2
......................................................................cacuccucuUcucccgucuuc.....................    1    1    pl2
......................................................................cacuccucuccucccgNcuuc.....................    2    1    pl2
......................................................................cacuccucuccucccguUuuc.....................    1    1    pl2
......................................................................cacucUucuccucccgucuuc.....................    1    1    pl2
......................................................................cacuccAcuccucccgucuuc.....................    2    1    pl2
......................................................................cacuccuGuccucccgucuuc.....................    1    1    pl2
......................................................................cacuccNcuccucccgucuuc.....................    3    1    pl2
......................................................................cacuccucuccucccgGcuuc.....................    1    1    pl2
......................................................................cacuccucuccucUcgucuuc.....................    1    1    pl2
......................................................................cacuccucuccucccgucuuc.....................    483  0    pl2
......................................................................cacuccucuccucccAucuucc....................    4    1    pl2
......................................................................cacuccucuccucccgucuucG....................    2    1    pl2
......................................................................Uacuccucuccucccgucuucc....................    1    1    pl2
......................................................................cacuccucuccucccgNcuucc....................    27   1    pl2
......................................................................cacucUucuccucccgucuucc....................    4    1    pl2
......................................................................cacuccucucUucccgucuucc....................    10   1    pl2
......................................................................cacuccucuccucccgucuucU....................    53   1    pl2
......................................................................cacuccucuccucccgucAucc....................    2    1    pl2
......................................................................cacuccCcuccucccgucuucc....................    3    1    pl2
......................................................................cacuccucuccucccgucuuUc....................    3    1    pl2
......................................................................cacuccucuccCcccgucuucc....................    4    1    pl2
......................................................................cacuccuUuccucccgucuucc....................    1    1    pl2
......................................................................cacuccucuGcucccgucuucc....................    3    1    pl2
......................................................................cacuccucuccucccgGcuucc....................    3    1    pl2
......................................................................cacuccucuccucccCucuucc....................    6    1    pl2
......................................................................cacuccucuccucccgucuCcc....................    4    1    pl2
......................................................................cacuccucuccuGccgucuucc....................    1    1    pl2
......................................................................Gacuccucuccucccgucuucc....................    3    1    pl2
......................................................................Nacuccucuccucccgucuucc....................    11   1    pl2
......................................................................cacuccucuccucGcgucuucc....................    2    1    pl2
......................................................................cacuccucuccucccgucuucc....................    5356 0    pl2
......................................................................cacuccucuccucccgAcuucc....................    8    1    pl2
......................................................................cCcuccucuccucccgucuucc....................    1    1    pl2
......................................................................cacuccucuccuUccgucuucc....................    5    1    pl2
......................................................................cacuccucuccucUcgucuucc....................    2    1    pl2
......................................................................cacuccucuccucccguUuucc....................    2    1    pl2
......................................................................cacuccucuccuccUgucuucc....................    5    1    pl2
......................................................................cacuccucuccucccgucuAcc....................    3    1    pl2
......................................................................cacuccNcuccucccgucuucc....................    20   1    pl2
......................................................................cacuccucuccucccgucuucA....................    55   1    pl2
......................................................................cacuccucuUcucccgucuucc....................    3    1    pl2
......................................................................cacuccucuccucccguGuucc....................    1    1    pl2
......................................................................caGuccucuccucccgucuucc....................    1    1    pl2
......................................................................cacuccucuccucccgCcuucc....................    7    1    pl2
......................................................................cacuccucucGucccgucuucc....................    1    1    pl2
......................................................................cacuccucuccucccgucCucc....................    1    1    pl2
......................................................................cUcuccucuccucccgucuucc....................    3    1    pl2
```

```
                               Star                                 Mature
agccgagucggagcugucggaggcgagggcgaggacgggaagagaggagggcgugguuucugcugguccucacuccucuccucccgucuuccuccuccuccucccauuccca
......................................................................caUuccucuccucccgucuucc....................     3     1     pl2
......................................................................cacuccucuccucccgAcuuccu...................     1     1     pl2
......................................................................cacuccucuccucccgGcuuccu...................     1     1     pl2
......................................................................cacCccucuccucccgucuuccu...................     1     1     pl2
......................................................................cacuccucuccucccgucuuccA...................     385   1     pl2
......................................................................cacuccucuccucccgucuuccG...................     4     1     pl2
......................................................................cacuccucuccucccgNcuuccu...................     2     1     pl2
......................................................................cacuccucuccucccgucuucAu...................     2     1     pl2
......................................................................cacuccNcuccucccgucuuccu...................     2     1     pl2
......................................................................cacuccucuccucccgucuucUu...................     1     1     pl2
......................................................................cacuccucuccucccgucuuccu...................     177   0     pl2
......................................................................cacuccucuccucccgucuuccuU..................     8     1     pl2
......................................................................cacuccucuccucccgucuuccuA..................     7     1     pl2
.......................................................................acuccucuccucccgucuucc....................     2     0     pl2
.........................................................................uccucuccucccgucuucc....................     2     0     pl2
..........................................................................ccucuccucccgucuucc....................     1     0     pl2
```
