# Supplementary material for: Integrative transcriptomic analysis uncovers the microRNA-centric regulation of Japanese encephalitis virus infection in porcine trophoblast cells
Source: Virulence. 2026 Jun 17;17(1):2690825. doi: 10.1080/21505594.2026.2690825 (PMC13313263; doi:10.1080/21505594.2026.2690825)
Supplement: FigShare.zip [file KVIR_A_2690825_SM3829.zip › FigShare/Original Western blot image.docx]

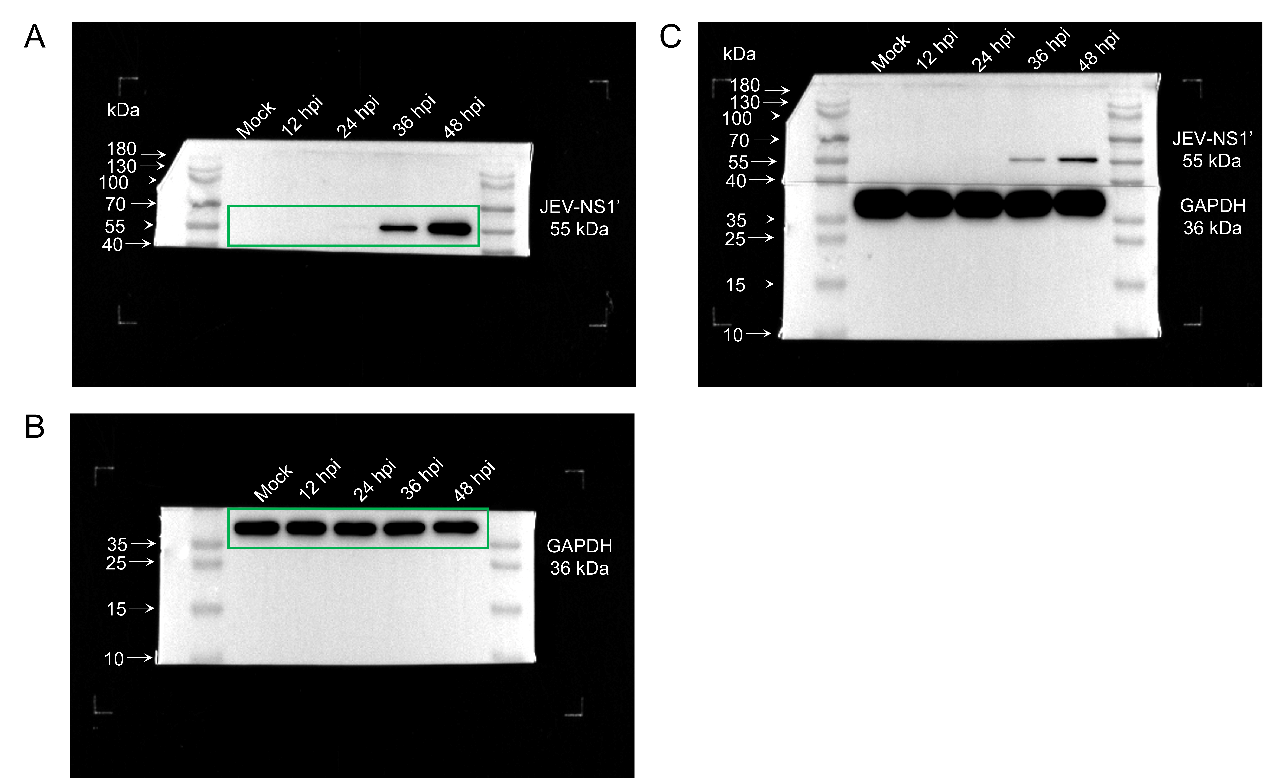


**Original Western blot image related to Figure 2D.** **(A,B)** The Western blots for JEV-NS1’ and GAPDH respectively, which match Figure 2D. **(C)** The original Western blot during the optimization of the exposure time. This figure demonstrates that JEV-NS1’ and GAPDH are of rather different protein abundance, which precludes optimal exposure of both proteins together. Therefore, we need to cut the whole membrane as two parts (each contains JEV-NS1’ and GAPDH, respectively), which were then exposed separately. To ensure that they both be exposed properly, we first performed a short exposure for GAPDH, and then performed a longer exposure for JEV-NS1’.


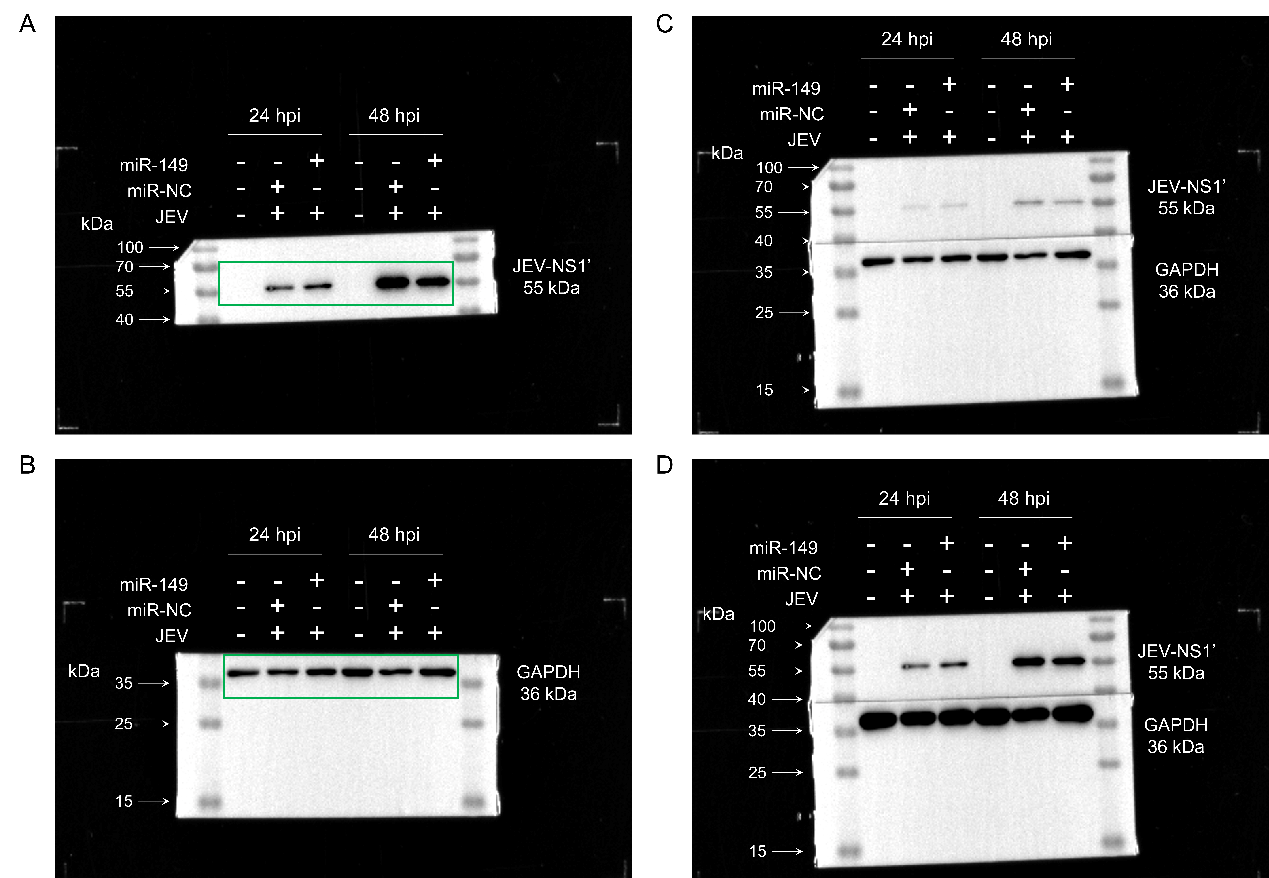


**Original Western blot image related to Figure 5E.** **(A,B)** The Western blots for JEV-NS1’ and GAPDH respectively, which match Figure 5E. **(C,D)** The original Western blot during the optimization of the exposure time. These figures demonstrate that JEV-NS1’ and GAPDH are of rather different protein abundance, which precludes optimal exposure of both proteins together. Therefore, we need to cut the whole membrane as two parts (each contains JEV-NS1’ and GAPDH, respectively), which were then exposed separately. To ensure that they both be exposed properly, we first performed a short exposure for GAPDH, and then performed a longer exposure for JEV-NS1’.


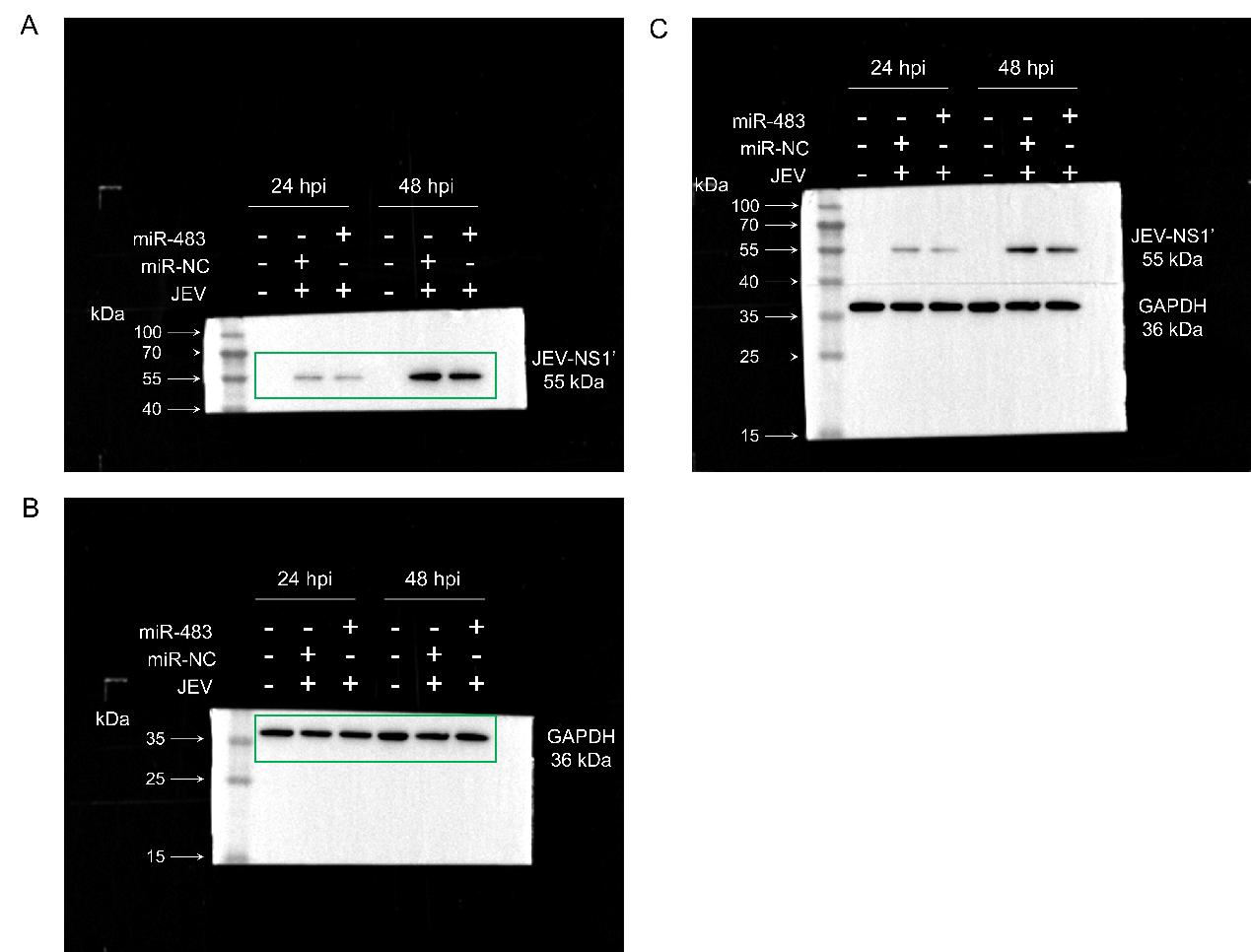


**Original Western blot image related to Figure 5F.** **(A,B)** The Western blots for JEV-NS1’ and GAPDH respectively, which match Figure 5E. **(C)** The original Western blot during the optimization of the exposure time. Although the protein abundances of JEV - NS1' and GAPDH in this experiment did not show as significant a difference as previously, in order to maintain consistency with the previous treatment methods, we still cut the whole membrane as two parts (each contains JEV-NS1’ and GAPDH, respectively), which were then exposed separately.
