# Supplementary material for: Integrative transcriptomic analysis uncovers the microRNA-centric regulation of Japanese encephalitis virus infection in porcine trophoblast cells
Source: Virulence. 2026 Jun 17;17(1):2690825. doi: 10.1080/21505594.2026.2690825 (PMC13313263; doi:10.1080/21505594.2026.2690825)
Supplement: FigShare.zip [file KVIR_A_2690825_SM3829.zip › FigShare/Supplementary tables and figures/figure-S2.pdf]

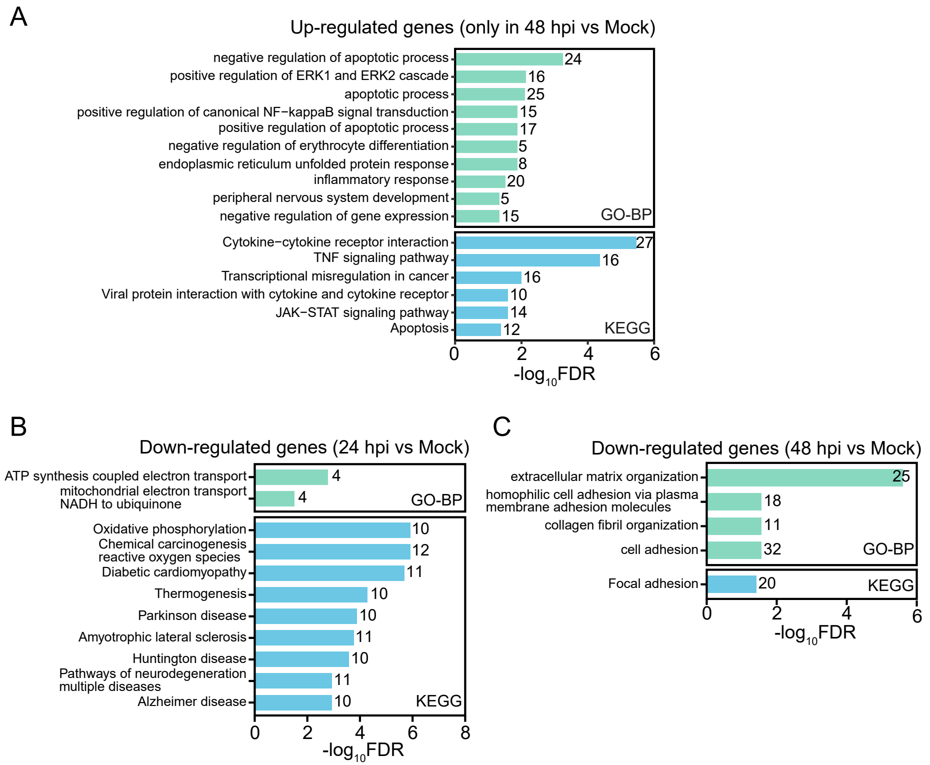
A
Up-regulated genes (only in 48 hpi vs Mock)
negative regulation of apoptotic process
positive regulation of ERK1 and ERK2 cascade
apoptotic process
positive regulation of canonical NF−kappaB signal transduction
positive regulation of apoptotic process
negative regulation of erythrocyte differentiation
endoplasmic reticulum unfolded protein response
inflammatory response
peripheral nervous system development
negative regulation of gene expression
24
16
25
15
17
5
8
20
5
15
GO-BP
Cytokine−cytokine receptor interaction
TNF signaling pathway
Transcriptional misregulation in cancer
Viral protein interaction with cytokine and cytokine receptor
JAK−STAT signaling pathway
Apoptosis
27
16
16
10
14
12
KEGG
0
2
4
6
-log10FDR
B
Down-regulated genes (24 hpi vs Mock)
ATP synthesis coupled electron transport
mitochondrial electron transport NADH to ubiquinone
4
4
GO-BP
Oxidative phosphorylation
Chemical carcinogenesis reactive oxygen species
Diabetic cardiomyopathy
Thermogenesis
Parkinson disease
Amyotrophic lateral sclerosis
Huntington disease
Pathways of neurodegeneration multiple diseases
Alzheimer disease
10
12
11
10
10
11
10
11
10
KEGG
0
2
4
6
8
-log10FDR
C
Down-regulated genes (48 hpi vs Mock)
extracellular matrix organization
homophilic cell adhesion via plasma membrane adhesion molecules
collagen fibril organization
cell adhesion
25
18
11
32
GO-BP
Focal adhesion
20
KEGG
0
2
4
6
-log10FDR
