# Supplementary material for: Integrative transcriptomic analysis uncovers the microRNA-centric regulation of Japanese encephalitis virus infection in porcine trophoblast cells
Source: Virulence. 2026 Jun 17;17(1):2690825. doi: 10.1080/21505594.2026.2690825 (PMC13313263; doi:10.1080/21505594.2026.2690825)
Supplement: FigShare.zip [file figure-S3.pdf › FigShare/Supplementary tables and figures/figure-S3.pdf]

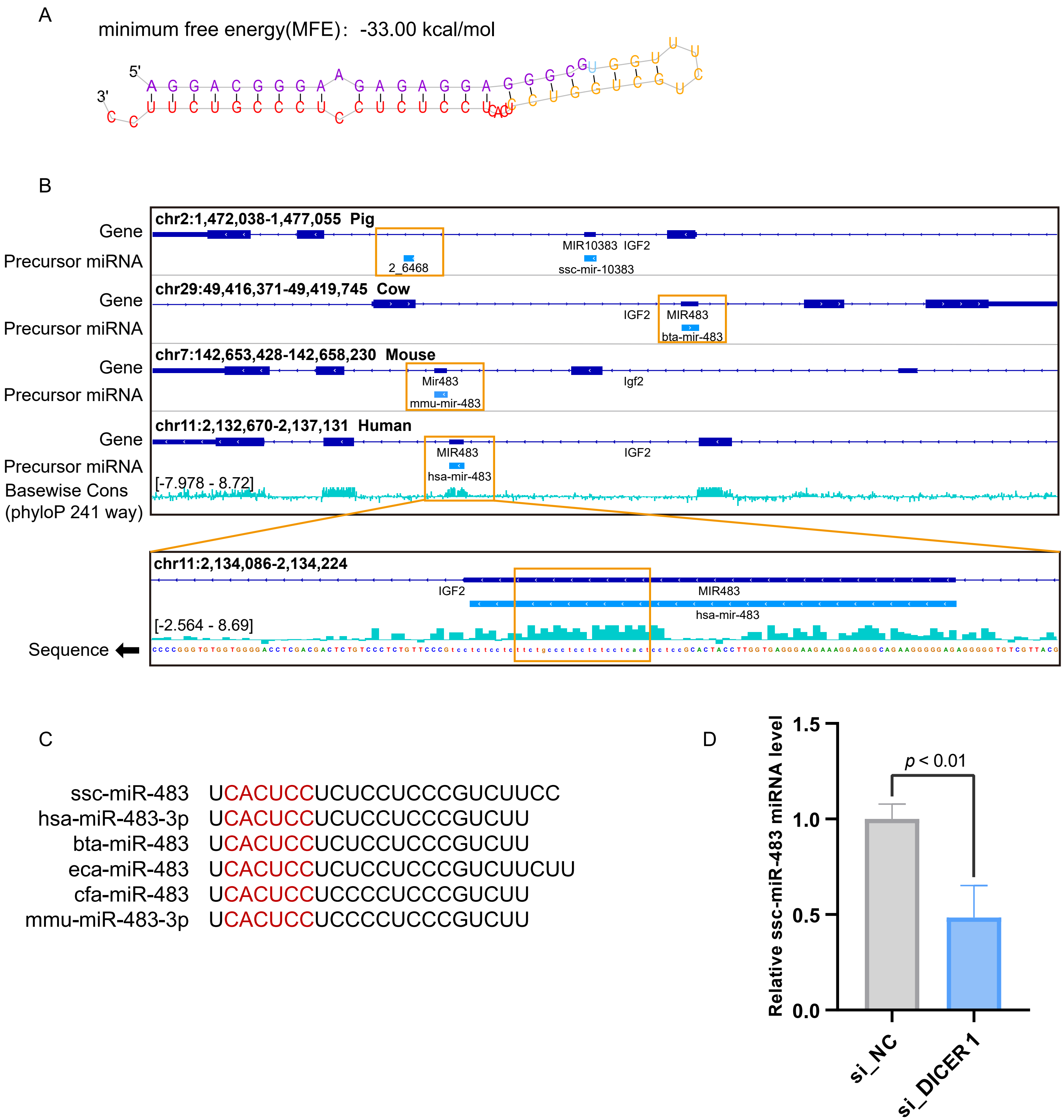
A
minimum free energy(MFE): -33.00 kcal/mol
5'
3'
B
chr2:1,472,038-1,477,055 Pig
Gene
Precursor miRNA
2_6468
MIR10383 IGF2
ssc-mir-10383
chr29:49,416,371-49,419,745 Cow
IGF2
MIR483
bta-mir-483
chr7:142,653,428-142,658,230 Mouse
Mir483
mmu-mir-483
Igf2
chr11:2,132,670-2,137,131 Human
MIR483
hsa-mir-483
IGF2
Basewise Cons
(phyloP 241 way)
[-7.978 - 8.72]
chr11:2,134,086-2,134,224
IGF2
MIR483
hsa-mir-483
[-2.564 - 8.69]
Sequence
C
ssc-miR-483 UCACUCCUCUCCUCCCGUCUUCC
hsa-miR-483-3p UCACUCCUCUCCUCCCGUCUU
bta-miR-483 UCACUCCUCUCCUCCCGUCUU
eca-miR-483 UCACUCCUCUCCUCCCGUCUUCUU
cfa-miR-483 UCACUCCUCCCCUCCCGUCUU
mmu-miR-483-3p UCACUCCUCCCCUCCCGUCUU
D
Relative ssc-miR-483 miRNA level
1.5
1.0
0.5
0.0
p < 0.01
si_NC
si_DICER1
